# Conceptual frameworks of barriers and facilitators to perinatal mental healthcare: the MATRIx models

**DOI:** 10.1192/bjo.2023.510

**Published:** 2023-07-13

**Authors:** Rebecca Webb, Elizabeth Ford, Abigail Easter, Judy Shakespeare, Jennifer Holly, Sally Hogg, Rose Coates, Susan Ayers, the MATRIx Study Team

**Affiliations:** Centre for Maternal and Child Health Research, School of Health Sciences, City, University of London, UK; Department of Primary Care and Public Health, Brighton & Sussex Medical School, UK; Department of Women and Children's Health, School of Life Course Sciences, King's College London, UK; and Section of Women's Mental Health, Institute of Psychiatry, Psychology & Neuroscience, King's College London, UK; retired general practitioner, UK; Section of Women's Mental Health, Institute of Psychiatry, Psychology and Neuroscience, King's College London, UK; Faculty of Education, University of Cambridge, UK

**Keywords:** Perinatal psychiatry, primary care, anxiety disorders, depressive disorders, post-traumatic stress disorder

## Abstract

**Background:**

Perinatal mental health (PMH) problems are a leading cause of maternal death and increase the risk of poor outcomes for women and their families. It is therefore important to identify the barriers and facilitators to implementing and accessing PMH care.

**Aims:**

To develop a conceptual framework of barriers and facilitators to PMH care to inform PMH services.

**Method:**

Relevant literature was systematically identified, categorised and mapped onto the framework. The framework was then validated through evaluating confidence with the evidence base and feedback from stakeholders (women and families, health professionals, commissioners and policy makers).

**Results:**

Barriers and facilitators to PMH care were identified at seven levels: individual (e.g. beliefs about mental illness), health professional (e.g. confidence addressing perinatal mental illness), interpersonal (e.g. relationship between women and health professionals), organisational (e.g. continuity of carer), commissioner (e.g. referral pathways), political (e.g. women's economic status) and societal (e.g. stigma). The MATRIx conceptual frameworks provide pictorial representations of 66 barriers and 39 facilitators to PMH care.

**Conclusions:**

The MATRIx frameworks highlight the complex interplay of individual and system-level factors across different stages of the care pathway that influence women accessing PMH care and effective implementation of PMH services. Recommendations are made for health policy and practice. These include using the conceptual frameworks to inform comprehensive, strategic and evidence-based approaches to PMH care; ensuring care is easy to access and flexible; providing culturally sensitive care; adequate funding of services and quality training for health professionals, with protected time to complete it.

Perinatal mental health (PMH) difficulties can occur during pregnancy or up to 12 months after birth. They commonly consist of anxiety disorders, depression, post-traumatic stress disorder and stress-related conditions such as adjustment disorder. Many disorders are comorbid.^1^ PMH difficulties can have a negative effect on women and their families.^2–7^ Furthermore, the cost to society is substantial, at approximately £8.1 billion for every annual cohort of women in the UK, with 72% of this cost attributable to the long-term impact on the child.^8^

The UK is an example of a country where PMH services are being prioritised and funded, but there is still a lot to learn. In the UK healthcare is free and mostly funded through taxation (the National Health Service (NHS)). Every year, the UK Government assigns a certain amount to be spent on healthcare. Each of the devolved nations (England, Wales, Scotland and Northern Ireland) receive a proportion of this funding and are able to decide which health services to allocate funds to.^9^ This means the amount and proportion of funds assigned to PMH services will differ between devolved nations. For example, in 2014 NHS England set out plans for £365 million to be spent on specialist PMH services from 2016 to 2021,^10^ as part of their ‘Five Year Forward View’. This has been continued and complemented by subsequent policy and funding announcements, including those in the NHS England ‘Long Term Plan’, which pledged an additional £2.3 billion a year, stating that by 2023–2024 66 000 women with moderate-to-severe mental health difficulties should have access to specialist care from preconception to 24 months postnatal.^11^ Scotland has invested £2.5 million in the Perinatal and Infant Mental Health Fund over 2.5 years, from 2020 to 2023.^12^ Between 2016 and 2019, Wales invested £1.5 million every year into PMH services,^13^ and between 2019 and 2022, Wales committed to implementing new community PMH services.^14^ Furthermore, in 2021, Northern Ireland announced the development of new specialist PMH services at an estimated cost of £4.7 million per year.^15^

Although there have been large improvements in PMH service provision in the UK since the publication of the these plans, in 2020 the Maternal Mental Health Alliance identified that 20% of clinical commissioning groups in England still did not have specialist PMH services. These gaps in specialist PMH service provision are even higher in Wales, Scotland and Northern Ireland, with 71.4% of health boards in Wales, 85.7% of health boards in Scotland and 100% of health and social care trusts in Northern Irelands not proving specialist community PMH services.^16^

## Treatment gaps

These treatment gaps may mean that women are not accessing the care that they need.^17^ Our recent systematic review of international research identified multiple levels (individual, health professional, interpersonal, organisational, political and societal) at which barriers to PMH care implementation can occur.^18^ Research also suggests that even if services are available, women do not always seek help^19^ or access help.^17,20^ Our meta-review of international research identifying barriers and facilitators to women accessing PMH care found that barriers occurred at the same multiple levels as implementation barriers.^21^ Both of these reviews provide an understanding of barriers to implementing PMH care and women deciding to seek help, accessing help and engaging in PMH care. Given the gaps in women accessing PMH care and PMH service implementation, as well the policy support and funding support for PMH services,^11,12,14,15,22^ it is both timely and important to provide evidence-based recommendations for policy and practice related to PMH service provision within the NHS context. To do this, the results from both reviews discussed above need to be synthesised, and the confidence with the evidence assessed, by creating a conceptual framework. A conceptual framework can be defined as a ‘network, or a plane, of interlinked concepts that together provide a comprehensive understanding of a phenomenon or phenomena’.^23^ The development of a conceptual framework can highlight areas for improvement and provide an empirical basis for recommendations for future practice and research.

## Method

### Aim

The aim of our study was to develop a conceptual framework of barriers and facilitators to PMH identification, assessment and treatment to inform healthcare services, practice and care pathways, and highlight where further research is needed.

### Ethics

This research was carried out using secondary data, therefore ethical approval and consent were not required. This study was registered with the International Prospective Register of Systematic Reviews, PROSPERO (review 1 identifier: CRD42019142854; review 2 identifier: CRD42019142854).

### Development of conceptual framework

**Fig. 1:**
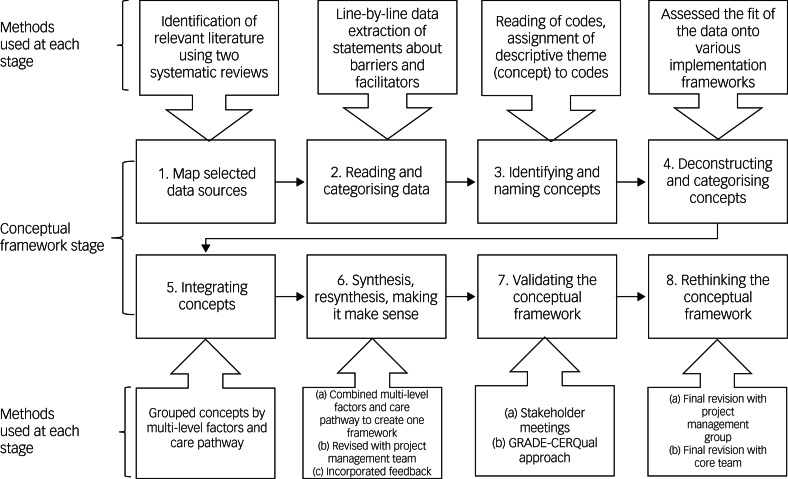
The process of developing the MATRIx conceptual frameworks.

The conceptual framework was developed with the method described by Jabareen^23^ (see Fig. 1). This involved following eight phases, which are presented in detail below.

#### Mapping the selected data sources

The first step is to identify sources of data, such as existing empirical data by using a systematic approach.^23^ To identify data, two systematic reviews were carried out according to a comprehensive search strategy and following the Preferred Reporting Items for Systematic Reviews and Meta-Analyses (PRISMA) guidelines.^24^ The first systematic review looked at barriers and facilitators to implementing PMH care, and 46 empirical studies were included.^18^ The second was a meta-review of systematic reviews, and looked at barriers and facilitators to women deciding to seek help, access help and engage in PMH care. A total of 32 systematic reviews were included.^21^ See individual papers for more detail of these reviews.

#### Extensive reading and categorising of the selected data

The aim of stage 2 is to read the selected data and categorise it by discipline.^23^ Line-by-line extraction of statements referring to facilitators or barriers to PMH assessment, care and treatment was carried out for both reviews. Therefore, data were categorised into barriers and facilitators.

#### Identifying and naming concepts

The aim of stage 3 is to read and reread the selected data and ‘discover’ concepts.^23^ This was done by re-reading the extracted data and assigning descriptive themes/concepts based on the meaning and content. Themes/concepts were developed and revised as each study was re-read.

#### Deconstructing and categorising the concepts

The aim of stage 4 is to identify each concept's attributes, characteristics and role, and organise and categorise the concepts accordingly.^23^ This stage was completed by assessing the fit of the data onto various implementation frameworks. For review 1, concepts were mapped onto an implementation model and a care pathway. Three implementation frameworks were assessed for their fit to the data: the Consolidated Framework for Implementation (CFIR^25^), the Reach Effectiveness Adoption Implementation Maintenance (RE-AIM^26^) and Ferlie and Shortell's Levels of Change framework.^27^ The RE-AIM and CFIR models both focus more on planning and evaluating the implementation of services. The data we were looking at were barriers and facilitators to implementation and women accessing services, thus a broader model was required. Ferlie and Shortell's Level of Change framework was originally developed to understand how health services could be improved, and so provided a better fit for our data. They hypothesise that four levels must change for health services to improve, and these are patient, care team, organisation and environment. The data fit best onto this model; however, certain concepts did not map onto this model. Therefore, the mapping of concepts was developed deductively from the initial theoretical framework, and then inductively revised as new concepts emerged.

#### Integrating concepts

The aim of stage 5 is to integrate and group together concepts that have similarities.^23^ The mapping of the concepts described previously led to the MATRIx multi-level model. This model has seven levels. The first level is the individual level, which reflects factors related to the person themselves. The second level is health professional, which reflects factors related to the health professional. Interpersonal refers to the relationship between women and health professionals, this is an extension of Ferlie and Shortell's work, and was included because this concept was apparent in the literature.^28^ The next concept is organisational, which relates to how the organisation is run, and the type of care the organisation delivers. The political level relates to the policies and governing that may affect women and healthcare. The societal level relates to larger societal factors, such as stigma (see Supplementary Appendix 1 available at https://doi.org/10.1192/bjo.2023.510). It is important to note that these levels do not exist in isolation, but often affect one another; for example, a lack of political funding and policy will have a negative impact on how an organisation is run.

Concepts were then mapped across different stages of the care pathway. The data best fit onto to an adapted care pathway from Goldberg and Huxley's Pathways to Care model.^28^ The MATRIx care pathway has eight stages (decision to consult, first contact with health professionals, assessment, deciding to disclose, referral, access to care, provision of optimal care, women's experience of care; see Supplementary Appendix 2). This care pathway is reflective of UK National Institute for Health and Care Excellence (NICE) guidelines for perinatal mental healthcare,^29^ and the introduction of Improving Access to Psychological Therapy services.

For review 2, concepts were mapped onto the MATRIx multi-level model and MATRIx care pathway.

#### Synthesis, resynthesis and making it all make sense

The aim in this phase is to synthesise concepts into a theoretical framework, using an iterative process of repetitive synthesis and resynthesis.^23^ This stage was done in multiple steps.

##### Combining the multi-level model and care pathway model

The MATRIx multi-level model and MATRIx care pathway model were combined together to create a draft framework. This had the care pathway along the top and the multi-level model down the side, with each concept placed in the corresponding box.

##### Revision with the project management group

Feedback on the draft framework was obtained from members of the project management group (A.E., Camilla Rosan, Agnes Hann, E.F., Fiona Alderdice, J.S. and S.A.). This included researchers and clinicians with expertise in maternal and child health, perinatal health and well-being, perinatal mental healthcare, strategy and transformation, and clinical psychology. All members work in the UK or NHS. Suggestions made by members of the project management group included considering the importance of outcome measurements, integration of different services, logistical issues such as co-location, and inclusion of a step between organisation and political structure (e.g. service managers).

##### Incorporating results

Another version of the draft framework was developed after incorporating the feedback from the previous step.

#### Validating the conceptual framework

The aim of this phase was to validate the framework.^23^ This was done in two steps: (a) stakeholder meetings to ascertain whether the proposed framework and its concepts made sense to practitioners and other stakeholders; and (b) assessing the confidence with the evidence. This step is in line with the development of NICE guidelines,^30^ where evidence is rated using the Grading of Recommendations Assessment, Development and Evaluation (GRADE)^31^ to assess the certainty of evidence before recommendations are made.

##### Stakeholder meetings

Following the approach of Leamy et al,^32^ three panels of stakeholders were consulted about the draft conceptual framework. Panels were held online via Microsoft Teams and led by members of the core team (J.S., S.A. and R.W.). The first panel comprised women, their partners and representatives from UK-based maternity charities that represent pregnant and postnatal women (e.g. National Childbirth Trust, Maternal Mental Health Change Agents). The second panel comprised healthcare professionals from different disciplines working for relevant NHS services. The third panel comprised NHS service commissioners and policy makers (see Table 1).

**Table 1:** Characteristics of stakeholders attending MATRIx stakeholder group meetings.

Stakeholder group	*n*	Role
Women and families	10	Mothers (*n* = 7) Fathers (*n* = 2) Not reported (*n* = 1) 8 reported that they had lived experience of perinatal mental illness
Health professionals	11	Specialist midwives (perinatal mental health, homelessness, substance misuse) (*n* = 5) Nurse/health visitor (*n* = 2) General practitioner (*n* = 2) Team manager, perinatal mental health (*n* = 1) Health research academic (*n* = 1)
Commissioners and policy makers	5	Implementation, training, project leads (*n* = 3) Clinical lead (*n* = 2)

During the stakeholder meetings, attendees were asked to review the conceptual framework and consider questions such as:

- How does the framework fit with your experience of implementing/accessing PMH Services?
- Does the framework include everything? Have we missed anything? What?
- In your view, what are the most important facilitators/barriers to implementing/accessing PMH services?
- In your view, what are the top recommendations for clinical practice?
- How can we disseminate this for most impact?

Conversations were recorded, and suggestions and recommendations were noted. These are summarised in Supplementary Appendix 3.

##### Using the GRADE-CERQual approach to assess confidence with the evidence

The GRADE-CERQual (Confidence in the Evidence from Reviews of Qualitative Research) approach was used to assess the confidence of the results for each of the concepts in the framework.^33^ To do this, empirical papers and feedback from stakeholders were assessed on their methodological limitations,^34^ coherence,^35^ adequacy of data^36^ and relevance of data^37^ for each concept (see Table 2).

**Table 2:** Rules followed for assigning confidence ratings to concepts

	High confidence	Medium confidence	Low confidence	Very low confidence
Methodology	Review 1: All domains were rated as high quality Review 2: High confidence – no or one non-critical flaw	Review 1: Most domains were rated as high quality Review 2: Moderate confidence – more than one non-critical flaw	Review 1: Most domains were rated as low quality Review 2: Low confidence – one critical flaw	Review 1: All domains were rated as low quality Review 2: Critically low confidence – more than one critical flaw
Coherence	All summaries were consistent in their content	Over half of the summaries were consistent in their content	Summary contents had a mix of two different themes	No consistency across summary contents
Adequacy	≥21 papers and more than half of the papers had thick data descriptions	10–20 papers and more than half of the papers had thick data descriptions	5–9 papers and/or fewer than half of the papers had thick data descriptions	<5 papers and/or fewer than half of the papers had thick data descriptions
Relevance	Review 1: Studies carried out in the UK/National Health Service Review 2: Reviews where >50% of included studies were carried out in the UK/National Health Service	Review 1: Studies carried out in Western countries, or countries with universal government funded healthcare (e.g. Canada) Review 2: Reviews where >50% of included studies met the above stipulation	Review 1: Lower-middle-income countries, or countries with universal health insurance coverage Review 2: Reviews where >50% of included studies met the above stipulation	Review 1: Countries without universal health insurance coverage (e.g. the USA) Review 2: Reviews where >50% of included studies met the above stipulation
Overall rating	Three or all aspects (methodology, coherence, adequacy, relevance) of a concept rated as high confidence	Three or all aspects of a concept rated as moderate confidence.	Three or all aspects of a concept rated as low confidence	Three or all aspects of a concept rated as very low confidence

Methodological limitations refers to the ‘extent to which there are concerns about the design or conduct of the primary studies that contributed evidence to an individual review finding’.^34^ Methodological limitations were assessed in two ways, and more details can be found in each individual paper.^18,21^ In brief, for review 1, the methodology sections of included studies were assessed for quality with the Joanna Briggs critical appraisal tools for qualitative research,^38^ cross-sectional studies,^39^ and text and opinion.^40^ For review 2, methodology sections of included reviews were appraised with the A Measurement Tool to Assess Systematic Reviews-2 (AMSTAR 2) criteria, which looks at factors such as the research question, search strategy and data extraction.^41^ See Supplementary Appendix 4 for the methodological limitations table.

Coherence refers to ‘how clear and cogent the fit is between the data from the primary studies and a review finding that synthesises that data’.^35^ Coherence was assessed by looking at the evidence assigned to that concept and identifying any outliers or ambiguous elements in the data. To do this, a summary from each of the papers included within a concept was written and outliers/ambiguous elements identified (see Supplementary Appendix 5).

Adequacy refers to the ‘overall determination of the degree of richness as well as the quantity of data to support a review finding’.^36^ Adequacy was assessed by looking at both the quantity and richness ('thickness’ and ‘thinness’) of the data for each concept. In the case of this research, a ‘thin’ description was defined as a set of statements rather than a description that provides the context of experiences and circumstances.^42^ An example of a thin description is this quote about healthcare professionals dismissing women's symptoms:^43^

‘[The study authors] found that women also felt that providers were downplaying the symptoms they were experiencing.’^43^

An example of a ‘thick’ description about the same topic is:

‘Having symptoms dismissed or attributed to factors other than PPD [postpartum depression] by healthcare professionals led to women ‘remaining silent’. Some women perceived that their difficulties would only be taken seriously when there were concerns about risk of harm to themselves or the infant. One woman said, “I kept going to this doctor and he used to give me a pep talk and send me home …”’.^44^

It is argued that the extent to which a text provides a thick description shows evidence of the authenticity of the results.^45^ See Supplementary Appendix 6 for the adequacy ratings.

Relevance refers to ‘the extent to which the body of data from the primary studies supporting a review finding is applicable to the context’.^37^ Relevance was assessed by identifying the country and health system of each study within a concept. This research was funded by the National Institute for Health and Care Research to develop recommendations for UK policy, so we defined direct relevance as studies carried out in the UK/NHS (or for review 2, where >50% of studies included in a systematic review were carried out in the UK/NHS). See Supplementary Appendix 7 for the geographical distribution of studies, and Supplementary Appendix 8 for relevance ratings.

For overall confidence rating, the confidence of each of these four aspects was rated by R.W. as high confidence, moderate confidence, low confidence or very low confidence. This meant that each concept had four specific confidence ratings. All four confidence ratings were then combined (see Table 2) to give an overall confidence rating for each concept. Where a concept had an even split of ratings and the ratings were next to each other in quality (e.g. high, high, medium, medium), the rating assigned to the ‘relevance’ of a concept was given a higher weighting. Where a concept had an even split of ratings, but the ratings were apart from each other in terms of quality (e.g. high, high, low low), the rating in the middle of these was given (e.g. medium). A decision was made to not assign anything higher than ‘low confidence’ to concepts where adequacy was given a ‘very low’ rating. This was to avoid putting too much emphasis on concepts where more research is needed. A decision was made to keep concepts that were rated as having ‘very low’ or ‘low’ confidence, and these were highlighted for future research. See Supplementary Appendix 9 for the overall confidence ratings of concepts.

#### Rethinking the conceptual framework

This step involved finalising the conceptual framework. This was done in two steps.

##### Final revision with the project management group

The most recent draft of the conceptual framework and the overall GRADE-CERQual rating for each concept was discussed with members of the project management group (A.E., E.F., Fiona Alderdice, Helen Cheyne, J.H., J.S., R.C., S.H. and S.A.). Feedback consisted of two main points. The first related to whether concepts with very low/low confidence ratings should be removed. As the majority of these concepts related to under-researched populations, removing them from the framework would perpetuate the cycle of underrepresentation of these groups. It was therefore decided to include all concepts in the framework, but provide an indication of the confidence rating scale. Recommendations for practice should be based on concepts with high/moderate confidence ratings, and recommendations for research should be based on concepts with low/very low confidence ratings.

The second point related to the language used. The framework presented was a framework of barriers, and it was decided that the negative language may act as a barrier itself. It was suggested that a framework of facilitators might also be appropriate and useful.

##### Final revision with the core team

For final revisions, members of the core team met for a 1-day workshop to consider all of the feedback given. It was agreed the following changes should be made.

- The decision to make two versions of the conceptual framework: one specifically related to barriers to PMH care, and the second related to facilitators to PMH care. The data were re-assessed, and barriers and facilitators were separated.
- The language of the two versions of the framework was scrutinised to remove blaming or negative language.
- Some of the healthcare-professional-level barriers and facilitators (e.g. training and heavy workloads) were moved to the service manager level. This is because the responsibility to provide this falls mostly within the service level rather than the healthcare professional level.
- Based on the funding structures in the UK, ‘funding complexities’ was moved to commissioner level, rather than government level. Although the government provides a set amount of funding for PMH, the complexities of using this funding most effectively to provide PMH services are more at the commissioner level.
- The framework was reviewed to ensure graphics and icons were representative and inclusive.

### Making recommendations for practice and future research for PMH assessment, care and treatment

Recommendations for practice and policy were developed from the conceptual frameworks. Where a concept had high or moderate confidence in the evidence, a recommendation to enact this concept in practice was made. This was first done by reframing the barriers into answers to the question ‘What would help to improve PMH identification, assessment and treatment?’, and by looking at the guidance provided by stakeholder groups in relation to recommendations (see Supplementary Appendix 3). Examples of good practice were also taken from the stakeholder consultation events, and from the FutureNHS Collaboration Platform. Where the confidence with the evidence was low or moderately low, recommendations for future research were made.

## Results

### Description of the conceptual frameworks

The two versions of the MATRIx conceptual framework were created to understand key barriers and facilitators to PMH identification, assessment, care and treatment, to improve PMH services. Syntheses of the reviews identified 78 key factors that can affect PMH care (see Supplementary Appendix 10). These are summarised in two versions of a conceptual framework, which provide pictorial representations of 66 barriers (see Supplementary Appendix 11) and 39 facilitators (see Supplementary Appendix 12) across the care pathway and at multiple levels (note that there is overlap with 27 of the barriers and facilitators.).

### Confidence in results

Of the 78 concepts identified, 14 were assigned a rating of high confidence with the evidence. Just under half of the concepts (*n* = 33) received a rating of moderate confidence. These will be discussed in more detail below. Slightly fewer (*n* = 25) concepts received a rating of low confidence, suggesting that more research is needed. These concepts included women's knowledge and understanding of the causes of mental illness, and where to go to seek help; demographic factors, such as the woman's ethnicity or current symptoms/diagnoses; healthcare professionals focusing too much on the infant; shared decision-making between women and healthcare professionals; co-location of buildings; care with a dedicated mental health champion and care that offers an opportunity to talk.

Only four concepts received very low confidence rating, suggesting that more research is needed into women's age or previous diagnoses/symptoms affecting help-seeking and access, the provision of supervision within organisations and organisational guidelines.

### Barriers

**Fig. 2:**
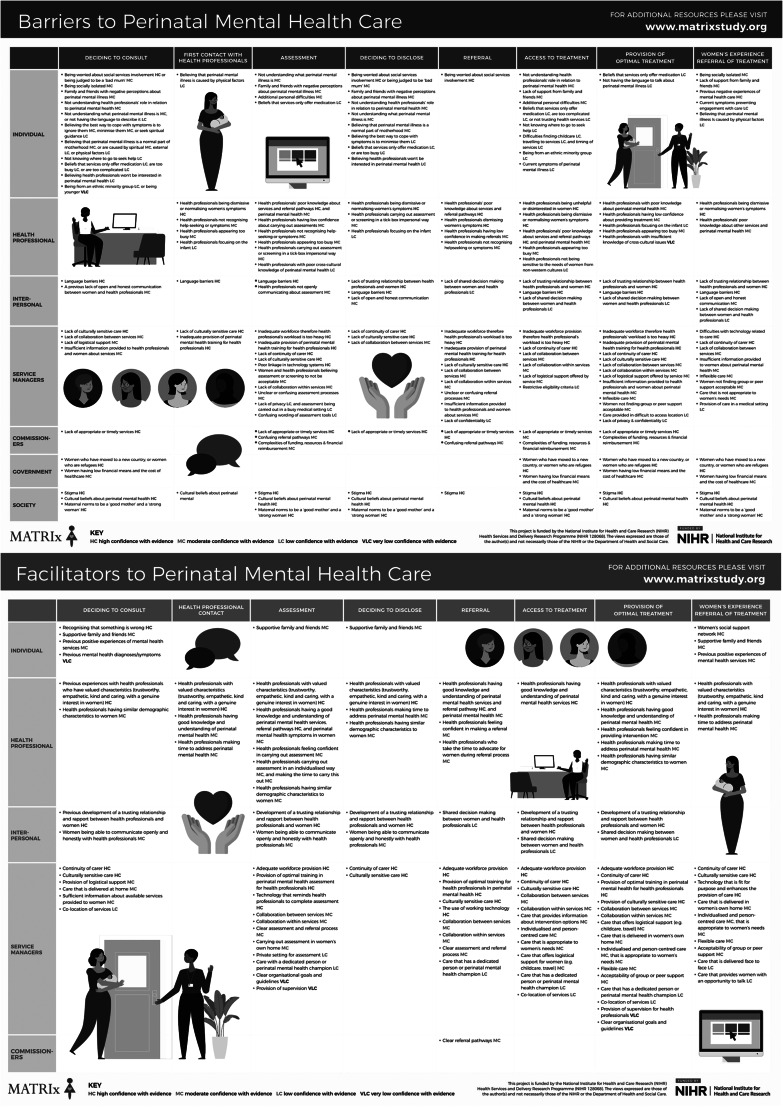
The MATRIx conceptual frameworks of barriers and facilitators to perinatal mental health care.

The conceptual framework for barriers to PMH care is shown in Fig. 2 and Supplementary Appendix 11 (in colour). Individual-level barriers with moderate and high confidence in the evidence included (in order of evidence confidence) being scared of social services involvement or being judged to be a ‘bad’ mum; having a lack of support from family and friends or them having negative perceptions about perinatal mental illness; being socially isolated; not understanding the role of the healthcare professional in relation to PMH; not understanding what perinatal mental illness is; believing that perinatal mental illness symptoms have physical causes, or are a normal part of motherhood; believing that the best way to cope with symptoms is to ignore them or minimise them; and previous negative experiences of mental healthcare.

Healthcare-professional-level barriers with moderate and high confidence included healthcare professionals being dismissive of or normalising women's symptoms, or not recognising help-seeking; appearing too busy; having poor knowledge about services, referral pathways and PMH in general; having low confidence about addressing PMH and carrying out assessment or screening in a tick-box or impersonal way.

Interpersonal-level barriers with moderate and high confidence were no trusting relationship between healthcare professionals and women, language barriers and a lack of open and honest communication.

At the organisational/service manager level, barriers with moderate and high confidence in the evidence were inadequate workforce resulting in healthcare professionals with a workload that is too heavy, inadequate provision of PMH training for healthcare professionals, lack of continuity of carer, lack of culturally sensitive care, difficulties with technology related to care, lack of collaboration within and between services, lack of logistical support offered by services, insufficient information provided about the care, inflexible care, care that is not appropriate for women's needs, confusing wording of assessment tools, assessment or screening viewed as unacceptable, and unclear or confusing assessment and referral processes within an organisation.

At the commissioner level, all three barriers had high or moderate confidence with evidence, and these were lack of appropriate or timely services; complexities of funding, resources and financial reimbursement; and confusing referral pathways.

Political-level barriers were rated as having moderate confidence with the evidence, and these were women being refugees or immigrants, the cost of healthcare and women's economic status. At the societal level, stigma, culture and maternal norms of being a ‘good mother’ and a ‘strong woman’ were all rated as having high confidence with the evidence.

### Facilitators

The conceptual framework for facilitators to PMH care is shown in Fig. 2 and Supplementary Appendix 12 (in colour). Fewer facilitators to PMH care were identified, which suggests more research is needed.

Individual-level facilitators with high or moderate confidence in the evidence were women recognising that something is wrong, having supportive family and friends and a strong support network. Previous positive experiences of mental health services were also a facilitator.

At the healthcare professional level, facilitators with high confidence were healthcare professionals possessing valued characteristics (e.g. being trustworthy, empathetic, kind, caring with a genuine interest, going above and beyond to meet women's needs), and healthcare professionals having knowledge of other services and referral pathways. Other facilitators with moderate confidence were healthcare professionals having similar demographics to women, having good knowledge and understanding of PMH, feeling confident in addressing PMH, making time to address PMH and carrying out assessment in an individualised way.

Interpersonal-level facilitators were the direct opposite of the barriers. Development of a trusting relationship and rapport between healthcare professionals, and women being able to communicate open and honestly with healthcare professionals, were both facilitators with moderate or high confidence in the evidence.

At the organisational/service manager level, facilitators with a high level of confidence were the provision of continuity of carer and culturally sensitive care for women, adequate workforce provision and provision of optimal training for healthcare professionals in PMH. Furthermore, technology that worked well and was fit for purpose was a facilitator for PMH care; for example, technology being easy to use without any bugs/glitches and being accessible to all who needed it. Facilitators with moderate confidence were individualised, person-centred, flexible care that is appropriate for women's needs and delivered face to face; the provision of logistical support for women; care that is delivered at home; group or peer support; sufficient information about available services; collaboration within and between services; and clear organisational assessment and referral processes.

At the commissioner level, one facilitator with moderate confidence was clear referral pathways.

### Recommendations

Detailed and specific recommendations can be found in Supplementary Appendix 13. They are split by level (e.g. individual, healthcare professional, etc.), with the concepts on the left and the related recommendation on the right. Before each recommendation is a numerical key indicating to whom this recommendation is addressed. Some of these recommendations are summarised below.

#### Recommendations for policy

Many elements of the conceptual frameworks can be modified by policy makers and government activity (e.g. workforce provision, healthcare capacity, training, etc.). Therefore, we recommend policy makers review the frameworks and take comprehensive, strategic and evidence-based efforts to ensure there is an effective system of PMH care.

Funding is required to ensure high-quality care provision. Therefore, the provision of a comprehensively researched and adequate budget is needed. Funding needs to be adequate for service needs and easily accessible. Funding structures may need to be revised depending on the needs of the community in which the service is delivered (e.g. affordable health insurance where free healthcare is not available). In some cases, it may be possible that funding has been provided, but services are not being implemented as effectively as possible.^18^ In these cases, health economics evaluations could be considered to ensure that health services are using funds appropriately, or to understand how funds could be used in a more effective way.^46^

The reduction of health inequalities is needed. It is therefore advisable that policy is put in place to improve equality in several areas: (a) between the genders by ensuring equal rights for women and men; (b) in terms of ethnicity, for example changes at the legislative level are needed to protect immigrants from being penalised for, or prevented from, accessing healthcare; and (iii) in terms of income, a fair and easily accessible welfare system is needed to prevent health inequalities based on deprivation.

#### Recommendations for practice

In terms of care, it is recommended that care is developed with women and is personalised and culturally appropriate. Increasing the flexibility and accessibility of services should be done through offering home visits and, where this is not possible, providing out-of-hours appointments located in an area with good transport links and an accessible building (e.g. ramps). In addition, service managers could consider the provision of virtual consultations using web-based platforms, but women should be given the choice about whether they would prefer virtual or face-to-face care.

Culturally sensitive care and increased accessibility of care is required for women who are unable to or have difficulty speaking the country's language. This can be done via pictorial aids, the purchase of products such as Language Line, or through collaboration with translation agencies. Where these tools are already available within a service, these should be utilised and additional time should be given for consultations with women where they are unable to, or have difficulty speaking the country's language.

Technology can be a facilitator for PMH services in terms of assessment, referral and intervention. Thus, technology systems should be co-produced with healthcare professionals and women to ensure ease of usability and integration into the workflow.

Within services, where not already implemented, multidisciplinary teams should be created to facilitate choice and personalised care and ensure an adequate workforce to meet women's needs. Culturally sensitive care could also be improved through the recruitment and retention of healthcare providers from diverse backgrounds.^47^ Silo working needs to be broken down, and service managers should encourage collaborative and joint working. The building of a coalition of health visitors, midwives, general practitioners, therapists, psychologists and psychiatrists is needed to encourage referral and reduce the risk of women falling out of the care pathway. Collaboration between services is also needed, with a focus on the identification and building of working relationships and networks with other services (e.g. Citizens Advice Bureau).

Healthcare professionals should be provided with high-quality training that is delivered face to face and incorporates role-play simulators where appropriate. This should include training in cultural sensitivity and cross-cultural mental health. Training time for healthcare professionals should be built into workloads and protected. Ideally, training should be provided as part of health professionals’ qualification training, not just afterward. Furthermore, there is a move within countries, such as the UK, to provide care that is trauma informed. This means care that recognises the impact of trauma and works to prevent re-traumatisation.^48^ Given the association between trauma and mental health,^49^ it is important that trauma-informed training is considered.

#### Recommendations for research

Future research should focus on addressing the concepts assigned low and very low confidence within this project, e.g. women's beliefs about the causes of mental illness and how this may affect help-seeking. Outside of the perinatal period, it has been suggested that within Western society, the biomedical approach to mental health provision can be a barrier to mental healthcare, as it is not sensitive to different cultural constructions of mental health problems.^50^ Furthermore, research suggests that treatments that diverge from standard Western treatments (e.g. meaning making, spiritual treatments, narrative or story telling interventions) can be helpful to individuals by improving their quality of life^51^ and reducing trauma symptoms.^52,53^ Future research should investigate if this is the case in perinatal populations.

At the healthcare professionals and interpersonal level, research should look at whether healthcare professionals focusing primarily on the infant is a barrier to PMH care, and if shared decision-making between women and healthcare professionals is a facilitator. At the organisational level, future research should investigate the impact of co-location of buildings, care with a dedicated mental health champion, care that offers an opportunity to talk, the provision of supervision within organisations, and organisational guidelines on PMH care access and implementation.

## Discussion

### Summary of main findings

Syntheses of the evidence identified 78 key factors that can affect PMH care. These are summarised in two conceptual frameworks, which provide pictorial representation of 66 barriers and 39 facilitators across the care pathway and at multiple levels. These frameworks were used to provide evidence-based recommendations for national health service policy, practice and research.

### Relevance to the wider literature

Concepts identified on all levels of the conceptual framework are supported by research carried out with the wider population. For example, at the individual level, previous research has identified the following as barriers to help-seeking: poor mental health literacy in young people and adults,^54,55^ fear of judgement or consequences of help-seeking/disclosure in adults,^56^ and poor social support in those who self-harm.^57^ At the healthcare professional level, non-perinatal populations report negative attitudes from healthcare professionals in relation to their mental illness, or being dismissed or not listened to by healthcare professionals.^58,59^ Dismissive and negative attitudes from healthcare professionals are likely stem from societal issues such as stigma,^60,61^ but also commissioner level issues such as poor training^61^ and a heavy workload resulting from understaffing.^62,63^ At the interpersonal level, other research suggests communication with healthcare professionals influences individuals’ experiences of mental healthcare.^64,65^ At the organisational level, other research suggests appropriateness of care is important for keeping people engaged.^55,66–68^ At the commissioner level, previous research has found a lack of services^69–71^ and long delays between referral and start of treatment^72^ are barriers to care. Political factors such as economic status, income and the cost of healthcare were barriers to PMH care. Multiple studies have found that these political factors can exacerbate mental health difficulties^73,74^ and affect help-seeking^71,75,76^ for mental health problems. Finally, at the societal level, previous research has also found stigma is one of the leading barriers to help-seeking in non-perinatal populations.^56,77,78^

### Strengths and limitations

The MATRIx conceptual frameworks have multiple strengths. First, a comprehensive method, which involved following a systematic process, was used for their development. Next, further validation of the MATRIx conceptual frameworks was carried out in multiple stages, with both stakeholder perspectives and confidence of evidence (GRADE-CERQual approach^33^ taken into account. Finally, the MATRIx conceptual frameworks were extensively revised based on the feedback received during the validation stage, showing the appropriateness of these models to both the evidence and NHS stakeholder experience.

However, there are several limitations to the MATRIx conceptual frameworks. The research analysed during the development of the conceptual frameworks related specifically to women and mothers. A decision was made to not look at the research including fathers because this is a topic that needs investigating in its own right. This means that the results from this review may not be generalisable to fathers, partners and families. These reviews also excluded services for substance misuse because these disorders raise unique challenges in terms of assessment and treatment that may not be generalisable to other disorders. Similarly, although we included international research when developing our conceptual frameworks, we rated studies and reviews carried out in low- and middle-income countries and countries without universal access to healthcare as being less relevant during the GRADE-CERQual evaluations. This means our recommendations are unlikely to be universally relevant. Another limitation is that when identifying literature, only reviews published in academic journals and written in the English language were included. Relevant reviews from health services, charities, third-sector organisations and other grey literature may have been missed. Further, the use of GRADE-CERQual to evaluate confidence in the findings is a strength, but ratings were done by one researcher (R.W.), which may mean they are slightly less valid. However, the GRADE-CERQual approach is described thoroughly, and specific rules for each of the assessments were discussed and agreed with the research team to ensure ratings were standardised.

### Recommendations for future research and practice

There were some limitations in terms of the evidence identified to inform the conceptual frameworks, and therefore future research should address this. These include (a) identifying more facilitators to PMH care, as most of the research focused on barriers; (b) understanding barriers and facilitators based on the severity of illness and different PMH difficulties; (c) barriers and facilitators to PMH in universal, primary care or in-patient care; (d) research with more diverse populations, including the transgender community; (e) research carried out in lower-middle income countries and (f) feedback from members of the research programme management group introduced the idea of incorporating service outcome measurements into the conceptual framework (stage 6b). This was not identified from the literature and may reflect the nature of service commissioning in the NHS, where services need to show that they are effective in order to be recommissioned. Thus, outcome measures to evaluate services need further attention.

In conclusion, the MATRIx framework led to the development of evidence-based recommendations for practice and commissioning (Supplementary Appendix 13). Despite being aimed at different stakeholder groups, these recommendations are all highly intertwined, and the uptake of one would be likely to have positive effects on others; for example, the continuation of prioritising funding for PMH services will affect the amount that service commissioners can allocate to PMH services. This should affect the workforce, increasing opportunities for continuity of carer models, staff training and other resources such as translators and logistical support.

## Data Availability

The data that support the findings of this study are available from the corresponding author, R.W., upon reasonable request.

## Supplementary material

For supplementary material accompanying this paper visit http://doi.org/10.1192/bjo.2023.510. click here to view supplementary material
