# Supplementary material for: Conceptual frameworks of barriers and facilitators to perinatal mental healthcare: the MATRIx models
Source: BJPsych Open. 2023 Jul 13;9(4):e127. doi: 10.1192/bjo.2023.510 (PMC10375902; doi:10.1192/bjo.2023.510)
Supplement: Supplementary file 1 [file S2056472423005100sup001.docx]

**The MATRIx Model – Conceptual frameworks of barriers and facilitators to implementing and women accessing perinatal mental health care services**

**Supplementary Materials**

[*Supplementary Materials 1:* The MATRIx Multi-Level Model 2](#_Toc108781720)

[*Supplementary Materials 2*: The MATRIx Care Pathway 3](#_Toc108781721)

[*Supplementary Materials 3*: Stakeholder group suggestions for conceptual framework, practice and research 4](#_Toc108781722)

[*Supplementary Materials 4:* GRADE-CERQual Methodological quality table 8](#_Toc108781723)

[*Supplementary Materials 5:* GRADE-CERQual Coherence rating table 25](#_Toc108781724)

[*Supplementary Materials 6:* GRADE-CERQual Adequacy rating table 43](#_Toc108781725)

[*Supplementary Materials 7:* Geographical distribution of research 65](#_Toc108781726)

[*Supplementary Materials 8.* GRADE-CERQual Relevance rating table 67](#_Toc108781727)

[*Supplementary Materials 9:* GRADE-CERQual overall confidence of concepts table 87](#_Toc108781728)

[*Supplementary Materials 10:* Identified barriers and facilitators to perinatal mental health care 102](#_Toc108781729)

[*Supplementary Materials 11:* MATRIx barriers conceptual framework 105](#_Toc108781730)

[*Supplementary Materials 12:* MATRIx facilitators conceptual framework 106](#_Toc108781731)

[*Supplementary Materials 12:* MATRIx recommendations for policy and practice 107](#_Toc108781732)

[*Supplementary Materials 13:* References 121](#_Toc108781733)

# *Supplementary Materials 1:* The MATRIx Multi-Level Model


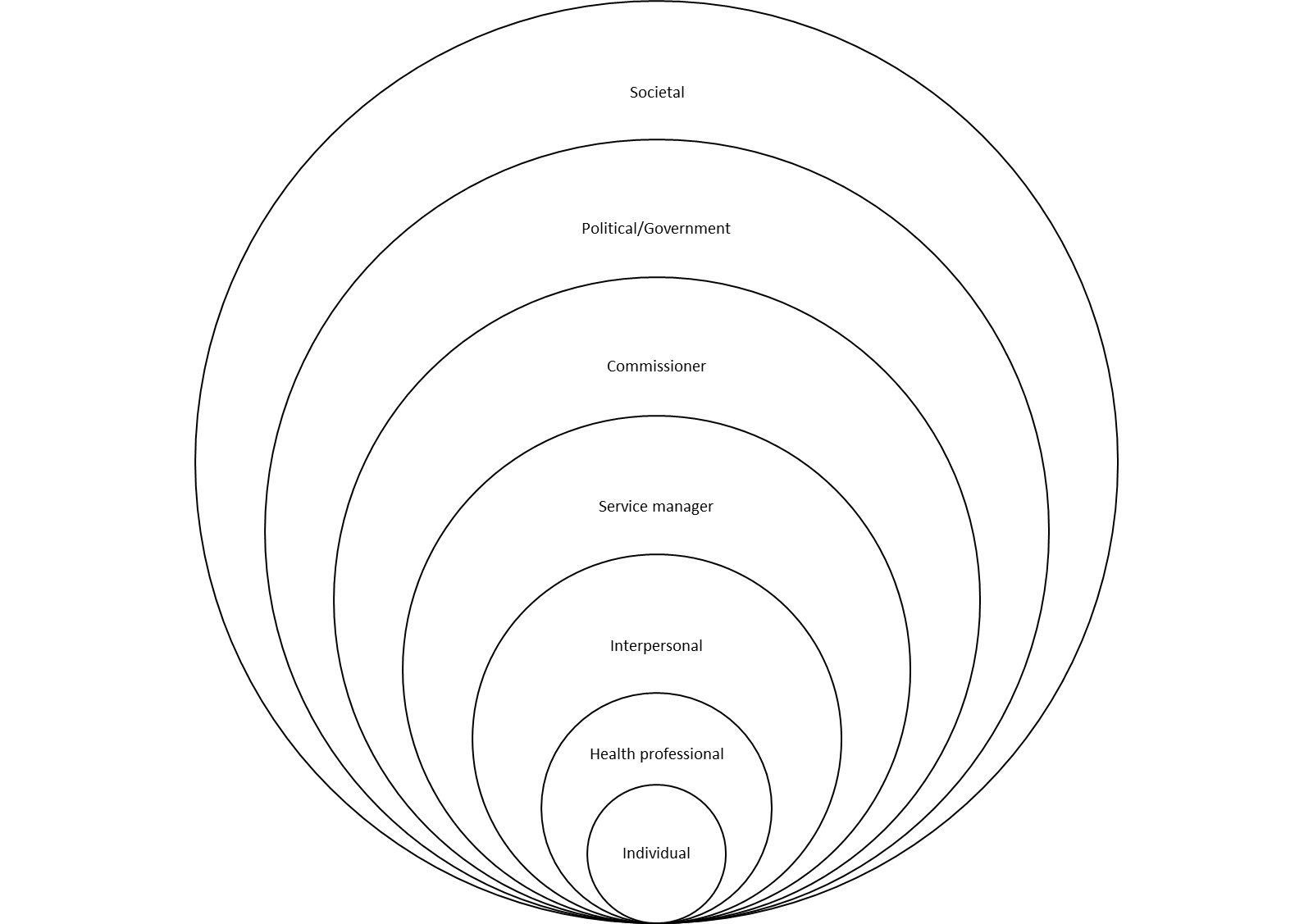


# *Supplementary Materials 2*: The MATRIx Care Pathway


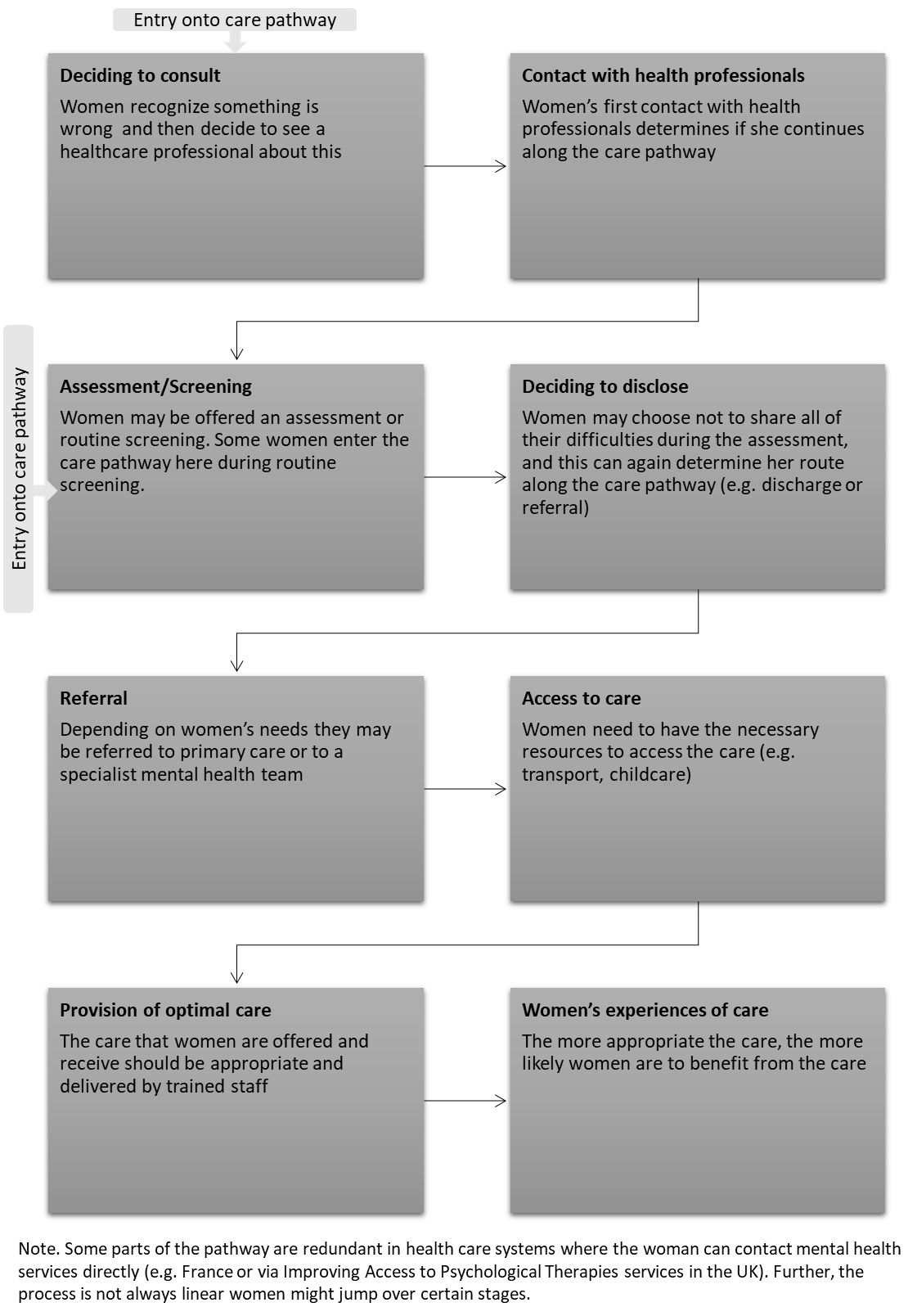


# *Supplementary Materials 3*: Stakeholder group suggestions for conceptual framework, practice and research

| **Women and families** | **Health professionals** | **Policy makers and commissioners** |
| --- | --- | --- |
| **Conceptual framework** | | |
| - Use inclusive, parent focused language e.g., parents-to-be - Avoid the use of “illness” or “problem” - Make infant-centred, whole-family approach rather than woman-centred,. Means all co-parents will be involved. - Think about how to highlight peer support, not just the clinical pathway. - Be more specific about funding - Visualisations to be inclusive (e.g. same sex couples, mothers with disabilities). | - Consider the gap in services between adjustment difficulties and severe PMH difficulties - Barriers also include variation between different areas | - Societal level – PMH difficulties are not just depression. Need to change the narrative - Consider the audience – UK vs international vs England only - Consider crisis and liaison services, maternal MH hubs, home based treatment teams, out of hours, paediatrics - Add barriers related to fragmentation of funding pots |
| **Practice** | | |
| Training   - Training for all people who come into contact with women and families (consultants, receptionists) - Uptake for training more likely if face to face - Training to include: - Language used (diagnostic labels not always helpful, every person is different) - Health inequalities - PMH is not just postnatal depression and does not always mean poor bonding - Different family structures - Lived experience stories (but protect those telling the stories) | Training   - Ring fenced times/time protected - Accreditation, matched to competencies - Mandatory PMH training for all, pitched at appropriate level - Dedicated person or network to deliver training - Training should cover - How to talk about PMH, what questions to ask. - Know where to refer to, how to fill out referral forms - Diversity of families (e.g. race, culture, family structure) - Treatment, including medication - Vulnerable groups | Training   - Time protected, funding backfills for time to attend and deliver training - Not e-learning – delivered face to face - Co-produced with families with lived experience - Practical |
| - Service provision - Whole family approach - Continuity of carer throughout entire perinatal period - Face to face information provision as well as leaflets - Make every contact count throughout entire care pathway. - Joined up working and integrated services, do not leave out parents whose infants are in the neonatal intensive care unit, and those experiencing perinatal loss - Transparency of pathways to users | - Service structure - Continuity of carer - Make every contact count - Trauma informed - More time needed at appointments - Engaging with diverse families - Pictorial assessment, translation tools. Availability of translators | - Service structure - Integration of adult/acute mental health services - Champions who are really invested |
|  | Stakeholder specific recommendations - Silos   - Communication within and between teams - Regular team meetings with HPs from different disciplines - Using a “contact us anytime” approach - Culture of team working, joint working, sharing knowledge, approachable. - Developing relationships across disciplines. - Co-location - Stakeholder specific recommendations – IT - All use same system, or communication across systems - Liaison person who has access to all systems - Referral systems that allow for one referral form that HPs can complete and that is sent to one place where it can then be triaged. | - Stakeholder specific recommendations - Commissioning - Increasing commissioners understanding and views of PMH, sustainability at a commissioning level - Funding - to be pulled from all areas, not just ringfenced as it is everyone’s business. Fragmentation of funding pots needs to be reduced - Space – physical building space, especially to enable integration across teams |
| **Research** | | |
| - Research with people with lived experiences about the impact of sharing stories |  | - Harder to reach women, more detailed look at their barriers at decision to consult level. - A framework for barriers and facilitators to implementing PMH for workforce alone e.g. why is training a barrier and how to overcome it? |

# *Supplementary Materials 4:* GRADE-CERQual Methodological quality table

| **Theme** | **Definition of theme** | **Studies citing this theme** | **N** | **No methodological concerns**  **(High confidence)** | **Minor concerns**  **(Moderate confidence)** | **Moderate concerns**  **(Low confidence)** | **Serious concerns**  **(Very low confidence)** | **Overall methodological confidence rating*** |
| --- | --- | --- | --- | --- | --- | --- | --- | --- |
| **1 Women** | | | | | | | | |
| **1.1 Beliefs about health services** | | | | | | | | |
| 1.1.1 Services only offer medication | The belief that health services will only offer medication to treat PMH concerns | *Doering et al., 2017; Ganann et al., 2019; Williams et al., 2016; Young et al., 2019;*  Bina, 2020; Button et al., 2017; Dennis & Chung-Lee, 2006; Hadfield & Wittkowski, 2017; Jones, 2019; Megnin-Viggars et al., 2015; Nilaweera et al., 2014; Sorsa et al., 2021; Tobin et al., 2018 | 14 | *Doering*  *Young* | *Ganann*  *Williams*  Hadfield 17  Tobin | Bina 20  Button  Jones 19  Megnin-Viggars  Nilaweera  Sorsa | Dennis | **Moderate confidence –** majority of studies lower quality |
| 1.1.2 Services are stretched | The belief that PMH services are too stretched and will therefore be unable to help women | Dennis & Chung-Lee, 2006; Hadfield & Wittkowski, 2017 | 2 |  | Hadfield |  | Dennis | **Low confidence –** middle of minor and serious |
| 1.1.3 Services are too complicated | Services being too complex or complicated | *Ganann et al., 2019*  Tobin et al., 2018  SH | 2 |  | *Ganann*  Tobin |  |  | **Moderate confidence –** all have minor methodological concerns |
| 1.1.4 Women’s mistrust and fear of services | Having little trust in health services | *Boyd et al., 2011*  Jones, 2019 | 2 |  | *Boyd* | Jones 19 |  | **Moderate confidence* -** even split, rounded up |
| **1.2 Beliefs about HCPs** | | | | | | | | |
| 1.2.1 Not understanding HCPs’ role | Not understanding the roles of HCPs and how their roles related to PMH | Brealey et al., 2010; Button et al., 2017; Dennis & Chung-Lee, 2006; Hadfield & Wittkowski, 2017; Hewitt et al., 2009; Megnin-Viggars et al., 2015; Mollard et al., 2016; Morrell et al., 2016; Nilaweera et al., 2014; Sambrook Smith et al., 2019; Schmied et al., 2017; Scope et al., 2017 | 12 | Hewitt  Morrell | Hadfield 17  Sambrook-Smith | Button  Megnin-Viggars  Mollard  Nilaweera  Schmied  Scope | Brealey  Dennis | **Low confidence -**  most reviews have moderate methodological concerns |
| 1.2.2 Believing HCPs won’t be interested | Believing HCPs won't be interested in PMH | Bina, 2020; Hadfield & Wittkowski, 2017 | 2 |  | Hadfield 17 | Bina 20 |  | **Moderate confidence* -** even split, round up because all SRs |
| **1.3 Beliefs about perinatal mental illness** | | | | | | | | |
| **1.3.1 What is it?** | | | | | | | | |
| 1.3.1.1 What is perinatal mental illness? | Having poor or no knowledge about PMI | *Atif et al., 2019; Kerker et al., 2018*  Bina, 2020; Button et al., 2017; Dennis & Chung-Lee, 2006; Hadfield & Wittkowski, 2017; Hansotte et al., 2017; A. Jones, 2019; Lucas et al., 2019; Megnin-Viggars et al., 2015; Morrell et al., 2016; Newman et al., 2019; Sambrook Smith et al., 2019; Schmied et al., 2017; Scope et al., 2017; Staneva et al., 2015; Tobin et al., 2018; Watson et al., 2019 | 18 | Lucas  Morrell | Hadfield 17  Sambrook-Smith  Tobin  Watson | *Atif 19*  *Kerker*  Bina 20  Button  Hansotte  Jones 19  Megnin-Viggars  Newman  Schmied  Scope | Dennis | **Low confidence -**  most of the reviews had moderate methodological concerns |
| 1.3.1.2 No language to describe perinatal mental illness | Not having the language to describe PMI | *Bina et al., 2018*  Brealey et al., 2010; Staneva et al., 2015; Tobin et al., 2018; Watson et al., 2019 | 5 |  | *Bina 2018*  Staneva  Tobin  Watson |  | Brealey | **Moderate confidence -**  most reviews have minor methodological concerns |
| **1.3.2 Causes of perinatal mental illness** | | | | | | | | |
| 1.3.2.1 Spiritual/cultural causes | Believing that symptoms are caused by cultural or spiritual factors | *Atif et al., 2016; McCauley et al., 2019; Nakku et al., 2016*  Button et al., 2017; Schmied et al., 2017; Wittkowski et al., 2014 | 6 | *McCauley* | *Atif 16*  *Nakku* | Button  Schmied | Wittkowski | **Moderate confidence -**  reviews are of lower quality, but generally more rigorous than 1 primary study |
| 1.3.2.2 External causes | Believing that symptoms are caused by external factors such as jobs, being a migrant | Bina, 2020; Button et al., 2017; Dennis & Chung-Lee, 2006; Lucas et al., 2019; Schmied et al., 2017; Staneva et al., 2015; Tobin et al., 2018; Watson et al., 2019 | 8 | Lucas | Staneva  Tobin  Watson | Bina 20  Button  Schmied | Dennis | **Moderate confidence -**  even split, given the higher rating because they are all SRs |
| 1.3.2.3 Physical causes | Believing that symptoms are caused by physical factors such as tiredness and hormones | *O’Mahen & Flynn, 2008*  Bina, 2020; Button et al., 2017; Dennis & Chung-Lee, 2006; Forde et al., 2020; C. C. G. Jones et al., 2014; Newman et al., 2019; Sambrook Smith et al., 2019; Schmied et al., 2017; Staneva et al., 2015; Watson et al., 2019 | 13 | *O’Mahen* | Forde  Sambrook-Smith  Staneva  Watson | Bina 20  Button  Newman  Schmied | Dennis  Jones 14 | **Low confidence -**  most SRs have moderate methodological concerns |
| 1.3.2.4 A normal response to motherhood? | Believing symptoms are just a normal response to motherhood | *Williams et al., 2016*  Dennis & Chung-Lee, 2006; Giscombe et al., 2020; Jones et al., 2014; Sambrook Smith et al., 2019; Schmied et al., 2017; Slade et al., 2020; Sorsa et al., 2021; Viveiros & Darling, 2018 | 9 |  | *Williams*  Sambrook-Smith  Slade | Schmied  Sorsa  Viveiros | Dennis  Giscombe  Jones 14 | **Low confidence** - most SRs have moderate methodological concerns |
| **1.3.3 How to cope with symptoms** | | | | | | | | |
| 1.3.3.1 Ignore them | Women many deal with symptoms by ignoring them and assuming they will go away on their own | Bina, 2020; Hadfield & Wittkowski, 2017; Jones et al., 2014; Newman et al., 2019; Schmied et al., 2017; Slade et al., 2020 | 6 |  | Hadfield 17  Slade | Bina 20  Newman  Schmied | Jones 14 | **Low confidence –**  most have moderate methodological concerns |
| 1.3.3.2 Seek spiritual guidance | Women may cope with symptoms by seeking spiritual guidance | Hansotte et al., 2017; Kassam, 2019; Schmied et al., 2017; Watson et al., 2019 | 4 |  | Watson  Kassam | Hansotte  Schmied |  | **Moderate confidence* -** even split, round up because all SRs |
| 1.3.3.3 Minimise them | Women may minimise or deny their symptoms | *Shakespeare et al., 2003*  Bina, 2020; Dennis & Chung-Lee, 2006; Forde et al., 2020; Hewitt et al., 2009; Holopainen & Hakulinen, 2019; Jones et al., 2014; Kassam, 2019; Megnin-Viggars et al., 2015; Schmied et al., 2017; Slade et al., 2020; Staneva et al., 2015; Tobin et al., 2018; Watson et al., 2019 | 14 | Hewitt  Holopainen | *Shakespeare*  Forde  Kassam  Slade  Staneva  Tobin  Watson | Bina 20  Megnin-Viggars  Schmied | Dennis  Jones 14 | **Moderate confidence -**  most SRs have minor methodological concerns |
| **1.4 Deciding to seek help** | | | | | | | | |
| 1.4.1 Recognising something is wrong | The first step to seeking help for many women, was recognising that something is "wrong" | Bina, 2020; Button et al., 2017; Forde et al., 2020; Hadfield & Wittkowski, 2017; Slade et al., 2020; Staneva et al., 2015; Viveiros & Darling, 2018 | 8 |  | Forde  Hadfield 17  Slade  Staneva | Bina 20  Button  Viveiros |  | **Moderate confidence -** most SRs have minor methodological concerns |
| 1.4.2 Where do I go to seek help? | The next step to help seeking is then understanding where to go in order to seek help | *Ganann et al., 2019*  Bina, 2020; Dennis & Chung-Lee, 2006; Hansotte et al., 2017; Megnin-Viggars et al., 2015; Schmied et al., 2017; Sorsa et al., 2021; Tobin et al., 2018 | 9 |  | *Ganann*  Tobin | Bina 20  Hansotte  Megnin-Viggars  Schmied  Sorsa | Dennis | **Low confidence -** most have moderate methodological concerns |
| **1.5 Fear of judgement** | | | | | | | | |
| 1.5.1 Fear of being seen as a bad mum | Fear of being judged and being seen as a bad mother | Bina, 2020; Brealey et al., 2010; Button et al., 2017; Forde et al., 2020; Jones et al., 2014; Lucas et al., 2019; Slade et al., 2020; Sorsa et al., 2021; Viveiros & Darling, 2018 | 9 | Lucas | Forde | Bina 20  Button | Brealey  Jones 14 | **Low confidence -**  most have moderate or serious methodological concerns |
| 1.5.2 Social services/ removal of child | Fear of social services involvement or their child being removed from their care | *Boyd et al., 2011; Feinberg et al., 2006; Shakespeare et al., 2003; Young et al., 2019*  Bina, 2020; Brealey et al., 2010; Button et al., 2017; Dennis & Chung-Lee, 2006; Evans et al., 2020; Forde et al., 2020; Hadfield & Wittkowski, 2017; Hewitt et al., 2009; A. Jones, 2019; Megnin-Viggars et al., 2015; Newman et al., 2019; Tobin et al., 2018; Watson et al., 2019  SH | 17 | *Young*  Evans | *Boyd*  *Feinberg*  *Shakespeare*  Forde  Hadfield 17  Tobin  Watson | Bina 20  Jones 19  Megnin-Viggars  Newman | Brealey  Dennis | **Moderate confidence-** the majority of studies fall under this category |
| **1.6 Logistics of accessing perinatal mental health care** | | | | | | | | |
| 1.6.1 Childcare | Lack of childcare as a barrier to PMH Care | *Boyd et al., 2011; Cox et al., 2017; Doering et al., 2017; Friedman et al., 2010*  Bina, 2020; Button et al., 2017; Dennis & Chung-Lee, 2006; Hansotte et al., 2017; Morrell et al., 2016; Newman et al., 2019; Sambrook Smith et al., 2019; Scope et al., 2017; Watson et al., 2019 | 14 | *Doering*  Morrell | *Boyd*  *Cox*  Sambrook-Smith  Watson | *Friedman*  Bina 20  Button  Hansotte  Newman  Scope | Dennis | **Low confidence -**  most studies have moderate methodological concerns |
| 1.6.2 Timing of care | Timing of appointments and services offered | *Atif et al., 2019; Friedman et al., 2010*  Bina, 2020; Dennis & Chung-Lee, 2006; Newman et al., 2019; Scope et al., 2017; Watson et al., 2019 | 7 |  | Watson | *Atif 19*  *Friedman*  Bina 20  Newman  Scope | Dennis | **Low confidence -**  most studies have moderate methodological concerns |
| 1.6.3 Location/ travel | Location of services or travel costs to get to services | *Cox et al., 2017; Doering et al., 2017; Eappen et al., 2018; Friedman et al., 2010; Masood et al., 2015; Nakku et al., 2016*  Bina, 2020; Hansotte et al., 2017; Mollard et al., 2016; Morrell et al., 2016; Newman et al., 2019; Tobin et al., 2018; Watson et al., 2019 | 13 | *Doering*  Morrell | *Cox*  *Eappen*  *Masood*  *Nakku*  Tobin  Watson | *Friedman*  Bina 20  Hansotte  Mollard  Newman |  | **Moderate confidence -**  more studies rated as high or moderate confidence. |
| **1.7 Social and family life** | | | | | | | | |
| 1.7.1 Social isolation or support | Woman’s experiences of social support or social isolation | Giscombe et al., 2020; Hansotte et al., 2017; A. Jones, 2019; C. Jones et al., 2014; Kassam, 2019; Lucas et al., 2019; Tobin et al., 2018; Viveiros & Darling, 2018; Watson et al., 2019 | 9 | Lucas | Kassam  Watson | Hanotte  Jones 19  Viveiros | Giscombe  Jones 14 | **Low confidence -** most studies have moderate methodological concerns |
| 1.7.2 Family and friends’ beliefs | Woman’s family and friends’ beliefs about mental illness | *Atif et al., 2016, 2019; Boyd et al., 2011; Doering et al., 2017; Ganann et al., 2019; Higgins et al., 2018; Masood et al., 2015; Nakku et al., 2016; Nithianandan et al., 2016; Noonan et al., 2018; O’Mahen & Flynn, 2008; Pineros-Leano et al., 2015; Vik et al., 2009; Williams et al., 2016; Young et al., 2019*  Bina, 2020; Button et al., 2017; Dennis & Chung-Lee, 2006; Forde et al., 2020; Hadfield & Wittkowski, 2017; Holopainen & Hakulinen, 2019; A. Jones, 2019; Lucas et al., 2019; Nilaweera et al., 2014; Sambrook Smith et al., 2019; Schmied et al., 2017; Sorsa et al., 2021; Viveiros & Darling, 2018; Watson et al., 2019 | 30 | *Doering*  *Nithiananden*  *O’Mahen*  *Pineros-Leano*  *Young*  Holopainen  Lucas | *Atif 16*  *Boyd*  *Ganann*  *Higgins*  *Masood*  *Nakku*  *Noonan*  *Williams*  Forde  Hadfield 17  Sambrook-Smith  Watson | *Atif 19*  *Vik*  Bina 20  Button  Jones 19  Nilaweera  Schmied  Sorsa  Viveiros | Dennis | **Moderate confidence -** more studies rated as high or moderate confidence. |
| 1.7.3 Additional personal difficulties | Personal difficulties outside of PMH such as unemployment | *Atif et al., 2016; Boyd et al., 2011; Kerker et al., 2018; Munodawafa et al., 2017; Rowan et al., 2010; Williams et al., 2016*  Hansotte et al., 2017 | 7 | *Munodawafa* | *Atif 16*  *Boyd*  *Williams* | *Kerker*  *Rowan*  Hansotte |  | **Moderate confidence -** more studies rated as high or moderate confidence. |
| **1.8 Sociodemographic factors** | | | | | | | | |
| 1.8.1 Ethnicity | Woman’s ethnicity | Bina, 2020; Dennis & Chung-Lee, 2006; Hansotte et al., 2017; Watson et al., 2019 | 4 |  | Watson | Bina 20  Hansotte | Dennis | **Low confidence -** most studies have moderate methodological concerns |
| 1.8.2 Age | Woman’s age | Bina, 2020; Hansotte et al., 2017 | 2 |  |  | Bina 20  Hansotte |  | **Low confidence –** all studies have moderate methodological concerns |
| **1.9 Mental health factors** | | | | | | | | |
| 1.9.1 Previous experiences of mental health care | Previous experiences of mental health care | *O’Mahen & Flynn, 2008*  Button et al., 2017; Evans et al., 2020; Hadfield & Wittkowski, 2017; Hansotte et al., 2017; Watson et al., 2019 | 6 | *O’Mahen*  Evans | Hadfield 17  Watson | Button  Hansotte |  | **Moderate confidence -** more studies rated as high or moderate confidence. |
| 1.9.2 Previous diagnoses or symptoms | Previous experiences of mental health symptoms or diagnoses | Bina, 2020; Sorsa et al., 2021 | 2 |  |  | Bina 20  Sorsa |  | **Low confidence -** all studies have moderate methodological concerns |
| 1.9.3 Current diagnoses or symptoms | Current experiences of mental health symptoms or diagnoses | *Chartier et al., 2015; Friedman et al., 2010; Hadfield et al., 2019; Young et al., 2019*  Sorsa et al., 2021; Viveiros & Darling, 2018 | 6 | *Young* | *Chartier* | *Friedman*  *Hadfield 19*  Sorsa  Viveiros |  | **Low confidence -** most studies have moderate methodological concerns |
| **2. HCPs** | | | | | | | | |
| **2.1 HCPs knowledge about PMH** | | | | | | | | |
| 2.1.1 HCPs knowledge about PMI | HCPs knowledge about PMH actual & perceived by women | *Beeber et al., 2009; Byatt et al., 2013; Ganann et al., 2019; Higgins et al., 2018; Judd et al., 2011; McCauley et al., 2019; McKenzie-McHarg et al., 2014; Noonan et al., 2018; Reed et al., 2014; Rowan et al., 2010*  Bina, 2020; Dennis & Chung-Lee, 2006; Megnin-Viggars et al., 2015; Morrell et al., 2016; Slade et al., 2020; Viveiros & Darling, 2018  SH | 17 | *McCauley*  Morrell | *Beeber*  *Ganann*  *Higgins*  *Judd*  *Noonan*  *Reed*  Slade | *Byatt*  *Rowan*  Bina 20  Megnin-Viggars  Viveiros | *McKenzie-McHarg*  Dennis | **Moderate confidence -** more studies rated as high or moderate confidence. |
| 2.1.2 HCP’s knowledge about services/referral pathways | HCPs knowledge about PMH services and referral pathways actual & perceived by women | *Ganann et al., 2019; Higgins et al., 2018; Rowan et al., 2010*  Dennis & Chung-Lee, 2006; Hansotte et al., 2017; Sambrook Smith et al., 2019; Slade et al., 2020; Viveiros & Darling, 2018 | 8 |  | *Gannan*  *Higgins*  Sambrook-Smith  Slade | *Rowan*  Hansotte  Viveiros | Dennis | **Moderate confidence -** even split, round up more SRs |
| 2.1.3 HCPs confidence | HCP's confidence in addressing PMH | *Atif et al., 2019; Bina et al., 2018; Cox et al., 2017; Fernandez y Garcia et al., 2011; Higgins et al., 2018; Munodawafa et al., 2017; Nithianandan et al., 2016; Ormsby et al., 2018; Reed et al., 2014*  SH | 9 | *Munodawafa*  *Nithiananden*  *Ormsby* | *Bina 18*  *Cox*  *Fernandez y Garcia*  *Higgins*  *Reed* | *Atif 19* |  | **Moderate confidence -** most studies rated as high or moderate confidence. |
| **2.2 Getting it right the first time** | | | | | | | | |
| 2.2.1 Being dismissive or normalising symptoms | HCPs dismissing or normalising symptoms | *Ganann et al., 2019*  Button et al., 2017; Dennis & Chung-Lee, 2006; Forde et al., 2020; Hadfield & Wittkowski, 2017; Hansotte et al., 2017; Megnin-Viggars et al., 2015; Newman et al., 2019; Sorsa et al., 2021; Watson et al., 2019  SH | 11 |  | *Ganann*  Forde  Hadfield 17  Watson | Button  Megnin-Viggars  Newman  Sorsa | Dennis | **Low confidence -** most studies have moderate methodological concerns |
| 2.2.2 Not recognising help seeking or PMI | HCPs not recognising help seeking or PMI | Bina, 2020; Button et al., 2017; Megnin-Viggars et al., 2015; Tobin et al., 2018; Watson et al., 2019 | 5 |  | Tobin  Watson | Bina 20  Button  Megnin-Viggars |  | **Low confidence -** most studies have moderate methodological concerns |
| 2.2.3 Focussing on infant | HCPs focussing mainly on the infant | Button et al., 2017; Megnin-Viggars et al., 2015 | 2 |  |  | Button  Megnin-Viggars |  | **Low confidence -** all studies have moderate methodological concerns |
| 2.2.4 Making time | A HCP who makes time to address PMH concerns | *Feinberg et al., 2006; Myors et al., 2015; Noonan et al., 2018*  Bina, 2020; Button et al., 2017; Dennis & Chung-Lee, 2006; Hewitt et al., 2009; Megnin-Viggars et al., 2015; Slade et al., 2020; Viveiros & Darling, 2018; Watson et al., 2019 | 11 | Hewitt | *Feinberg*  *Myors*  *Noonan*  Slade  Watson | Bina 20  Button  Megnin-Viggars  Viveiros | Dennis | **Moderate confidence -** most studies rated as high or moderate confidence. |
| 2.2.5 Assessment specific behaviours | HCP's assessment specific behaviours, such as asking about PMH, carrying out in a tick box way, or in a personalised way | *Doering et al., 2017; Fernandez y Garcia et al., 2011; Nithianandan et al., 2016; Segre et al., 2014; Vik et al., 2009; Williams et al., 2016*  Brealey et al., 2010; Sambrook Smith et al., 2019; Schmied et al., 2017; Slade et al., 2020; Viveiros & Darling, 2018; Watson et al., 2019 | 12 | *Doering*  *Nithiananden* | *Fernandez y Garcia*  *Segre*  *Williams*  Sambrook-Smith  Slade  Watson | *Vik*  Schmied  Viveiros | Brealey | **Moderate confidence -** most studies rated as high or moderate confidence. |
| **2.3 HCPs’ attributes** | | | | | | | | |
| 2.3.1 Similar demographic characteristics | HCP having similar demographics to women | *Leger et al., 2015; Masood et al., 2015; Nithianandan et al., 2016; Shorey & Ng, 2019*  Dennis & Chung-Lee, 2006; Watson et al., 2019 | 6 | *Nithiananden* | *Leger*  *Masood*  *Shorey*  Watson |  | Dennis | **Moderate confidence -** most studies rated as high or moderate confidence. |
| 2.3.2 Culturally sensitive | HCP being sensitive to women from all cultures | Kassam, 2019; Nilaweera et al., 2014; Viveiros & Darling, 2018; Watson et al., 2019*;* | 4 |  | Kassam  Watson | Nilaweera  Viveiros |  | **Moderate confidence -**  even split, round up because all SRs |
| 2.3.3 Valued characteristics | HCPs possessing valued characteristics Trustworthy, empathetic, kind, caring with a genuinine interest, and going above and beyond | *Atif et al., 2016, 2019; Boyd et al., 2011; Doering et al., 2017; Kerker et al., 2018; Kim et al., 2009; Munodawafa et al., 2017; Myors et al., 2015; Pugh et al., 2015; Shorey & Ng, 2019*  Brealey et al., 2010; Button et al., 2017; Dennis & Chung-Lee, 2006; Forde et al., 2020; Hadfield & Wittkowski, 2017; Hewitt et al., 2009; Jones, 2019; Megnin-Viggars et al., 2015; Morrell et al., 2016; Newman et al., 2019; Schmied et al., 2017; Slade et al., 2020; Staneva et al., 2015; Watson et al., 2019  SH | 25 | *Doering*  *Munodawafa*  *Pugh*  Hewitt  Morrell | *Atif 16*  *Boyd*  *Myors*  *Shorey*  Forde  Hadfield 17  Slade  Staneva  Watson | *Kerker*  *Kim*  Button  Jones 19  Megnin-Viggars  Newman  Schmied | Brealey  Dennis | **Moderate confidence -** most studies rated as high or moderate confidence. |
| **3. Interpersonal** | | | | | | | | |
| 3.1 Trusting relationship and rapport | The development of a trusting relationship and rapport between HCP and women | *Atif et al., 2016; Doering et al., 2017; Feinberg et al., 2006; Ganann et al., 2019; Hadfield et al., 2019; Higgins et al., 2018; Kerker et al., 2018; Leger et al., 2015; Noonan et al., 2018; Shakespeare et al., 2003; Shorey & Ng, 2019; Willey et al., 2018; Williams et al., 2016; Young et al., 2019*  Bina, 2020; Brealey et al., 2010; Dennis & Chung-Lee, 2006; Hadfield & Wittkowski, 2017; Hewitt et al., 2009; Megnin-Viggars et al., 2015; Morrell et al., 2016; Scope et al., 2017; Tobin et al., 2018 | 23 | *Doering*  *Young*  Hewitt  Morrell | *Atif 16*  *Feinberg*  *Ganann*  *Higgins*  *Leger*  *Noonan*  *Shakespeare*  *Shorey*  *Willey*  *Williams*  Hadfield 17  Tobin | *Hadfield 19*  *Kerker*  Bina 20  Megnin-Viggars  Scope | Brealey  Dennis | **Moderate confidence -** most studies rated as high or moderate confidence. |
| 3.2 Language barriers | Difficulties in communicating due to language barriers | *Beeber et al., 2009; Doering et al., 2017; Ganann et al., 2019; Masood et al., 2015; Munodawafa et al., 2017; Nithianandan et al., 2016; Pineros-Leano et al., 2015; Segre et al., 2014; Willey et al., 2018; Williams et al., 2016*  Dennis & Chung-Lee, 2006; Hansotte et al., 2017; Megnin-Viggars et al., 2015; Sambrook Smith et al., 2019; Schmied et al., 2017; Watson et al., 2019  SH | 16 | *Doering*  *Munodawafa*  *Nithiananden*  *Pineros-Leano* | *Beeber*  *Ganann*  *Masood*  *Segre*  *Willey*  *Williams*  Sambrook-Smith  Watson | Hansotte  Megnin-Viggars  Schmied | Dennis | **Moderate confidence -** most studies rated as high or moderate confidence. |
| 3.3 Shared decision making | Shared decision making between HCP and woman | Hadfield & Wittkowski, 2017; Megnin-Viggars et al., 2015; Randall & Briscoe, 2018; Scope et al., 2017 | 4 | Hewitt | Hadfield 17 | Megnin-Viggars  Randall  Scope |  | **Low confidence -** most studies have moderate methodological concerns |
| 3.4 Open and honest communication | Open and honest communication between HCP and woman | *Doering et al., 2017; Shakespeare et al., 2003; Vik et al., 2009; Willey et al., 2018*  Brealey et al., 2010; Hadfield & Wittkowski, 2017; Hewitt et al., 2009; Schmied et al., 2017; Watson et al., 2019  SH | 9 | *Doering*  Hewitt | *Shakespeare*  *Willey*  Hadfield 17  Watson | *Vik*  Schmied | Brealey | **Moderate confidence -** most studies rated as high or moderate confidence. |
| **4. Organisational** | | | | | | | | |
| **4.1 Overall organisational aspects** | | | | | | | | |
| 4.1.1 Co location and buildings | Location of the service including co-location of different services within the same building | *Boyd et al., 2011; Cox et al., 2017; Judd et al., 2011; Munodawafa et al., 2017; Ormsby et al., 2018; Young et al., 2019;*  Bina, 2020  SH | 7 | *Munodawafa*  *Ormsby*  *Young* | *Boyd*  *Cox* | *Judd*  Bina 20 |  | **Moderate confidence -** most studies rated as high or moderate confidence. |
| 4.1.2 Service integration and collaborative working | Collaborative working across services  SH | *Atif et al., 2016; Bina et al., 2018; Boyd et al., 2011; Byatt et al., 2013; Feinberg et al., 2006; Ganann et al., 2019; Hadfield et al., 2019; Judd et al., 2011; Lind et al., 2017; Lomonaco-Haycraft et al., 2018; Myors et al., 2015; Nithianandan et al., 2016; Noonan et al., 2018; Rowan et al., 2010*  Newman et al., 2019; Sambrook Smith et al., 2019; Watson et al., 2019 | 17 | *Nithiananden* | *Atif 16*  *Bina 18*  *Boyd*  *Feinberg*  *Ganann*  *Myors*  *Noonan*  Sambrook-Smith  Watson | *Byatt*  *Hadfield*  *Judd*  *Lind*  *Lomonaco-Haycraft*  *Rowan*  Newman |  | **Moderate confidence -** most studies rated as high or moderate confidence. |
| 4.1.3 Collaboration within services | Collaborative working within services  SH | *Ammerman et al., 2014; Cox et al., 2017; Eappen et al., 2018; Higgins et al., 2018; Judd et al., 2011; Kerker et al., 2018; Lind et al., 2017; McKenzie-McHarg et al., 2014; Munodawafa et al., 2017; Nithianandan et al., 2016; Ormsby et al., 2018; Segre et al., 2014; Willey et al., 2018*  Sambrook Smith et al., 2019 | 14 | *Munodawafa*  *Nithiananden*  *Ormsby* | *Cox*  *Eappen*  *Higgins*  *Segre*  *Willey*  Sambrook-Smith | *Ammerman*  *Judd*  *Kerker*  *Lind* | *McKenzie-McHarg* | **Moderate confidence -** most studies rated as high or moderate confidence. |
| 4.1.4 Adequate workforce provision/ HCPs workload | Ensuring and adequate workforce provision so PMH can be addressed | *Ammerman et al., 2014; Bina et al., 2018; Drozd et al., 2018; Feinberg et al., 2006; Ganann et al., 2019; Higgins et al., 2018; Kerker et al., 2018; Kim et al., 2009; McCauley et al., 2019; Nakku et al., 2016; Nithianandan et al., 2016; Noonan et al., 2018; Rowan et al., 2010; Vik et al., 2009; Willey et al., 2018*  Bina, 2020; Viveiros & Darling, 2018  SH | 17 | *McCauley*  *Nithiananden* | *Bina 18*  *Feinberg*  *Ganann*  *Higgins*  *Nakku*  *Noonan*  *Willey* | *Ammerman*  *Drozd*  *Kerker*  *Kim*  *Rowan*  *Vik* | Bina 20  Viveiros | **Moderate confidence -** most studies rated as high or moderate confidence. |
| 4.1.5 Clear assessment and referral process | Clear assessment and referral processes within the organisation | *Cox et al., 2017; Feinberg et al., 2006; Ganann et al., 2019; Judd et al., 2011; Kerker et al., 2018; Kim et al., 2009; Nithianandan et al., 2016; Rowan et al., 2010; Segre et al., 2014; Williams et al., 2016*  Bina, 2020  SH | 11 | *Nithiananden* | *Cox*  *Feinberg*  *Ganann*  *Segre*  *Williams* | *Kerker*  *Kim*  *Rowan*  Bina 20 |  | **Moderate confidence -** most studies rated as high or moderate confidence. |
| 4.1.6 Provision of supervision | Supervision for HCPs | *Atif et al., 2019; Munodawafa et al., 2017; Vik et al., 2009* | 3 | *Munodawafa* |  | *Atif 19*  *Vik* |  | **Low confidence -** most studies have moderate methodological concerns |
| 4.1.7 Training | Provision of training for all HCPs working with perinatal women | *Ammerman et al., 2014; Atif et al., 2016, 2019; Beeber et al., 2009; Bina et al., 2018; Boyd et al., 2011; Chartier et al., 2015; Doering et al., 2017; Drozd et al., 2018; Feinberg et al., 2006; Ganann et al., 2019; Judd et al., 2011; Kerker et al., 2018; Kim et al., 2009; Leger et al., 2015; Lind et al., 2017; McKenzie-McHarg et al., 2014; Munodawafa et al., 2017; Nakku et al., 2016; Nithianandan et al., 2016; Noonan et al., 2018; Reed et al., 2014; Rowan et al., 2010; Shorey & Ng, 2019; Willey et al., 2018; Williams et al., 2016*  Bina, 2020; Brealey et al., 2010  SH | 28 | *Doering*  *Munodawafa*  *Nithiananden* | *Atif 16*  *Beeber*  *Bina 18*  *Boyd*  *Chartier*  *Ganann*  *Leger*  *Noonan*  *Reed*  *Shorey*  *Willey*  *Williams* | *Ammerman*  *Atif 19*  *Drozd*  *Judd*  *Kerker*  *Kim*  *Lind*  *Rowan*  Bina 20 | *McKenzie-McHarg*  Brealey | **Moderate confidence -** most studies rated as high or moderate confidence. |
| 4.1.9 Organisational goals/guidelines | Clear organisational goals and guidelines | *Ammerman et al., 2014; Willey et al., 2018* | 2 |  | *Willey* | *Ammerman* |  | **Low confidence –** even split, round down because not SRs |
| **4.2 Characteristics of PMH Care** | | | | | | | | |
| **4.2.1 Across the care pathway** | | | | | | | | |
| 4.2.1.1 Continuity of carer | Care that provides the same HCP along the care pathway | *Chartier et al., 2015; Higgins et al., 2018; Nithianandan et al., 2016; O’Mahen & Flynn, 2008; Rowan et al., 2010; Willey et al., 2018*  Brealey et al., 2010; Button et al., 2017; Dennis & Chung-Lee, 2006; Hadfield & Wittkowski, 2017; Megnin-Viggars et al., 2015; Sambrook Smith et al., 2019; Slade et al., 2020; Tobin et al., 2018; Viveiros & Darling, 2018; Watson et al., 2019  SH | 17 | *Nithiananden*  *O’Mahen* | *Chartier*  *Higgins*  *Willey*  Hadfield 17  Sambrook-Smith  Slade  Tobin  Watson | *Rowan*  Button  Megnin-Viggars  Viveiros | Brealey  Dennis | **Moderate confidence -** most studies rated as high or moderate confidence. |
| 4.2.1.2 Culturally sensitive care | Care that is culturally sensitive to women's needs | *Ganann et al., 2019; Nithianandan et al., 2016; Noonan et al., 2018; Shorey & Ng, 2019*  Bina, 2020; Brealey et al., 2010; Button et al., 2017; Dennis & Chung-Lee, 2006; Giscombe et al., 2020; Hadfield & Wittkowski, 2017; Hansotte et al., 2017; Hewitt et al., 2009; Jones, 2019; Kassam, 2019; Sambrook Smith et al., 2019; Schmied et al., 2017; Tobin et al., 2018; Viveiros & Darling, 2018; Watson et al., 2019 | 19 | *Nithiananden*  Hewitt | *Ganann*  *Noonan*  *Shorey*  Hadfield  Kassam  Sambrook-Smith  Tobin  Watson | Bina 20  Button  Hansotte  Jones 19  Schmied  Viveiros | Brealey  Dennis  Giscombe | **Moderate confidence -** most studies rated as high or moderate confidence. |
| 4.2.1.3 Privacy & confidentiality | Care that is private and maintains women's confidentiality | *Atif et al., 2019; Feinberg et al., 2006; Higgins et al., 2018; Jallo et al., 2015; Nithianandan et al., 2016; O’Mahen & Flynn, 2008*  Giscombe et al., 2020 | 7 | *Nithiananden*  *O’Mahen* | *Atif 16*  *Feinberg*  *Higgins*  *Jallo* |  | Giscombe | **Moderate confidence -** most studies rated as high or moderate confidence. |
| 4.2.1.4 Dedicated person/ PMH Champion | Care that has a dedicated person or PMH champion | *Chartier et al., 2015; Ganann et al., 2019; Kim et al., 2009; Lomonaco-Haycraft et al., 2018; Nithianandan et al., 2016; Rowan et al., 2010; Willey et al., 2018* Bina, 2020; Megnin-Viggars et al., 2015 | 9 |  | *Chartier*  *Ganann*  *Willey* | *Kim*  *Lomonaco-Haycraft*  *Rowan*  Bina  Megnin-Viggars |  | **Low confidence -** most studies have moderate methodological concerns |
| 4.2.1.5 Logistical support | Logistical support for women including easily accessible location, childcare, travel costs | *Ganann et al., 2019; Hadfield & Wittkowski, 2017; Leger et al., 2015; Masood et al., 2015; Nakku et al., 2016; Nithianandan et al., 2016; Ormsby et al., 2018*  Button et al., 2017; Jones, 2019; Mollard et al., 2016; Newman et al., 2019; Scope et al., 2017; Watson et al., 2019 | 13 | *Nithiananden*  *Ormsby* | *Ganann*  *Leger*  *Masood*  *Nakku*  Watson | *Hadfield 19*  Button  Jones 19  Mollard  Newman  Scope |  | **Moderate confidence -** most studies rated as high or moderate confidence. |
| 4.2.1.6 Home delivery | Care that is delivered at home | *Ammerman et al., 2014; Beeber et al., 2009; Judd et al., 2011; Leger et al., 2015; Munodawafa et al., 2017; Myors et al., 2015*  Brealey et al., 2010; Hadfield & Wittkowski, 2017; Hansotte et al., 2017; Jones, 2019 | 10 | *Munodawafa* | *Beeber*  *Leger*  *Myors*  Hadfield 17 | *Ammerman*  *Judd*  Hansotte  Jones 19 | Brealey | **Moderate confidence –** even split, rounded up because SRs* |
| 4.2.1.7 Hospital delivery | Care that is delivered in hopsital/medical setting | *Atif et al., 2019; Boyd et al., 2011; Kerker et al., 2018; Shakespeare et al., 2003*  Dennis & Chung-Lee, 2006 | 5 |  | *Boyd*  *Shakespeare* | *Atif 19*  *Kerker* | Dennis | **Low confidence -** most studies have moderate methodological concerns |
| 4.2.1.8 Provision of information | Whether care provides information | Dennis & Chung-Lee, 2006; Hadfield & Wittkowski, 2017; Jones, 2019; Megnin-Viggars et al., 2015; Morrell et al., 2016; Randall & Briscoe, 2018; Schmied et al., 2017 | 7 | Morrell | Hadfield 17 | Jones 19  Megnin-Viggars  Randall  Schmied | Dennis | **Low confidence -** most studies have moderate methodological concerns |
| 4.2.1.9 Technology | The use of technology in care | *Doering et al., 2017; Feinberg et al., 2006; Fernandez y Garcia et al., 2011; Jallo et al., 2015; Kim et al., 2009; Lind et al., 2017; Noonan et al., 2018; Pineros-Leano et al., 2015; Shorey & Ng, 2019; Willey et al., 2018; Williams et al., 2016*  SH | 11 | *Doering*  *Pineros-Leano* | *Feinberg*  *Fernandez y Garcia*  *Jallo*  *Noonan*  *Shorey*  *Willey*  *Williams* | *Kim*  *Lind* |  | **Moderate confidence -** most studies rated as high or moderate confidence. |
| 4.2.1.10 Service inclusion criteria | Inclusion criteria of services | *Boyd et al., 2011; Ganann et al., 2019*  Viveiros & Darling, 2018 | 3 |  | *Boyd*  *Ganann* | Viveiros |  | **Moderate confidence -** most studies rated as high or moderate confidence. |
| **4.2.2 Assessment specific characteristics** | | | | | | | | |
| 4.2.2.1 Wording of assessment tools | How assessment tools are worded | *Doering et al., 2017; Segre et al., 2014; Williams et al., 2016*  Brealey et al., 2010; Button et al., 2017; Hewitt et al., 2009 | 6 | *Doering*  Hewitt | *Segre*  *Williams* | Button | Brealey | **Moderate confidence -** most studies rated as high or moderate confidence. |
| 4.2.2.2 Acceptability of assessment/ screening | Whether assessment tools and assessment/screening in general are acceptable to women and HCPs | *Boyd et al., 2011; Doering et al., 2017; Feinberg et al., 2006; Ganann et al., 2019; Kim et al., 2009; Nithianandan et al., 2016; Segre et al., 2014; Shakespeare et al., 2003; Vik et al., 2009; Willey et al., 2018*  Brealey et al., 2010; Evans et al., 2020; Hewitt et al., 2009; Megnin-Viggars et al., 2015; Mollard et al., 2016; Sambrook Smith et al., 2019; Viveiros & Darling, 2018 | 17 | *Doering*  *Nithiananden*  Evans  Hewitt | *Boyd*  *Feinberg*  *Ganann*  *Segre*  *Shakespeare*  *Willey*  Sambrook-Smith | *Kim*  *Vik*  Megnin-Viggars  Mollard  Viveiros | Brealey | **Moderate confidence -** most studies rated as high or moderate confidence. |
| **4.2.3 Intervention characteristics** | | | | | | | | |
| 4.2.3.1 Opportunity to talk | Interventions that provide an opportunity to talk | Dennis & Chung-Lee, 2006; Evans et al., 2020; Hadfield & Wittkowski, 2017; Jones et al., 2014; Kassam, 2019; Morrell et al., 2016; Praetorius et al., 2020 | 7 | Evans  Morrell | Hadfield 17  Kassam | Praetorius | Dennis  Jones 14 | **Low confidence -** most studies have moderate methodological concerns |
| 4.2.3.2 Individualised and person centred | Indiviudalised and person centred interventions/care | *Chartier et al., 2015; Doering et al., 2017; Ganann et al., 2019; Masood et al., 2015; McKenzie-McHarg et al., 2014; Noonan et al., 2018; O’Mahen & Flynn, 2008; Pugh et al., 2015; Segre et al., 2014; Shorey & Ng, 2019*  Evans et al., 2020; Hadfield & Wittkowski, 2017; Megnin-Viggars et al., 2015; Morrell et al., 2016; Schmied et al., 2017; Scope et al., 2017; Slade et al., 2020; Watson et al., 2019  SH | 19 | *Doering*  *O’Mahen*  *Pugh*  Evans  Morrell | *Chartier*  *Ganann*  *Masood*  *Noonan*  *Segre*  *Shorey*  Hadfield 17  Slade  Watson | Megnin-Viggars  Schmied  Scope | *McKenzie-McHarg* | **Moderate confidence -** most studies rated as high or moderate confidence. |
| 4.2.3.3 Appropriateness | Appropriateness of intervention being offered, from women and HCPs point of view | *Atif et al., 2019; Bina et al., 2018; Chartier et al., 2015; Drozd et al., 2018; Leger et al., 2015; McKenzie-McHarg et al., 2014; Munodawafa et al., 2017; Noonan et al., 2018; Ormsby et al., 2018; Pugh et al., 2015; Reed et al., 2014; Shorey & Ng, 2019*  Evans et al., 2020; Megnin-Viggars et al., 2015; Scope et al., 2017 | 15 | *Munodawafa*  *Ormsby*  *Pugh*  Evans | *Bina 18*  *Chartier*  *Leger*  *Noonan*  *Reed*  *Shorey* | *Atif 19*  *Drozd*  Megnin-Viggars  Scope | *McKenzie-McHarg* | **Moderate confidence -** most studies rated as high or moderate confidence. |
| 4.2.3.4 Flexible | Flexibility of intervention | *Atif et al., 2019; Bina et al., 2018; Ganann et al., 2019; Hadfield et al., 2019; Judd et al., 2011; Munodawafa et al., 2017; O’Mahen & Flynn, 2008; Pugh et al., 2015; Shorey & Ng, 2019*  Sorsa et al., 2021; Watson et al., 2019 | 11 | *Munodawafa*  *O’Mahen*  *Pugh* | *Bina 18*  *Ganann*  *Shorey*  Watson | *Atif 19*  *Hadfield 19*  *Judd*  Sorsa |  | **Moderate confidence -** most studies rated as high or moderate confidence. |
| 4.2.3.5 Group support | Group/peer support as an intervention | *Hadfield et al., 2019; Masood et al., 2015; Nakku et al., 2016*  Dennis & Chung-Lee, 2006; Evans et al., 2020; Hadfield & Wittkowski, 2017; Holopainen & Hakulinen, 2019; Jones et al., 2014; Megnin-Viggars et al., 2015; Schmied et al., 2017; Scope et al., 2017; Slade et al., 2020; Tobin et al., 2018; Watson et al., 2019  SH | 14 | Evans  Holopainen | *Masood*  *Nakku*  Hadfield 17  Slade  Tobin  Watson | *Hadfield 19*  Megnin-Viggars  Schmied  Scope | Dennis  Jones 14 | **Moderate confidence -** most studies rated as high or moderate confidence. |
| 4.2.3.7 Face to face delivery | Face to face delivery of intervention | *O’Mahen & Flynn, 2008; Pugh et al., 2015; Shorey & Ng, 2019*  Schmied et al., 2017  SH | 4 | *O’Mahen*  *Pugh* | *Shorey* | Schmied |  | **Moderate confidence -** most studies rated as high or moderate confidence. |
| **5. Commissioners** | | | | | | | | |
| 5.1 Referral pathways | Clear referral pathways | *Ammerman et al., 2014; Boyd et al., 2011; Higgins et al., 2018; Nithianandan et al., 2016; Rowan et al., 2010; Willey et al., 2018*  SH | 6 | *Nithiananden* | *Boyd*  *Higgins*  *Willey* | *Ammerman*  *Rowan* |  | **Moderate confidence -** most studies rated as high or moderate confidence. |
| 5.2 Lack of appropriate or timely services | Lack of appropriate and timely services to refer women onto | *Boyd et al., 2011; Doering et al., 2017; Ganann et al., 2019; Higgins et al., 2018; Kerker et al., 2018; Leger et al., 2015; Lomonaco-Haycraft et al., 2018; Munodawafa et al., 2017; Myors et al., 2015; Nakku et al., 2016; Noonan et al., 2018; Rowan et al., 2010; Williams et al., 2016*  Bina, 2020; Button et al., 2017; Jones, 2019; Jones et al., 2014; Megnin-Viggars et al., 2015; Newman et al., 2019; Sambrook Smith et al., 2019; Tobin et al., 2018; Viveiros & Darling, 2018  SH | 22 | *Doering*  *Munodawafa* | *Boyd*  *Ganann*  *Higgins*  *Leger*  *Myors*  *Nakku*  *Noonan*  *Williams*  Sambrook-Smith  Tobin | *Kerker*  *Lomonaco-Haycraft*  *Rowan*  Bina 20  Button  Jones 19  Megnin-Viggars  Newman  Viveiros | Jones 14 | **Moderate confidence -** most studies rated as high or moderate confidence. |
| 5.3 Financial complexities | Financial complexities including funding, and sourcing money and resources for services and financial reimbursement | *Cox et al., 2017; Feinberg et al., 2006; Friedman et al., 2010; Ganann et al., 2019; Kim et al., 2009; Lomonaco-Haycraft et al., 2018; Ormsby et al., 2018; Rowan et al., 2010*  SH | 8 | *Ormsby* | *Cox*  *Feinberg*  *Ganann* | *Friedman*  *Kim*  *Lomonaco-Haycraft*  *Rowan* |  | **Moderate confidence –** Weighting of high confidence for one paper means we round up |
| **6. Political** | | | | | | | | |
| 6.1 Immigration status | How the immigration status of women may impact their PMH care journey | *Cox et al., 2017; Ganann et al., 2019*  Bina, 2020; Giscombe et al., 2020; Hansotte et al., 2017; Kassam, 2019; Schmied et al., 2017; Tobin et al., 2018; Watson et al., 2019  SH | 9 |  | *Cox*  *Ganann*  Hansotte 17  Kassam  Tobin  Watson | Bina 20  Schmied | Giscombe | **Moderate confidence -** most studies rated as high or moderate confidence. |
| 5.2 Economic status & healthcare costs | How the cost of healthcare, and women's economic status may impact their PMH care journey | *Atif et al., 2016; Boyd et al., 2011; Cox et al., 2017; Doering et al., 2017; Ganann et al., 2019; Lomonaco-Haycraft et al., 2018; Munodawafa et al., 2017; Nakku et al., 2016; Ormsby et al., 2018*  Bina, 2020; Hansotte et al., 2017; Jones, 2019; Kassam, 2019; Lucas et al., 2019; Tobin et al., 2018; Viveiros & Darling, 2018  SH | 16 | *Doering*  *Ormsby*  Lucas | *Atif 16*  *Boyd*  *Cox*  *Ganann*  *Nakku*  Kassam  Tobin | *Lomonaca-Haycraft*  Bina 20  Hansotte  Jones 19  Viveiros |  | **Moderate confidence -** most studies rated as high or moderate confidence. |
| **7. Societal** | | | | | | | | |
| 7.1 Stigma | Stigma related to mental illness | *Atif et al., 2016, 2019; Boyd et al., 2011; Chartier et al., 2015; Cox et al., 2017; Feinberg et al., 2006; Higgins et al., 2018; Kerker et al., 2018; McCauley et al., 2019; McKenzie-McHarg et al., 2014; Munodawafa et al., 2017; Myors et al., 2015; Nakku et al., 2016; Nithianandan et al., 2016; Noonan et al., 2018; O’Mahen & Flynn, 2008; Shakespeare et al., 2003; Vik et al., 2009; Williams et al., 2016; Young et al., 2019*  Bina et al., 2018; Button et al., 2017; Dennis & Chung-Lee, 2006; Giscombe et al., 2020; Hadfield & Wittkowski, 2017; Hansotte et al., 2017; Hewitt et al., 2009; Holopainen & Hakulinen, 2019; A. Jones, 2019; Kassam, 2019; Lucas et al., 2019; Megnin-Viggars et al., 2015; Mollard et al., 2016; Morrell et al., 2016; Nilaweera et al., 2014; Sambrook Smith et al., 2019; Schmied et al., 2017; Scope et al., 2017; Sorsa et al., 2021; Tobin et al., 2018; Viveiros & Darling, 2018; Watson et al., 2019  SH | 43 | *McCauley*  *Munodowafa*  *Nithiananden*  *O’Mahen*  *Young*  Holopainen  Lucas  Morrell | *Atif 16*  *Boyd*  *Chartier*  *Feinberg*  *Higgins*  *Myors*  *Nakku*  *Noonan*  *Shakespeare*  *Williams*  Hadfield 17  Kassam  Sambrook-Smith  Tobin  Watson | *Atif 19*  *Kerker*  *Vik*  Bina 20  Button  Hansotte  Jones 19  Megnin-Viggars  Mollard  Nilaweera  Schmied  Scope  Sorsa  Viveiros | *McKenzie-McHarg*  Dennis  Giscombe | **Moderate confidence -** most studies rated as high or moderate confidence. |
| 7.2 Culture | Cultural beliefs about mental illness and seeking and accessing help | *Atif et al., 2016; Bina et al., 2018; Boyd et al., 2011; Feinberg et al., 2006; Friedman et al., 2010; Ganann et al., 2019; Higgins et al., 2018; Masood et al., 2015; McCauley et al., 2019; Nakku et al., 2016; Noonan et al., 2018; Segre et al., 2014*  Brealey et al., 2010; Button et al., 2017; Dennis & Chung-Lee, 2006; Giscombe et al., 2020; Hansotte et al., 2017; Hewitt et al., 2009; Holopainen & Hakulinen, 2019; Jones, 2019; Kassam, 2019; Megnin-Viggars et al., 2015; Praetorius et al., 2020; Sambrook Smith et al., 2019; Schmied et al., 2017; Staneva et al., 2015; Tobin et al., 2018; Viveiros & Darling, 2018; Watson et al., 2019; Wittkowski et al., 2014  SH | 30 | *McCauley*  Holopainen  Hewitt | *Atif 16*  *Bina 18*  *Boyd*  *Feinberg*  *Ganann*  *Higgins*  *Masood*  *Nakku*  *Noonan*  *Segre*  Kassam  Sambrook-Smith  Staneva  Tobin  Watson | *Friedman*  Button  Hansotte  Jones 19  Megnin-Viggars  Praetorius  Schmied | Brealey  Dennis  Giscombe | **Moderate confidence -** most studies rated as high or moderate confidence. |
| 7.3 Maternal norms | Maternal norms of being a "good mother" and a "strong woman" | *Shakespeare et al., 2003; Williams et al., 2016*  Bina, 2020; Brealey et al., 2010; Button et al., 2017; Dennis & Chung-Lee, 2006; Hadfield & Wittkowski, 2017; Hansotte et al., 2017; Hewitt et al., 2009; Holopainen & Hakulinen, 2019; Johnson et al., 2020; Jones et al., 2014; Kassam, 2019; Lucas et al., 2019; Megnin-Viggars et al., 2015; Mollard et al., 2016; Newman et al., 2019; Nilaweera et al., 2014; Praetorius et al., 2020; Sambrook Smith et al., 2019; Schmied et al., 2017; Slade et al., 2020; Sorsa et al., 2021; Staneva et al., 2015; Viveiros & Darling, 2018; Watson et al., 2019  SH | 27 | Hewitt  Holopainen  Lucas | *Shakespeare*  *Williams*  Hadfield  Kassam  Sambrook-Smith  Slade  Staneva  Watson | Bina 20  Button  Hansotte  Megnin-Viggars  Mollard  Newman  Nilaweera  Praetorius  Sorsa  Viveiros | Brealey  Dennis  Jones 14 | **Low confidence -** most studies have moderate methodological concerns |

Overall, the systematic reviews received lower methodological quality scores, however when there is an even split, if there are more systematic reviews, the higher quality rating is chosen. This is because systematic reviews are seen as higher in the evidence pyramid, than primary research studies (<https://academicguides.waldenu.edu/library/healthevidence/evidencepyramid>).

# *Supplementary Materials 5:* GRADE-CERQual Coherence rating table

| **Theme** | **Definition of theme** | **Studies citing this theme** | **N** | **Methodological rating**  **Adequacy rating**  **Coherence rating**  **Relevance rating** | | | | **Overall CERQUAL Rating** |
| --- | --- | --- | --- | --- | --- | --- | --- | --- |
| **1 Women** | | | | | | | | |
| **1.1 Beliefs about health services** | | | | | | | | |
| 1.1.1 Services only offer medication | The belief that health services will only offer medication to treat PMH concerns | *Doering et al., 2017; Ganann et al., 2019; Williams et al., 2016; Young et al., 2019;*  Bina, 2020; Button et al., 2017; Dennis & Chung-Lee, 2006; Hadfield & Wittkowski, 2017; Jones, 2019; Megnin-Viggars et al., 2015; Nilaweera et al., 2014; Sorsa et al., 2021; Tobin et al., 2018 | 14 | *Doering – Quote from HV about how women don’t want to go on medication*  *Ganann – Some women avoid accessing psychiatric care if they perceive medication will be pushed*  *Williams – women say they don’t want help because they don’t want to take antidepressants*  *Young – Women didn’t want to take medications because worried it would intefer with parental duties and get into breast milk*  Bina 20 – Medication seen as likely but unwanted treatment offer  Button – Women concerned about the side effects of medication  Dennis – Women reluctant to take medication  Hadfield 17 – Women felt ashamed to take medication  Jones 19 – Fear of taking medication because of perceived side effects  Megnin-Viggars – Women felt medication was the only treatment available  Nilaweera – Women concerned antidepressants might affect the baby through the breastmilk  Sorsa – Women expressed fear of medication  Tobin – Women did not disclose because they thought the doctor was likely to write a prescription | | | | **High confidence -**  all studies are saying the same thing |
| 1.1.2 Services are stretched | The belief that PMH services are too stretched and will therefore be unable to help women | Dennis & Chung-Lee, 2006; Hadfield & Wittkowski, 2017 | 2 | Dennis –Women felt they would be burdensome if they disclosed their feelings  Hadfield 17 – Women perceived health services as under resourced. | | | | **Low confidence -**  Dennis study open to interpretation |
| 1.1.3 Services are too complicated | Services being too complex or complicated | *Ganann et al., 2019*  Tobin et al., 2018  SH | 2 | *Ganann – Women who had immigrated di not understand the healthcare system*  Tobin – Women did not understand how health systems worked  SH – It was reported that women found the services complicated. | | | | **High confidence** |
| 1.1.4 Women’s mistrust and fear of services | Having little trust in health services | *Boyd et al., 2011*  Jones, 2019 | 2 | *Boyd – Women described mistrust and fear in mental health services*  Jones 19 – a barrier was trust in health services | | | | **High confidence** |
| **1.2 Beliefs about HCPs** | | | | | | | | |
| 1.2.1 Not understanding HCPs’ role | Not understanding the roles of HCPs and how their roles related to PMH | Brealey et al., 2010; Button et al., 2017; Dennis & Chung-Lee, 2006; Hadfield & Wittkowski, 2017; Hewitt et al., 2009; Megnin-Viggars et al., 2015; Mollard et al., 2016; Morrell et al., 2016; Nilaweera et al., 2014; Sambrook Smith et al., 2019; Schmied et al., 2017; Scope et al., 2017 | 12 | Brealey – Women and HPs said it was important to be clear about HPs role so they’re not perceived as social agents  Button – Women did not understand the role of professionals  Dennis – Some women did not view health professionals as appropriate source of MH support  Hadfield 17 – Women were unclear about role of HPs  Hewitt – Roles of HVs could be clearer because they may be perceived as agents of social control  Megnin-Viggars – Women not clear about role of HVs  Mollard – Rural women did not expect mental health support from health provider  Morrell – Women did not understand the role of the service provider  Nilaweera – Women believed GPs and HV only involved in physical health  Sambrook-Smith – not knowing which HPs to contact was a barrier  Schmied – Women didn’t believe GP address mental health  Scope – Women didn’t think they could talk to nurse about MH problems. | | | | **Moderate confidence –** All about confusion over roles, but in different ways, who to talk to, are HPs agents of social control, can women talk to them about MH. |
| 1.2.2 Believing HCPs won’t be interested | Believing HCPs won't be interested in PMH | Bina, 2020; Hadfield & Wittkowski, 2017 | 2 | Bina – Women felt HPs were non responsive to their needs  Hadfield – Women felt they wouldn’t be taken seriously | | | | **Low confidence –** Bina is open to interpretation. Is this more about being dismissive |
| **1.3 Beliefs about perinatal mental illness** | | | | | | | | |
| **1.3.1 What is it?** | | | | | | | | |
| 1.3.1.1 What is perinatal mental illness? | Having poor or no knowledge about PMI | *Atif et al., 2019; Kerker et al., 2018*  Bina, 2020; Button et al., 2017; Dennis & Chung-Lee, 2006; Hadfield & Wittkowski, 2017; Hansotte et al., 2017; A. Jones, 2019; Lucas et al., 2019; Megnin-Viggars et al., 2015; Morrell et al., 2016; Newman et al., 2019; Sambrook Smith et al., 2019; Schmied et al., 2017; Scope et al., 2017; Staneva et al., 2015; Tobin et al., 2018; Watson et al., 2019 | 18 | *Atif – Lack of awareness about mental illness prevented disclosure*  *Kerker – Patients rarely acknowledged that stress and mental health were concerns in their life on screening tools*  Bina – Women struggled with the distinction between normal feelings of distress and exhaustion vs condition that justifies help seeking  Button – Women struggled to articulate their distress  Dennis – Some mothers lacked knowledge about postpartum depression, so were not aware they were suffering  Hadfield 17 – Barrier to support was not recognising symptoms  Jones 19 – Barrier was a lack of knowledge about perinatal depression  Lucas – Women didn’t know the meaning of depression  Megnin-Viggars – Lack of awareness about MI meant women could not explain their feelings  Morrell – Women did not see depression as something that happened in perinatal period  Newman – Women experienced difficulty in recognising their symptoms  Sambrook-Smith – Quote from a woman about not knowing the meaning of depression  Schmied – Migrant women rarely recognised or understood the symptoms of PND  Scope – barrier was not associating depression with perinatal period  Staneva – Women struggled to understand what was round and couldn’t explain their feelings  Tobin – Women didn’t understand PPD  Watson – Women from ethnic minority groups were not aware of PMI | | | | **High confidence –** All studies explained how women did not understand or have knowledge about PMI |
| 1.3.1.2 No language to describe perinatal mental illness | Not having the language to describe PMI | *Bina et al., 2018*  Brealey et al., 2010; Staneva et al., 2015; Tobin et al., 2018; Watson et al., 2019 | 5 | *Bina 18 – Many people do not know how to name the emotions they are feeling*  Brealey – Ethnic minority women could not describe their difficulties because of no culturally contextualised understanding of PND  Staneva – Some women couldn’t explain their feelings  Tobin – Some women used a different language to describe emotional distress  Watson – Women didn’t have the language to describe their symptoms | | | | **Moderate confidence –** All but one saying the same thing, Brealey is about cultural context |
| **1.3.2 Causes of perinatal mental illness** | | | | | | | | |
| 1.3.2.1 Spiritual/cultural causes | Believing that symptoms are caused by cultural or spiritual factors | *Atif et al., 2016; McCauley et al., 2019; Nakku et al., 2016*  Button et al., 2017; Schmied et al., 2017; Wittkowski et al., 2014 | 6 | *Atif – Quote explaining how women with MI are described as being possessed*  *McCauley – Attribution of causes to spiritual things*  *Nakku – Cultural influences and witchcraft as causes*  Button – Quote about cause of illness coming from god  Schmied – Became ill because they broke culturally traditions  Wittkowski – Women seen at risk of spirit attack and bewitchment | | | | **Moderate confidence –** All but Schmied are clear and saying the same thing |
| 1.3.2.2 External causes | Believing that symptoms are caused by external factors such as jobs, being a migrant | Bina, 2020; Button et al., 2017; Dennis & Chung-Lee, 2006; Lucas et al., 2019; Schmied et al., 2017; Staneva et al., 2015; Tobin et al., 2018; Watson et al., 2019 | 8 | Bina – External pressures as the cause  Button – external attribution as the cause  Dennis – Multiple stressors thought to lead to depressive symptoms  Lucas – Life circumstances as exacerbators  Schmied – Stress related socio-economic causes  Staneva – Women identified various stressors e.g. work and home environment  Tobin – Depressive symptoms to practical problems e.g. no time to rest  Watson – Financial problems, racism | | | | **Moderate confidence –** All about external factors, but could be split into closer and wider external factors |
| 1.3.2.3 Physical causes | Believing that symptoms are caused by physical factors such as tiredness and hormones | *O’Mahen & Flynn, 2008*  Bina, 2020; Button et al., 2017; Dennis & Chung-Lee, 2006; Forde et al., 2020; C. C. G. Jones et al., 2014; Newman et al., 2019; Sambrook Smith et al., 2019; Schmied et al., 2017; Staneva et al., 2015; Watson et al., 2019 | 13 | *O’Mahen – Women beliefs about genetic causes*  Bina 20 – Hormonal causes  Button – Lack of sleep and heavy cold  Dennis – Symptoms manifest as physical  Forde – Hormonal causes  Jones 14 – Physical fatigue  Newman – Not sleeping  Sambrook-Smith – Tiredness, hormones  Schmied – Physical exhaustion  Staneva - Hormones  Watson - Hormones | | | | **Moderate confidence –** All talk but Dennis talk about physical factors as causes |
| 1.3.2.4 A normal response to motherhood? | Believing symptoms are just a normal response to motherhood | *Williams et al., 2016*  Dennis & Chung-Lee, 2006; Giscombe et al., 2020; Jones et al., 2014; Sambrook Smith et al., 2019; Schmied et al., 2017; Slade et al., 2020; Sorsa et al., 2021; Viveiros & Darling, 2018 | 9 | *Williams – Seen as a normal part of pregnancy*  Dennis – Struggle is a normal part of motherhood  Giscombe – Women scored high on EPDS but did not think they were depressed  Jones 14 – Normal part of motherhood  Sambrook-Smith – Symptoms due to pregnancy and motherhood  Schmied – A normal part of motherhood  Slade – Was their traumatic birth normal?  Sorsa – Normal state after having a baby  Viveiros – Normal pregnancy feelings | | | | **Moderate confidence -**  Giscombe could be open to interpretation. Slade is slightly different but relevant. |
| **1.3.3 How to cope with symptoms** | | | | | | | | |
| 1.3.3.1 Ignore them | Women many deal with symptoms by ignoring them and assuming they will go away on their own | Bina, 2020; Hadfield & Wittkowski, 2017; Jones et al., 2014; Newman et al., 2019; Schmied et al., 2017; Slade et al., 2020 | 6 | Bina – Women felt they could deal with their symptoms on their own  Hadfield 17 – Some women believed their symptoms would resolve on their own  Jones 14 – Women coped by putting on a brave face  Newman – Some women waited before they sought help  Schmied – Women ignored their feelings, pushed them aside  Slade – Women avoided thinking about their feelings | | | | **Moderate confidence -**  All are about delaying help seeking by avoiding/ignoring but Bina was more about thinking they could deal with them themselves rather than specifically ignoring/avoiding |
| 1.3.3.2 Seek spiritual guidance | Women may cope with symptoms by seeking spiritual guidance | Hansotte et al., 2017; Kassam, 2019; Schmied et al., 2017; Watson et al., 2019 | 4 | Hansotte – Religion was a comfort to some women  Kassam – Religion was coping mechanism  Schmied – Cope by praying  Watson – Seeking spiritual support from religious leader | | | | **High confidence –** all about religious support |
| 1.3.3.3 Minimise them | Women may minimise or deny their symptoms | *Shakespeare et al., 2003*  Bina, 2020; Dennis & Chung-Lee, 2006; Forde et al., 2020; Hewitt et al., 2009; Holopainen & Hakulinen, 2019; Jones et al., 2014; Kassam, 2019; Megnin-Viggars et al., 2015; Schmied et al., 2017; Slade et al., 2020; Staneva et al., 2015; Tobin et al., 2018; Watson et al., 2019 | 14 | *Shakespeare – Some women covered up the way they were feeling*  Bina – women minimised the seriousness  Dennis – Women denied their feelings  Forde – Women concealed their symptoms  Hewitt – Women covered up their symptoms  Holopainen – Quote from woman about how only her husband knew the truth  Jones 14 – Women pretended everything was fine  Kassam – Women concealed their symptoms  Megnin-Viggars – Women concealed their illness  Schmied – Women ignored their symptoms, pushed them away  Slade – Women ignored symptoms  Staneva – Women denied their symptoms  Tobin  Watson – Women minimised their symptoms | | | | **High confidence –** All about ignoring, minimising, pretending symptoms don’t exist |
| **1.4 Deciding to seek help** | | | | | | | | |
| 1.4.1 Recognising something is wrong | The first step to seeking help for many women, was recognising that something is "wrong" | Bina, 2020; Button et al., 2017; Forde et al., 2020; Hadfield & Wittkowski, 2017; Slade et al., 2020; Staneva et al., 2015; Viveiros & Darling, 2018 | 8 | Bina – Recognition and awareness of difficulties were a trigger for help seeking  Button – Identification of a problem was the first step to help seeking  Forde – There was a “moment” when mothers realised they needed help  Hadfield – Women had to recognise something was wrong  Slade – Women reached breaking point before sourced support  Staneva – Women need to recognise their needs  Viveiros – Women needed to perceive the need for care | | | | **High confidence -**  very coherent theme |
| 1.4.2 Where do I go to seek help? | The next step to help seeking is then understanding where to go in order to seek help | *Ganann et al., 2019*  Bina, 2020; Dennis & Chung-Lee, 2006; Hansotte et al., 2017; Megnin-Viggars et al., 2015; Schmied et al., 2017; Sorsa et al., 2021; Tobin et al., 2018 | 9 | *Ganann – Women did not understand the health system and didn’t know what to ask for*  Bina – Women didn’t know where to go to get help  Dennis – Women didn’t know where to obtain assistance  Hansotte – Women didn’t know where to find treatment  Megnin-Viggars – Women did not know what to do with their symptoms of depression  Schmied – Quote from woman saying she didn’t know where to go, who to trust  Sorsa – Women didn’t know where to get help  Tobin – Women didn’t know where to get help or know how the system works | | | | **High confidence -**  very coherent theme |
| **1.5 Fear of judgement** | | | | | | | | |
| 1.5.1 Fear of being seen as a bad mum | Fear of being judged and being seen as a bad mother | Bina, 2020; Brealey et al., 2010; Button et al., 2017; Forde et al., 2020; Jones et al., 2014; Lucas et al., 2019; Slade et al., 2020; Sorsa et al., 2021; Viveiros & Darling, 2018 | 9 | Bina – Women worry to admit in case they are labelled as unfit mothers  Brealey – Women worry about being seen as an incompetent mother  Button – Quote about woman being worried to be judged to be a bad mum  Forde – Quote about woman being worried to be judged to be a bad mum  Jones 14 – Failure to be an ideal mother  Lucas – Young women felt judged to be a bad mum  Slade – Pressure of trying to be a perfect mother and fear of being judged  Sorsa – They didn’t want to be seen as a failure  Viveiros – Pressure to be a good mother | | | | **High confidence -**  very coherent theme |
| 1.5.2 Social services/ removal of child | Fear of social services involvement or their child being removed from their care | *Boyd et al., 2011; Feinberg et al., 2006; Shakespeare et al., 2003; Young et al., 2019*  Bina, 2020; Brealey et al., 2010; Button et al., 2017; Dennis & Chung-Lee, 2006; Evans et al., 2020; Forde et al., 2020; Hadfield & Wittkowski, 2017; Hewitt et al., 2009; A. Jones, 2019; Megnin-Viggars et al., 2015; Newman et al., 2019; Tobin et al., 2018; Watson et al., 2019  SH | 17 | *Boyd – Women are afraid their baby will be taken into care*  *Feinberg – Concern acknowledge of depressive symptoms would prompt child protective service involvement*  *Shakespeare – Women were scared they would lose the baby*  *Young -Fear of child protective services*  Bina 20 – Fear their baby would be taken away  Brealey – Fear of having their baby removed  Button – Fear of social services and baby being taken into care  Dennis – Fear of losing baby  Evans – Fear of social services  Forde – Worried baby would be taken away  Hadfield 17 – Fear of child being removed  Hewitt – Fear baby would be removed  Jones 19 – Worried about losing parental rights  Megnin-Viggars – Worried about losing custody  Newman – Fear of social services involvement  Tobin – Fear of child being taken into care  Watson – Fear child being removed from their care  SH- Fear of social services involvement | | | | **High confidence –** very coherent theme |
| **1.6 Logistics of accessing perinatal mental health care** | | | | | | | | |
| 1.6.1 Childcare | Lack of childcare as a barrier to PMH Care | *Boyd et al., 2011; Cox et al., 2017; Doering et al., 2017; Friedman et al., 2010*  Bina, 2020; Button et al., 2017; Dennis & Chung-Lee, 2006; Hansotte et al., 2017; Morrell et al., 2016; Newman et al., 2019; Sambrook Smith et al., 2019; Scope et al., 2017; Watson et al., 2019 | 14 | *Boyd – Can’t take their child to service and cannot afford childcare*  *Cox – Lack of childcare as a barrier*  *Doering – Lack of childcare as a barrier*  *Friedman – Lack of childcare as a barrier*  Bina 20 – Difficulty finding childcare  Button – Lack of childcare as a barrier  Dennis – No childcare  Hansotte – Lack of childcare  Morrell – Lack of childcare  Newman – limited childcare provision  Sambrook-Smith – limited childcare  Scope – improvement involved providing childcare  Watson – lack of childcare | | | | **High confidence -**  very coherent theme |
| 1.6.2 Timing of care | Timing of appointments and services offered | *Atif et al., 2019; Friedman et al., 2010*  Bina, 2020; Dennis & Chung-Lee, 2006; Newman et al., 2019; Scope et al., 2017; Watson et al., 2019 | 7 | *Atif 19 – Excessive home life demands meaning can’t always attend appointments*  *Friedman – Time constraints as a barrier*  Bina 20 – Lack of time to attend appointments  Dennis – Overburdened days, insufficient time, inconvenience of attending appointments  Newman – time of day of appointments  Scope- improvements would be to alter format and timing of sessions  Watson – waiting times and time of the day services provided | | | | **Moderate confidence –** About both mothers times and appointment times and how incompatible |
| 1.6.3 Location/ travel | Location of services or travel costs to get to services | *Cox et al., 2017; Doering et al., 2017; Eappen et al., 2018; Friedman et al., 2010; Masood et al., 2015; Nakku et al., 2016*  Bina, 2020; Hansotte et al., 2017; Mollard et al., 2016; Morrell et al., 2016; Newman et al., 2019; Tobin et al., 2018; Watson et al., 2019 | 13 | *Cox – Lack of transport*  *Doering – Lack of transport*  *Eappen – Travel to appointments*  *Friedman – Transport and transport costs*  *Masood – Lack of transport*  *Nakku – No transport or transport money*  Bina – Lack of transport, long distance to care  Hansotte – Lack of transport, geographically removed from care  Mollard – Lengthy travel times as a barrier  Morrell – Practical difficulties getting to appointments  Newman – inaccessible care and travel costs  Tobin – Lack of transport  Watson – Travel costs | | | | **High confidence –** Very coherent theme |
| **1.7 Social and family life** | | | | | | | | |
| 1.7.1 Social isolation or support | Woman’s experiences of social support or social isolation | Giscombe et al., 2020; Hansotte et al., 2017; A. Jones, 2019; C. Jones et al., 2014; Kassam, 2019; Lucas et al., 2019; Tobin et al., 2018; Viveiros & Darling, 2018; Watson et al., 2019 | 9 | Giscombe – No emotional support makes care challenging  Hansotte – No social relationships felt an additional burden when dealing with PPD  Jones 19 – Lack of appropriate support compounded feelings of isolation  Jones 14 – Lack of social support as a barrier to help seeking  Kassam – Sense of community ax  Lucas  Tobin  Viveiros – Emotional isolation and loneliness were barriers to help seeking  Watson | | | | **Moderate confidence** |
| 1.7.2 Family and friends’ beliefs | Woman’s family and friends’ beliefs about mental illness | *Atif et al., 2016, 2019; Boyd et al., 2011; Doering et al., 2017; Ganann et al., 2019; Higgins et al., 2018; Masood et al., 2015; Nakku et al., 2016; Nithianandan et al., 2016; Noonan et al., 2018; O’Mahen & Flynn, 2008; Pineros-Leano et al., 2015; Vik et al., 2009; Williams et al., 2016; Young et al., 2019*  Bina, 2020; Button et al., 2017; Dennis & Chung-Lee, 2006; Forde et al., 2020; Hadfield & Wittkowski, 2017; Holopainen & Hakulinen, 2019; A. Jones, 2019; Lucas et al., 2019; Nilaweera et al., 2014; Sambrook Smith et al., 2019; Schmied et al., 2017; Sorsa et al., 2021; Viveiros & Darling, 2018; Watson et al., 2019 | 30 |  | | | | **Moderate confidence** |
| 1.7.3 Additional personal difficulties | Personal difficulties outside of PMH such as unemployment | *Atif et al., 2016; Boyd et al., 2011; Kerker et al., 2018; Munodawafa et al., 2017; Rowan et al., 2010; Williams et al., 2016*  Hansotte et al., 2017 | 7 |  | | | | **Moderate confidence** |
| **1.8 Sociodemographic factors** | | | | | | | | |
| 1.8.1 Ethnicity | Woman’s ethnicity | Bina, 2020; Dennis & Chung-Lee, 2006; Hansotte et al., 2017; Watson et al., 2019 | 4 |  | | | | **Low confidence** |
| 1.8.2 Age | Woman’s age | Bina, 2020; Hansotte et al., 2017 | 2 |  | | | | **Very low confidence** |
| **1.9 Mental health factors** | | | | | | | | |
| 1.9.1 Previous experiences of mental health care | Previous experiences of mental health care | *O’Mahen & Flynn, 2008*  Button et al., 2017; Evans et al., 2020; Hadfield & Wittkowski, 2017; Hansotte et al., 2017; Watson et al., 2019 | 6 |  | | | | **Moderate confidence** |
| 1.9.2 Previous diagnoses or symptoms | Previous experiences of mental health symptoms or diagnoses | Bina, 2020; Sorsa et al., 2021 | 2 |  | | | | **Very low confidence** |
| 1.9.3 Current diagnoses or symptoms | Current experiences of mental health symptoms or diagnoses | *Chartier et al., 2015; Friedman et al., 2010; Hadfield et al., 2019; Young et al., 2019*  Sorsa et al., 2021; Viveiros & Darling, 2018 | 6 |  | | | | **Low confidence** |
| **2. HCPs** | | | | | | | | |
| **2.1 HCPs knowledge about PMH** | | | | | | | | |
| 2.1.1 HCPs knowledge about PMI | HCPs knowledge about PMH actual & perceived by women | *Beeber et al., 2009; Byatt et al., 2013; Ganann et al., 2019; Higgins et al., 2018; Judd et al., 2011; McCauley et al., 2019; McKenzie-McHarg et al., 2014; Noonan et al., 2018; Reed et al., 2014; Rowan et al., 2010*  Bina, 2020; Dennis & Chung-Lee, 2006; Megnin-Viggars et al., 2015; Morrell et al., 2016; Slade et al., 2020; Viveiros & Darling, 2018  SH | 17 | Moderate confidence | Moderate confidence | High confidence | Moderate confidence | **Moderate confidence** |
| 2.1.2 HCP’s knowledge about services/referral pathways | HCPs knowledge about PMH services and referral pathways actual & perceived by women | *Ganann et al., 2019; Higgins et al., 2018; Rowan et al., 2010*  Dennis & Chung-Lee, 2006; Hansotte et al., 2017; Sambrook Smith et al., 2019; Slade et al., 2020; Viveiros & Darling, 2018 | 8 | Moderate confidence | Low confidence | High confidence | High confidence | **High confidence** |
| 2.1.3 HCPs confidence | HCP's confidence in addressing PMH | *Atif et al., 2019; Bina et al., 2018; Cox et al., 2017; Fernandez y Garcia et al., 2011; Higgins et al., 2018; Munodawafa et al., 2017; Nithianandan et al., 2016; Ormsby et al., 2018; Reed et al., 2014*  SH | 9 | Moderate confidence confidence | Low confidence | High confidence | Moderate confidence | **Moderate confidence** |
| **2.2 Getting it right the first time** | | | | | | | | |
| 2.2.1 Being dismissive or normalising symptoms | HCPs dismissing or normalising symptoms | *Ganann et al., 2019*  Button et al., 2017; Dennis & Chung-Lee, 2006; Forde et al., 2020; Hadfield & Wittkowski, 2017; Hansotte et al., 2017; Megnin-Viggars et al., 2015; Newman et al., 2019; Sorsa et al., 2021; Watson et al., 2019  SH | 11 | Low confidence | Low confidence | High confidence | High confidence | **High confidence** |
| 2.2.2 Not recognising help seeking or PMI | HCPs not recognising help seeking or PMI | Bina, 2020; Button et al., 2017; Megnin-Viggars et al., 2015; Tobin et al., 2018; Watson et al., 2019 | 5 | Low confidence | Very low confidence | High confidence | Moderate confidence | **Moderate confidence** |
| 2.2.3 Focussing on infant | HCPs focussing mainly on the infant | Button et al., 2017; Megnin-Viggars et al., 2015 | 2 | Low confidence | Very low confidence | High confidence | Moderate confidence | **Low confidence** |
| 2.2.4 Making time | A HCP who makes time to address PMH concerns | *Feinberg et al., 2006; Myors et al., 2015; Noonan et al., 2018*  Bina, 2020; Button et al., 2017; Dennis & Chung-Lee, 2006; Hewitt et al., 2009; Megnin-Viggars et al., 2015; Slade et al., 2020; Viveiros & Darling, 2018; Watson et al., 2019 | 11 | Moderate confidence | Low confidence | Moderate confidence | High confidence | **Moderate confidence** |
| 2.2.5 Assessment specific behaviours | HCP's assessment specific behaviours, such as asking about PMH, carrying out in a tick box way, or in a personalised way | *Doering et al., 2017; Fernandez y Garcia et al., 2011; Nithianandan et al., 2016; Segre et al., 2014; Vik et al., 2009; Williams et al., 2016*  Brealey et al., 2010; Sambrook Smith et al., 2019; Schmied et al., 2017; Slade et al., 2020; Viveiros & Darling, 2018; Watson et al., 2019 | 12 | Moderate confidence | Low confidence | Moderate confidence | Moderate confidence | **Moderate confidence** |
| **2.3 HCPs’ attributes** | | | | | | | | |
| 2.3.1 Similar demographic characteristics | HCP having similar demographics to women | *Leger et al., 2015; Masood et al., 2015; Nithianandan et al., 2016; Shorey & Ng, 2019*  Dennis & Chung-Lee, 2006; Watson et al., 2019 | 6 | Moderate confidence | Low confidence | High confidence | Moderate confidence | **Moderate confidence** |
| 2.3.2 Culturally sensitive | HCP being sensitive to women from all cultures | Kassam, 2019; Nilaweera et al., 2014; Viveiros & Darling, 2018; Watson et al., 2019*;* | 4 | Moderate confidence | Very low confidence | Moderate confidence | Moderate confidence | **Low confidence** |
| 2.3.3 Valued characteristics | HCPs possessing valued characteristics Trustworthy, empathetic, kind, caring with a genuinine interest, and going above and beyond | *Atif et al., 2016, 2019; Boyd et al., 2011; Doering et al., 2017; Kerker et al., 2018; Kim et al., 2009; Munodawafa et al., 2017; Myors et al., 2015; Pugh et al., 2015; Shorey & Ng, 2019*  Brealey et al., 2010; Button et al., 2017; Dennis & Chung-Lee, 2006; Forde et al., 2020; Hadfield & Wittkowski, 2017; Hewitt et al., 2009; Jones, 2019; Megnin-Viggars et al., 2015; Morrell et al., 2016; Newman et al., 2019; Schmied et al., 2017; Slade et al., 2020; Staneva et al., 2015; Watson et al., 2019  SH | 25 | Moderate confidence | High confidence | High confidence | High confidence | **High confidence** |
| **3. Interpersonal** | | | | | | | | |
| 3.1 Trusting relationship and rapport | The development of a trusting relationship and rapport between HCP and women | *Atif et al., 2016; Doering et al., 2017; Feinberg et al., 2006; Ganann et al., 2019; Hadfield et al., 2019; Higgins et al., 2018; Kerker et al., 2018; Leger et al., 2015; Noonan et al., 2018; Shakespeare et al., 2003; Shorey & Ng, 2019; Willey et al., 2018; Williams et al., 2016; Young et al., 2019*  Bina, 2020; Brealey et al., 2010; Dennis & Chung-Lee, 2006; Hadfield & Wittkowski, 2017; Hewitt et al., 2009; Megnin-Viggars et al., 2015; Morrell et al., 2016; Scope et al., 2017; Tobin et al., 2018 | 23 | Moderate confidence | High confidence | Moderate confidence | High confidence | **High confidence** |
| 3.2 Language barriers | Difficulties in communicating due to language barriers | *Beeber et al., 2009; Doering et al., 2017; Ganann et al., 2019; Masood et al., 2015; Munodawafa et al., 2017; Nithianandan et al., 2016; Pineros-Leano et al., 2015; Segre et al., 2014; Willey et al., 2018; Williams et al., 2016*  Dennis & Chung-Lee, 2006; Hansotte et al., 2017; Megnin-Viggars et al., 2015; Sambrook Smith et al., 2019; Schmied et al., 2017; Watson et al., 2019  SH | 16 | Moderate confidence | Moderate confidence | High confidence | High confidence | **High confidence** |
| 3.3 Shared decision making | Shared decision making between HCP and woman | Hadfield & Wittkowski, 2017; Megnin-Viggars et al., 2015; Randall & Briscoe, 2018; Scope et al., 2017 | 4 | Low confidence | Very low confidence | Moderate confidence | Moderate confidence | **Low confidence** |
| 3.4 Open and honest communication | Open and honest communication between HCP and woman | *Doering et al., 2017; Shakespeare et al., 2003; Vik et al., 2009; Willey et al., 2018*  Brealey et al., 2010; Hadfield & Wittkowski, 2017; Hewitt et al., 2009; Schmied et al., 2017; Watson et al., 2019  SH | 9 | Moderate confidence | Low confidence | Moderate confidence | High confidence | **Moderate confidence** |
| **4. Organisational** | | | | | | | | |
| **4.1 Overall organisational aspects** | | | | | | | | |
| 4.1.1 Co location and buildings | Location of the service including co-location of different services within the same building | *Boyd et al., 2011; Cox et al., 2017; Judd et al., 2011; Munodawafa et al., 2017; Ormsby et al., 2018; Young et al., 2019;*  Bina, 2020  SH | 7 | Moderate confidence | Low confidence | Moderate confidence | Low confidence | **Low confidence** |
| 4.1.2 Service integration and collaborative working | Collaborative working across services  SH | *Atif et al., 2016; Bina et al., 2018; Boyd et al., 2011; Byatt et al., 2013; Feinberg et al., 2006; Ganann et al., 2019; Hadfield et al., 2019; Judd et al., 2011; Lind et al., 2017; Lomonaco-Haycraft et al., 2018; Myors et al., 2015; Nithianandan et al., 2016; Noonan et al., 2018; Rowan et al., 2010*  Newman et al., 2019; Sambrook Smith et al., 2019; Watson et al., 2019 | 17 | Moderate confidence | Moderate confidence | Moderate confidence | Moderate confidence | **Moderate confidence** |
| 4.1.3 Collaboration within services | Collaborative working within services  SH | *Ammerman et al., 2014; Cox et al., 2017; Eappen et al., 2018; Higgins et al., 2018; Judd et al., 2011; Kerker et al., 2018; Lind et al., 2017; McKenzie-McHarg et al., 2014; Munodawafa et al., 2017; Nithianandan et al., 2016; Ormsby et al., 2018; Segre et al., 2014; Willey et al., 2018*  Sambrook Smith et al., 2019 | 14 | Moderate confidence | Moderate confidence | Moderate confidence | Moderate confidence | **Moderate confidence** |
| 4.1.4 Adequate workforce provision/ HCPs workload | Ensuring and adequate workforce provision so PMH can be addressed | *Ammerman et al., 2014; Bina et al., 2018; Drozd et al., 2018; Feinberg et al., 2006; Ganann et al., 2019; Higgins et al., 2018; Kerker et al., 2018; Kim et al., 2009; McCauley et al., 2019; Nakku et al., 2016; Nithianandan et al., 2016; Noonan et al., 2018; Rowan et al., 2010; Vik et al., 2009; Willey et al., 2018*  Bina, 2020; Viveiros & Darling, 2018  SH | 17 | Moderate confidence | Moderate confidence | High confidence | High confidence | **High confidence** |
| 4.1.5 Clear assessment and referral process | Clear assessment and referral processes within the organisation | *Cox et al., 2017; Feinberg et al., 2006; Ganann et al., 2019; Judd et al., 2011; Kerker et al., 2018; Kim et al., 2009; Nithianandan et al., 2016; Rowan et al., 2010; Segre et al., 2014; Williams et al., 2016*  Bina, 2020  SH | 11 | Moderate confidence | Low confidence | Moderate confidence | Moderate confidence | **Moderate confidence** |
| 4.1.6 Provision of supervision | Supervision for HCPs | *Atif et al., 2019; Munodawafa et al., 2017; Vik et al., 2009* | 3 | Low confidence | Very low confidence | High confidence | Very low confidence | **Very low confidence** |
| 4.1.7 Training | Provision of training for all HCPs working with perinatal women | *Ammerman et al., 2014; Atif et al., 2016, 2019; Beeber et al., 2009; Bina et al., 2018; Boyd et al., 2011; Chartier et al., 2015; Doering et al., 2017; Drozd et al., 2018; Feinberg et al., 2006; Ganann et al., 2019; Judd et al., 2011; Kerker et al., 2018; Kim et al., 2009; Leger et al., 2015; Lind et al., 2017; McKenzie-McHarg et al., 2014; Munodawafa et al., 2017; Nakku et al., 2016; Nithianandan et al., 2016; Noonan et al., 2018; Reed et al., 2014; Rowan et al., 2010; Shorey & Ng, 2019; Willey et al., 2018; Williams et al., 2016*  Bina, 2020; Brealey et al., 2010  SH | 28 | Moderate confidence | High confidence | High confidence | High confidence | **High confidence** |
| 4.1.9 Organisational goals/guidelines | Clear organisational goals and guidelines | *Ammerman et al., 2014; Willey et al., 2018* | 2 | Low confidence | Very low confidence | Very low confidence | Very low confidence | **Very low confidence** |
| **4.2 Characteristics of PMH Care** | | | | | | | | |
| **4.2.1 Across the care pathway** | | | | | | | | |
| 4.2.1.1 Continuity of carer | Care that provides the same HCP along the care pathway | *Chartier et al., 2015; Higgins et al., 2018; Nithianandan et al., 2016; O’Mahen & Flynn, 2008; Rowan et al., 2010; Willey et al., 2018*  Brealey et al., 2010; Button et al., 2017; Dennis & Chung-Lee, 2006; Hadfield & Wittkowski, 2017; Megnin-Viggars et al., 2015; Sambrook Smith et al., 2019; Slade et al., 2020; Tobin et al., 2018; Viveiros & Darling, 2018; Watson et al., 2019  SH | 17 | Moderate confidence | Moderate confidence | High confidence | High confidence | **High confidence** |
| 4.2.1.2 Culturally sensitive care | Care that is culturally sensitive to women's needs | *Ganann et al., 2019; Nithianandan et al., 2016; Noonan et al., 2018; Shorey & Ng, 2019*  Bina, 2020; Brealey et al., 2010; Button et al., 2017; Dennis & Chung-Lee, 2006; Giscombe et al., 2020; Hadfield & Wittkowski, 2017; Hansotte et al., 2017; Hewitt et al., 2009; Jones, 2019; Kassam, 2019; Sambrook Smith et al., 2019; Schmied et al., 2017; Tobin et al., 2018; Viveiros & Darling, 2018; Watson et al., 2019 | 19 | Moderate confidence | Moderate confidence | High confidence | High confidence | **High confidence** |
| 4.2.1.3 Privacy & confidentiality | Care that is private and maintains women's confidentiality | *Atif et al., 2019; Feinberg et al., 2006; Higgins et al., 2018; Jallo et al., 2015; Nithianandan et al., 2016; O’Mahen & Flynn, 2008*  Giscombe et al., 2020 | 7 | Moderate confidence | Low confidence | Moderate confidence | Low confidence | **Low confidence** |
| 4.2.1.4 Dedicated person/ PMH Champion | Care that has a dedicated person or PMH champion | *Chartier et al., 2015; Ganann et al., 2019; Kim et al., 2009; Lomonaco-Haycraft et al., 2018; Nithianandan et al., 2016; Rowan et al., 2010; Willey et al., 2018* Bina, 2020; Megnin-Viggars et al., 2015 | 9 | Low confidence | Low confidence | Moderate confidence | Low confidence | **Low confidence** |
| 4.2.1.5 Logistical support | Logistical support for women including easily accessible location, childcare, travel costs | *Ganann et al., 2019; Hadfield & Wittkowski, 2017; Leger et al., 2015; Masood et al., 2015; Nakku et al., 2016; Nithianandan et al., 2016; Ormsby et al., 2018*  Button et al., 2017; Jones, 2019; Mollard et al., 2016; Newman et al., 2019; Scope et al., 2017; Watson et al., 2019 | 13 | Moderate confidence | Low confidence | High confidence | Moderate confidence | **Moderate confidence** |
| 4.2.1.6 Home delivery | Care that is delivered at home | *Ammerman et al., 2014; Beeber et al., 2009; Judd et al., 2011; Leger et al., 2015; Munodawafa et al., 2017; Myors et al., 2015*  Brealey et al., 2010; Hadfield & Wittkowski, 2017; Hansotte et al., 2017; Jones, 2019 | 10 | Moderate confidence | Low confidence | High confidence | Moderate confidence | **Moderate confidence** |
| 4.2.1.7 Hospital delivery | Care that is delivered in hopsital/medical setting | *Atif et al., 2019; Boyd et al., 2011; Kerker et al., 2018; Shakespeare et al., 2003*  Dennis & Chung-Lee, 2006 | 5 | Low confidence | Very low confidence | Low confidence | Low confidence | **Low confidence** |
| 4.2.1.8 Provision of information | Whether care provides information | Dennis & Chung-Lee, 2006; Hadfield & Wittkowski, 2017; Jones, 2019; Megnin-Viggars et al., 2015; Morrell et al., 2016; Randall & Briscoe, 2018; Schmied et al., 2017 | 7 | Low confidence | Low confidence | Moderate confidence | Moderate confidence | **Moderate confidence** |
| 4.2.1.9 Technology | The use of technology in care | *Doering et al., 2017; Feinberg et al., 2006; Fernandez y Garcia et al., 2011; Jallo et al., 2015; Kim et al., 2009; Lind et al., 2017; Noonan et al., 2018; Pineros-Leano et al., 2015; Shorey & Ng, 2019; Willey et al., 2018; Williams et al., 2016*  SH | 11 | Moderate confidence | Low confidence | High confidence | High confidence | **High confidence** |
| 4.2.1.10 Service inclusion criteria | Inclusion criteria of services | *Boyd et al., 2011; Ganann et al., 2019*  Viveiros & Darling, 2018 | 3 | Moderate confidence | Very low confidence | Moderate confidence | Low confidence | **Low confidence** |
| **4.2.2 Assessment specific characteristics** | | | | | | | | |
| 4.2.2.1 Wording of assessment tools | How assessment tools are worded | *Doering et al., 2017; Segre et al., 2014; Williams et al., 2016*  Brealey et al., 2010; Button et al., 2017; Hewitt et al., 2009 | 6 | Moderate confidence | Low confidence | High confidence | High confidence | **Moderate confidence** |
| 4.2.2.2 Acceptability of assessment/ screening | Whether assessment tools and assessment/screening in general are acceptable to women and HCPs | *Boyd et al., 2011; Doering et al., 2017; Feinberg et al., 2006; Ganann et al., 2019; Kim et al., 2009; Nithianandan et al., 2016; Segre et al., 2014; Shakespeare et al., 2003; Vik et al., 2009; Willey et al., 2018*  Brealey et al., 2010; Evans et al., 2020; Hewitt et al., 2009; Megnin-Viggars et al., 2015; Mollard et al., 2016; Sambrook Smith et al., 2019; Viveiros & Darling, 2018 | 17 | Moderate confidence | Moderate confidence | Moderate confidence | Moderate confidence | **Moderate confidence** |
| **4.2.3 Intervention characteristics** | | | | | | | | |
| 4.2.3.1 Opportunity to talk | Interventions that provide an opportunity to talk | Dennis & Chung-Lee, 2006; Evans et al., 2020; Hadfield & Wittkowski, 2017; Jones et al., 2014; Kassam, 2019; Morrell et al., 2016; Praetorius et al., 2020 | 7 | Low confidence | Low confidence | High confidence | Low confidence | **Low confidence** |
| 4.2.3.2 Individualised and person centred | Indiviudalised and person centred interventions/care | *Chartier et al., 2015; Doering et al., 2017; Ganann et al., 2019; Masood et al., 2015; McKenzie-McHarg et al., 2014; Noonan et al., 2018; O’Mahen & Flynn, 2008; Pugh et al., 2015; Segre et al., 2014; Shorey & Ng, 2019*  Evans et al., 2020; Hadfield & Wittkowski, 2017; Megnin-Viggars et al., 2015; Morrell et al., 2016; Schmied et al., 2017; Scope et al., 2017; Slade et al., 2020; Watson et al., 2019  SH | 19 | Moderate confidence | Moderate confidence | Moderate confidence | High confidence | **Moderate confidence** |
| 4.2.3.3 Appropriateness | Appropriateness of intervention being offered, from women and HCPs point of view | *Atif et al., 2019; Bina et al., 2018; Chartier et al., 2015; Drozd et al., 2018; Leger et al., 2015; McKenzie-McHarg et al., 2014; Munodawafa et al., 2017; Noonan et al., 2018; Ormsby et al., 2018; Pugh et al., 2015; Reed et al., 2014; Shorey & Ng, 2019*  Evans et al., 2020; Megnin-Viggars et al., 2015; Scope et al., 2017 | 15 | Moderate confidence | Moderate confidence | High confidence | Low confidence | **Moderate confidence** |
| 4.2.3.4 Flexible | Flexibility of intervention | *Atif et al., 2019; Bina et al., 2018; Ganann et al., 2019; Hadfield et al., 2019; Judd et al., 2011; Munodawafa et al., 2017; O’Mahen & Flynn, 2008; Pugh et al., 2015; Shorey & Ng, 2019*  Sorsa et al., 2021; Watson et al., 2019 | 11 | Moderate confidence | Low confidence | High confidence | Moderate confidence | **Moderate confidence** |
| 4.2.3.5 Group support | Group/peer support as an intervention | *Hadfield et al., 2019; Masood et al., 2015; Nakku et al., 2016*  Dennis & Chung-Lee, 2006; Evans et al., 2020; Hadfield & Wittkowski, 2017; Holopainen & Hakulinen, 2019; Jones et al., 2014; Megnin-Viggars et al., 2015; Schmied et al., 2017; Scope et al., 2017; Slade et al., 2020; Tobin et al., 2018; Watson et al., 2019  SH | 14 | Moderate confidence | Low confidence | Moderate confidence | High confidence | **Moderate confidence** |
| 4.2.3.7 Face to face delivery | Face to face delivery of intervention | *O’Mahen & Flynn, 2008; Pugh et al., 2015; Shorey & Ng, 2019*  Schmied et al., 2017  SH | 4 | Moderate confidence | Very low confidence | High confidence | High confidence | **Low confidence** |
| **5. Commissioners** | | | | | | | | |
| 5.1 Referral pathways | Clear referral pathways | *Ammerman et al., 2014; Boyd et al., 2011; Higgins et al., 2018; Nithianandan et al., 2016; Rowan et al., 2010; Willey et al., 2018*  SH | 6 | Moderate confidence | Low confidence | Moderate confidence | High confidence | **Moderate confidence** |
| 5.2 Lack of appropriate or timely services | Lack of appropriate and timely services to refer women onto | *Boyd et al., 2011; Doering et al., 2017; Ganann et al., 2019; Higgins et al., 2018; Kerker et al., 2018; Leger et al., 2015; Lomonaco-Haycraft et al., 2018; Munodawafa et al., 2017; Myors et al., 2015; Nakku et al., 2016; Noonan et al., 2018; Rowan et al., 2010; Williams et al., 2016*  Bina, 2020; Button et al., 2017; Jones, 2019; Jones et al., 2014; Megnin-Viggars et al., 2015; Newman et al., 2019; Sambrook Smith et al., 2019; Tobin et al., 2018; Viveiros & Darling, 2018  SH | 22 | Moderate confidence | High confidence | High confidence | High confidence | **High confidence** |
| 5.3 Financial complexities | Financial complexities including funding, and sourcing money and resources for services and financial reimbursement | *Cox et al., 2017; Feinberg et al., 2006; Friedman et al., 2010; Ganann et al., 2019; Kim et al., 2009; Lomonaco-Haycraft et al., 2018; Ormsby et al., 2018; Rowan et al., 2010*  SH | 8 | Moderate confidence | Low confidence | Moderate confidence | Moderate confidence | **Moderate confidence** |
| **6. Political** | | | | | | | | |
| 6.1 Immigration status | How the immigration status of women may impact their PMH care journey | *Cox et al., 2017; Ganann et al., 2019*  Bina, 2020; Giscombe et al., 2020; Hansotte et al., 2017; Kassam, 2019; Schmied et al., 2017; Tobin et al., 2018; Watson et al., 2019  SH | 9 | Moderate confidence | Low confidence | High confidence | High confidence | **High confidence** |
| 5.2 Economic status & healthcare costs | How the cost of healthcare, and women's economic status may impact their PMH care journey | *Atif et al., 2016; Boyd et al., 2011; Cox et al., 2017; Doering et al., 2017; Ganann et al., 2019; Lomonaco-Haycraft et al., 2018; Munodawafa et al., 2017; Nakku et al., 2016; Ormsby et al., 2018*  Bina, 2020; Hansotte et al., 2017; Jones, 2019; Kassam, 2019; Lucas et al., 2019; Tobin et al., 2018; Viveiros & Darling, 2018  SH | 16 | Moderate confidence | Moderate confidence | High confidence | Low confidence | **Moderate confidence** |
| **7. Societal** | | | | | | | | |
| 7.1 Stigma | Stigma related to mental illness | *Atif et al., 2016, 2019; Boyd et al., 2011; Chartier et al., 2015; Cox et al., 2017; Feinberg et al., 2006; Higgins et al., 2018; Kerker et al., 2018; McCauley et al., 2019; McKenzie-McHarg et al., 2014; Munodawafa et al., 2017; Myors et al., 2015; Nakku et al., 2016; Nithianandan et al., 2016; Noonan et al., 2018; O’Mahen & Flynn, 2008; Shakespeare et al., 2003; Vik et al., 2009; Williams et al., 2016; Young et al., 2019*  Bina et al., 2018; Button et al., 2017; Dennis & Chung-Lee, 2006; Giscombe et al., 2020; Hadfield & Wittkowski, 2017; Hansotte et al., 2017; Hewitt et al., 2009; Holopainen & Hakulinen, 2019; A. Jones, 2019; Kassam, 2019; Lucas et al., 2019; Megnin-Viggars et al., 2015; Mollard et al., 2016; Morrell et al., 2016; Nilaweera et al., 2014; Sambrook Smith et al., 2019; Schmied et al., 2017; Scope et al., 2017; Sorsa et al., 2021; Tobin et al., 2018; Viveiros & Darling, 2018; Watson et al., 2019  SH | 43 | Moderate confidence | High confidence | High confidence | High confidence | **High confidence** |
| 7.2 Culture | Cultural beliefs about mental illness and seeking and accessing help | *Atif et al., 2016; Bina et al., 2018; Boyd et al., 2011; Feinberg et al., 2006; Friedman et al., 2010; Ganann et al., 2019; Higgins et al., 2018; Masood et al., 2015; McCauley et al., 2019; Nakku et al., 2016; Noonan et al., 2018; Segre et al., 2014*  Brealey et al., 2010; Button et al., 2017; Dennis & Chung-Lee, 2006; Giscombe et al., 2020; Hansotte et al., 2017; Hewitt et al., 2009; Holopainen & Hakulinen, 2019; Jones, 2019; Kassam, 2019; Megnin-Viggars et al., 2015; Praetorius et al., 2020; Sambrook Smith et al., 2019; Schmied et al., 2017; Staneva et al., 2015; Tobin et al., 2018; Viveiros & Darling, 2018; Watson et al., 2019; Wittkowski et al., 2014  SH | 30 | Moderate confidence | High confidence | High confidence | High confidence | **High confidence** |
| 7.3 Maternal norms | Maternal norms of being a "good mother" and a "strong woman" | *Shakespeare et al., 2003; Williams et al., 2016*  Bina, 2020; Brealey et al., 2010; Button et al., 2017; Dennis & Chung-Lee, 2006; Hadfield & Wittkowski, 2017; Hansotte et al., 2017; Hewitt et al., 2009; Holopainen & Hakulinen, 2019; Jones et al., 2014; Kassam, 2019; Lucas et al., 2019; Megnin-Viggars et al., 2015; Mollard et al., 2016; Newman et al., 2019; Nilaweera et al., 2014; Praetorius et al., 2020; Sambrook Smith et al., 2019; Schmied et al., 2017; Slade et al., 2020; Sorsa et al., 2021; Staneva et al., 2015; Viveiros & Darling, 2018; Watson et al., 2019  SH | 26 | Low confidence | High confidence | High confidence | High confidence | **High confidence** |

# *Supplementary Materials 6:* GRADE-CERQual Adequacy rating table

| Source | Theme | Summary of review finding | Studies contributing to finding | Adequacy | Overall rating |
| --- | --- | --- | --- | --- | --- |
| **1** | Additional personal difficulties | Difficulties that women may have that are not related to perinatal mental illness, such as poverty can prevent successful service implementation | Ganann 2019  Rowan 2010  Kerker 2018  Munodawafa 2017  Williams 2016  Atif 2016  Atif 2020  Boyd 2011  Cox 2017  Nakku 2016 | **Richness -** Rowan, Kerker and Cox – vague, descriptive data  **Quantity -**  10 studies | Minor – most studies provide rich relevant data, and there are a large amount of supporting studies |
| **1** | Family  Family and friends | Negative beliefs from women’s family and friends can prevent successful implementation, help-seeking and women’s access to services | Atif 2016  Atif 2020  Boyd 2011  Doering 2017  Gannan 2019  Higgins 2018  Masood 2015  Nakku 2016  Nithianden  Noonan  o mahen  Pineros Leano  Williams  Vik 2009  Young 2019 | **Richness -** Higgins was very vague, reported as a stat. Hansotte was a bit vague. Viveiros is not detailed  **Quantity -**  30 studies | **None to very minor -**  30 studies, majority are very rich in data, very descriptive |
| **2** |  |  | Bina, 2020; Button et al., 2017; Dennis & Chung-Lee, 2006; Forde et al., 2020; Hadfield & Wittkowski, 2017; Hansotte et al., 2017; Holopainen & Hakulinen, 2019; Jones, 2019; Lucas et al., 2019; Nilaweera et al., 2014; Schmied et al., 2017; Smith et al., 2019; Sorsa et al., 2021; Viveiros & Darling, 2018; Watson et al., 2019 |  |  |
| **2** | Social isolation | Social isolation can exacerbate women’s symptoms, but can also be exacerbated by social isolation. This can affect help seeking and women’s experiences of care | Bina, 2020; Giscombe et al., 2020;  Jones, 2019;  Jones et al., 2014;  Kassam, 2019;  Tobin et al., 2018;  Viveiros & Darling, 2018  Watson | **Richness -**  bina not rich, one statement, viveiros statement, jones 19 3 words  **Quantity =** 8 | **Moderate -**  3 very non descriptive studies, but the rest are fine, 8 is a fair quantity but I think with so many studies included it is still a small amount |
| **1** | Beliefs about the causes of mental illness | When women believe their symptoms are caused by a biological predisposition, or that their symptoms are caused by spiritual, physical or external factors, or are a normal response to motherhood this hinders implementation and prevents women help seeking and accessing services. | O'Mahen 2015  Feinberg 2006 | **Richness -**  both are rich  **Quantity =**  2 - poor | **Moderate -**  rich, but not enough studies |
| **2** | Spiritual |  | Schmied et al., 2017; Wittkowski et al., 2014 | **Richness: ­**fine, less rich than above  **Quantity = 2** | **Serious:**  rich but not as rich as above and only 2 studies |
| **2** | External |  | Bina, 2020; Button et al., 2017; Dennis & Chung-Lee, 2006; Lucas et al., 2019; Schmied et al., 2017; Staneva et al., 2015; Tobin et al., 2018; Watson et al., 2019 | **Richness -**  button is one statement  **Quantity =** 8 | **Moderate -**  most were rich but 8 studies is very limited |
| **2** | Physical |  | Bina, 2020; Button et al., 2017; Dennis & Chung-Lee, 2006; Forde et al., 2020; Jones et al., 2014; Newman et al., 2019; Schmied et al., 2017; Smith et al., 2019; Staneva et al., 2015; Watson et al., 2019 | **Richness:**  Watson very vague  **Quantity: 10** | **Minor:** most descriptively rich, 10 studies limited but okay |
| **2** | Normal |  | Dennis & Chung-Lee, 2006; Giscombe et al., 2020; Jones et al., 2014; Schmied et al., 2017; Slade et al., 2020; Smith et al., 2019; Sorsa et al., 2021 | **Richness** all but viveiros were rich  **Quantity: 7** | **Moderate -**  most were rich but 7 studies is very limited |
| **2** | Dealing with symptoms - ignore | Depending on how women view their symptoms will affect how they deal with their symptoms. When women choose to ignore their difficulties, minimise their symptoms or seek spiritual guidance this is a barrier to help-seeking and access | Bina, 2020; Hadfield & Wittkowski, 2017; Jones, 2019; Newman et al., 2019; Schmied et al., 2017; Slade et al., 2020 | **Richness** all rich  **Quantity: 6** | **Moderate -**  most were rich but 6 studies is very limited |
| **2** | seeking spiritual guidance |  | Hansotte et al., 2017; Kassam, 2019; Schmied et al., 2017; Watson et al., 2019 | **Richness:** all good  **Quantity:**  4 | **Serious:**  rich but very limited number of studies, more research needed |
| **2** | Symptom minimisation |  | Bina, 2020; Dennis & Chung-Lee, 2006; Forde et al., 2020; Hewitt et al., 2009; Holopainen & Hakulinen, 2019; Jones et al., 2014; Kassam, 2019; Lucas et al., 2019; Megnin-Viggars et al., 2015; Schmied et al., 2017; Staneva et al., 2015; Watson et al., 2019 | **Richness:** Dennis is one statement, Watson also one statement  **Quantity:**  12 | **Minor:**  2 vague studies, but 12 supporting studies |
| **2** | Recognising something is wrong | Women have to recognise something is wrong before they seek help | Bina, 2020; Button et al., 2017; Forde et al., 2020; Hadfield & Wittkowski, 2017; Hansotte et al., 2017; Slade et al., 2020; Sorsa et al., 2021; Staneva et al., 2015; Viveiros & Darling, 2018 | **Richness:** Hadfield not detailed, Forde not massively detailed, Hansotte equally undetailed  **Quantity:**  9 | **Moderate -**  most were rich but limited data overall |
| **2** | Not knowing where to go | Not having enough information to know where to go is a barrier to help seeking | Bina, 2020; Dennis & Chung-Lee, 2006; Hansotte et al., 2017; Jones, 2019; Megnin-Viggars et al., 2015; Schmied et al., 2017; Smith et al., 2019; Sorsa et al., 2021; Tobin et al., 2018 | **Richness:** Bina, Hansotte, Megnin viggars, Dennis, Sambrook Smith, Sorsa did not provide particularly detailed descriptions  **Quantity:**  9 | **Moderate:**  few studies and not all were rich |
| **2** | Beliefs about health services | Beliefs about health services are also a barrier to helpseeking and access, such as believing services only offer medication, are stretched, HCPs wont care or that they not sure if the HCP they are talking with are equipped to deal with their symptoms, | N/A |  |  |
| **2** | Beliefs about health services – medication only |  | Bina, 2020; Button et al., 2017; Jones, 2019; Megnin-Viggars et al., 2015; Nilaweera et al., 2014; Sorsa et al., 2021; Tobin et al., 2018 | **Richness:** Sorsa does not provide rich data  **Quantity:**  7 | **Moderate -**  most were rich but limited data overall |
| **2** | Beliefs about health services - stretched |  | Hadfield & Wittkowski, 2017 | **Richness:** Very detailed  **Quantity:**  1 | **Severe:**  not enough data to make conclusion – only one study |
| **2** | Beliefs about HCPs – what is their role |  | Brealey et al., 2010; Button et al., 2017; Dennis & Chung-Lee, 2006; Hadfield & Wittkowski, 2017; Hewitt et al., 2009; Megnin-Viggars et al., 2015; Mollard et al., 2016; Morrell et al., 2016; Nilaweera et al., 2014; Schmied et al., 2017; Scope et al., 2017; Smith et al., 2019 | **Richness:** Dennis is not very detailed. 2 which lack additional content but it is clear what the message is  **Quantity:**  12 | **Minor -**  most clear and there is quite a lot of supporting data |
| **2** | Beliefs about HCPs – they won’t be interested |  | Bina, 2020; Hadfield & Wittkowski, 2017 | **Richness:** both provide rich data  **Quantity:**  2 | **severe -**  rich, but not enough studies (n = 2) |
| **1** | Inability to attend | Logistical issues effect implementation, help seeking and access to care. These logistical issues include, childcare, travel, timing of appointments | Young 2019  Atif 2016  Pugh 2015  Atif 2020  Boyd 2011  Chariter 2015  Cox 2017  Doering 2017  Friedman 2011  Hadfield 2019  Eappen 2018  Masood 2015 | **Richness:** Atif 16 is vague, as is Chartier & Hadfield – could be more detailed  **Quantity:**  12 | **Minor -**  lots of data, most is clear |
| **2** | Logistics – childcare |  | Bina, 2020; Button et al., 2017; Dennis & Chung-Lee, 2006; Hansotte et al., 2017; Morrell et al., 2016;Jones 19 Newman et al., 2019; Scope et al., 2017; Smith et al., 2019; Tobin et al., 2018; Watson et al., 2019 | **Richness:** None of the studies describe this in detail, but it is well supported by data (lots of it and very clear)  **Quantity:**  11 (15 including studies above) | **Minor:**  lots of data and all is saying the same thing |
| **2** | Logistics – timing |  | Bina, 2020; Dennis & Chung-Lee, 2006; Newman et al., 2019; Scope et al., 2017; Watson et al., 2019 | **Richness:** None of the studies describe this in detail  **Quantity:**  5 (9 including studies above) | **Minor:**  data and all is saying the same thing |
| **2** | Logistics – location/travel |  | Bina, 2020; Hansotte et al., 2017; Jones, 2019; Mollard et al., 2016; Morrell et al., 2016; Newman et al., 2019; Sorsa et al., 2021; Tobin et al., 2018; Watson et al., 2019 | **Richness:** None of the studies describe this in detail  **Quantity:** 9 (12 including above) | **Minor:**  lots of data and all is saying the same thing |
| **1** | Symptoms of psychological difficulty | Psychological factors such as fear of judgement, feeling psychologically ready to take part in care, symptoms of psychological difficulties, and fear of social services involvement prevent implementation, help seeking and access to PNMH care | Friedman 2011  Boyd 2011  Hadfield 2019  BINA (2)  Viveirose (2) | **Richness:** all rich  **Quantity: 5** | **Moderate -**  not enough data to draw conclusions |
| **2** | Being seen as bad mum |  | Bina, 2020; Brealey et al., 2010; Button et al., 2017; Forde et al., 2020; Jones et al., 2014; Lucas et al., 2019; Schmied et al., 2017; Slade et al., 2020; Sorsa et al., 2021; Viveiros & Darling, 2018; Watson et al., 2019 | **Richness:** viveiros is lacking in detail  **Quantity: 10** | **Minor:**  data and all is saying the same thing |
| **2** | Social services |  | Bina, 2020; Brealey et al., 2010; Button et al., 2017; Dennis & Chung-Lee, 2006; Evans et al., 2020; Forde et al., 2020; Hadfield & Wittkowski, 2017; Hewitt et al., 2009; Jones, 2019; Megnin-Viggars et al., 2015; Newman et al., 2019; Tobin et al., 2018; Watson et al., 2019 | **Richness:** all rich  **Quantity:** 13 | **None/V minor -**  all data is rich and all is saying the same thing and there is a fair bit of data supporting this |
| **SH** | Fear of social services involvement |  | Stakeholder group meeting |  |  |
| **1** | Psychological readiness |  | Leger 2005  Chartier 2015 | **Richness:** Neither particularly rich  **Quantity: 2** | **Serious -**  only 2 studies, only a sentence for each study |
| **2** | Ethnicity | Demographic factors such as age (older women are more likely to seek help), ethnicity (white women are more likely to seek help), and previous experiences with mental health services (those with previous diagnoses and positive experiences of MHS are more likely to seek help) impacted helpseeking and access to PNMH Care | Bina, 2020; Dennis & Chung-Lee, 2006; Hansotte et al., 2017; Watson et al., 2019 | **Richness:** all rich  **Quantity: 4** | **Moderate -**  not enough data to draw conclusions |
| **2** | Age |  | Bina, 2020; Hansotte et al., 2017 | **Richness:**  not particularly rich  **Quantity: 2** | **Serious -**  only 2 studies, only a sentence for each study |
| **1** | Previous experiences |  | O’mahen | **Richness:** Relatively rich and detailed  **Quantity: 8** |  |
| **2** |  |  | Button et al., 2017; Evans et al., 2020; Hadfield & Wittkowski, 2017; Hansotte et al., 2017; Jones, 2019; Sorsa et al., 2021; Watson et al., 2019 |  | **Moderate -**  rich but not enough data to draw conclusions |
| **2** | Previous diagnoses |  | Bina, 2020; | **Richness: No**  **Quantity: 1** | **Serious -**  only 1 study, not rich, more data needed |
|  | **HCPs** |  |  |  |  |
| 1 | Characteristics of healthcare professionals (n = 19) | The way healthcare professionals acted towards women. For example, open, non-judgmental, willing to listen, motivated and interested healthcare professionals were valued by women, aided implementation. Trustworthy, readily available, helpful, interested, nonjudgemental, understanding, caring, positive, empathetic, warm, above and beyond were all facilitators to PNMH access. | Atif  Boyd  Chartier  Doering  Feinberg  Kerker  Gannan  Higgins  Judd  Kim  Leger  Masood  Munodawaf  Myors  Nithian  Noonan  Pugh  Shorey  Willioams  Atif 2020 | **Richness:** Boyd is not detailed, Higgins not detailed, Judd not detailed, Kim not detailed, Brealey is not very detailed  **Quantity: 34** | **None/V Minor -**  although a couple aren’t very detailed, there are over 30 studies all saying the same thing, it is enough to make a conclusion |
| 2 | Characteristics of HCPs |  | Brealey et al., 2010; Button et al., 2017; Dennis & Chung-Lee, 2006; Forde et al., 2020; Hadfield & Wittkowski, 2017; Hewitt et al., 2009; Megnin-Viggars et al., 2015; Morrell et al., 2016; Newman et al., 2019; Schmied et al., 2017; Slade et al., 2020; Staneva et al., 2015; Viveiros & Darling, 2018 |  |  |
| 2 | HCP being dismissive or normalising symptoms | This prevented women from accessing care | Button et al., 2017; Dennis & Chung-Lee, 2006; Forde et al., 2020; Hadfield & Wittkowski, 2017; Hansotte et al., 2017; Megnin-Viggars et al., 2015; Newman et al., 2019; Sorsa et al., 2021; Watson et al., 2019 | **Richness:** All provide detailed data  **Quantity: 9** | **Minor:**  including SH support |
| 2 | The way the HCP delivers the care | HCPs focussing on the infant, not asking about PNMH or asking in a tick-box way, not treating women as individuals and not being culturally sentitive were barriers to care | Button et al., 2017; Forde et al., 2020; Kassam, 2019; Megnin-Viggars et al., 2015; Nilaweera et al., 2014; Schmied et al., 2017; Slade et al., 2020; Smith et al., 2019; Sorsa et al., 2021 | **Richness:** all provide detailed data  **Quantity: 9** | **Moderate:**  not enough data to make a conclusion |
| 1 | Collaborative working (n = 18) | Healthcare professionals working together to improve care for women. | Ammerman  Boyds  Byatt  Cox  Eappen  Feinberg  Ganann  Higgins  Judd  Kerker  Lind  Lomonaco  Munodawafa  Myors  Nithianden  Noonan  Rowan  Willey | **Richness:** Higgins is not v detailed, neither is Lind, same with Nithianden  **Quantity: 18** | **Minor:**  all saying that working together, organisational links are important |
| 1 | Communication between healthcare professionals (n = 10) | Clear communication amongst healthcare professionals. | Bina  Byatt  Cox  Eappen  Lind  Mckenzie  Nithiandan  Orsmby  Riwan  Segre | **Richness:** all provide detailed data apart from Segre  **Quantity: 10** | **Minor:**  all saying the same thing |
| 1 | Confidence of healthcare providers  (n = 12) | Healthcare professional’s belief in their ability to provide the care being offered to women. | Atif 2020  Beeber  Bina  Cox  Fernandez  Higgins  Munodawara  Nithianden  Ormsby  Reed  Vik | **Richness:** Beeber is lacking detail  **Quantity:** 11 | **Minor:**  all saying the same thing |
| 1 | Dedicated person to act as women’s advocate (n = 7) | Someone who can explain the process of the service and who is there for women throughout the care pathway. | Lomonoco haycroft  Chartier  Kim  Ganann  Nithian  Rowan  Willey | **Richness:** Kim provides hardly any detail  **Quantity:8** | **Moderate:** more data is needed before conclusions can be made |
| 1 | Heavy workload (n = 20) | A heavy workload or not enough time to provide the care being offered by the service was a barrier to implementation and HCP appearing to busy to women was a barrier to PNMH access. | Ammerman  Bina  Drozd  Eappen  Feinberg  Fernandez  Gannan  Higgins  Kerker  Kim  McCauley  McKenzie  Nakku  Nithianden  Noonan  Rowan  Shorey  Vik  Willey | **Richness:** Kim provides hardly any detail, Bina provides little detail  **Quantity: 26** | **None/V minor:** lots of data, all saying the same thing |
| 2 | HCP appearing too busy from women’s perspective |  | Bina, 2020; Button et al., 2017; Dennis & Chung-Lee, 2006; Hewitt et al., 2009; Megnin-Viggars et al., 2015; Slade et al., 2020; Viveiros & Darling, 2018; Watson et al., 2019 |  |  |
| 1 | Knowledge (n = 8) | Healthcare professionals not knowing about PNMI or the services available was a barrier to implementation, and women perceiving this to be the case was a barrier to access | Ganann  Higgins  McCauley  McKenzie McHarg  Ormsby  Reed  Rowan  Beeber | **Richness:** Ormsby is vague  **Quantity: 9** | **Minor:** if you take lack of knowledge generally then there are 16 studies that show this |
| 2 | Women’s perception of HCPs knowledge about PNMH |  | Dennis & Chung-Lee, 2006; Forde et al., 2020; Hansotte et al., 2017; Jones, 2019; Megnin-Viggars et al., 2015; Morrell et al., 2016 |  |  |
| 2 | Women’s perception of HCPs knowledge of services/referral pathways |  | Dennis & Chung-Lee, 2006; Smith et al., 2019; Viveiros & Darling, 2018 | **Richness: all fine**  **Quantity: 7** |  |
| 1 | Supervision/Support (n = 6) | The opportunity for healthcare professionals to have a place to reflect and raise issues. | Boyd  Gannan  Munodawafa  Nakku  Vik  Nithianden | **Richness:** all provide an okay level of detail  **Quantity: 6** | **Moderate:**  not the most detailed or quantity full theme, not enough evidence to draw conclusions |
| 1 | Training (n = 27) | The opportunity for healthcare professionals to learn about perinatal mental illness and the care they are providing to women- poor or no training was associated with poor or no implementation. | Ammerman  Atif 2016  Atiff 2020  Beeber  Boyd  Chartier  Doering  Drozd  Eappen  Feinberg  Gannan  Kerker  Judd  Kim  Lind  Mccaluey  Mckenzie  Munodowar  Nithianden  Noonan  Reed  Rowan  Shorey  Willey  Williams | **Richness:** Chartier doesn’t provide a high level of detail  **Quantity: 25** | **None/V minor:** a lot of studies, providing a good level of detail about training |
| 2 | HCP not recognising help-seeking | This impacted women’s access to care | Bina, 2020; Button et al., 2017; Megnin-Viggars et al., 2015; Tobin et al., 2018; Watson et al., 2019 | **Richness:** Most studies only provide a basic level of detail  **Quantity: 5** | **Moderate:** Not enough detailed data to draw a conclusion. |
|  | Interpersonal |  |  |  |  |
| 1 | Language barriers (n = 10) | Difficulties in communication due to women and healthcare professionals speaking different languages affected implementation and access. | Doering  Boyd  Gannan  Masood  Munodowafa  Nithiaden  Pinero-leano  Segre  Willey  Williams | **Richness:** Data is detailed  **Quantity: 16** | **No/V minor:** detailed data provided across 2 systematic reviews, all saying the same thing + support from stakeholder meetings |
| 2 |  |  | Button et al., 2017; Dennis & Chung-Lee, 2006; Hansotte et al., 2017; Megnin-Viggars et al., 2015; Schmied et al., 2017; Smith et al., 2019 |  |  |
| SH |  |  | Discussions of this in the stakeholder meetings |  |  |
| 1 | Open and honest communication (n = 11) | Women and healthcare professionals being able to speak openly and honestly without fear of judgement – aids implementation . Women like to be informed of what will he happening, and for HCPs to listen to them this aids access | Doering  Shakespeare  Williams  Vik  Shorey  Beeber  Boyd  Willey  Gannan | **Richness:** Data is quite rich, but not as rich as other themes  **Quantity: 13** | **Minor issues:** communication is important for women and HCPs but some depth lacking |
| 2 | Communication |  | Brealey et al., 2010; Forde et al., 2020; Hadfield & Wittkowski, 2017; Hewitt et al., 2009 |  |  |
| 2 | Information provision | Women like to have information shared with them, this aids access | Dennis; Hadfield; Jones Megnin-Viggars et al., 2015; Randall & Briscoe, 2018; Slade et al., 2020; Sorsa et al., 2021; | **Richness:** Jones is not detailed  **Quantity: 8** | **Moderate:** There are more detailed themes, and only 8 studies supporting this theme |
| 2 | Shared decision making | Women like to make decisions about their care with HCPs, this aids access | Bina, 2020; Hadfield & Wittkowski, 2017; Megnin-Viggars et al., 2015; Morrell et al., 2016; Randall & Briscoe, 2018; Scope et al., 2017 | **Richness:** Data is moderately rich  **Quantity: 6** | **Moderate:** There are more detailed themes, and only 6 studies supporting this theme |
| 1 | Privacy and confidentiality (n = 8) | Women and healthcare professionals being able to interact in privacy. | Doering  Feinberg  Higgins  Jallo  Masood  Omahen  Pineros-Leano  Pugh | **Richness:** Higgins, v v lacking in detail  **Quantity: 8** | **Moderate:** There are more detailed themes, and only 8 studies supporting this theme |
| 1 | Trusting relationship (n = 15) | Women feeling safe with healthcare professionals to be able to be open and honest about their feelings this aided implementation and access. | Atif 2016  Doering  Feinberg  Gannan  Hadfield  Higgins  Kerker  Leger  Noonan  Shakespeare  Shorey  Vik  Willey  Williams  Young | **Richness:** Most data is well detailed  **Quantity: 25** | **No/V minor:** There is a lot of data saying the same thing |
| 2 | Relationship and rapport |  | Bina, 2020; Brealey et al., 2010; Dennis & Chung-Lee, 2006; Hadfield & Wittkowski, 2017; Hewitt et al., 2009; Megnin-Viggars et al., 2015; Morrell et al., 2016; Scope et al., 2017; Slade et al., 2020; Tobin et al., 2018 |  |  |
|  | Medication |  |  |  |  |
|  | HCPs reluctance | Healthcare professionals having little confidence in prescribing medication or knowing which medications are safe during pregnancy and breastfeeding | Ganann  Byatt  Noonan | **Richness:** There is detailed data supporting this theme  **Quantity: 3** | **Severe:** Not enough data to draw conclusions |
| 1 | Women’s reluctance | Women not wanting to take medication affects implementation of care and access. | Ganan  Doering  Williamns  Young | **Richness:** Jones did not provide rich, detailed data  **Quantity: 12** | **None/V Minor –** Multiple studies showing the same thing |
| 2 |  |  | Dennis, Tobin, Jones & Sorsa, Megnin Viggers, Hadfield, Bina and Button |  |  |
|  | **Ideal care** |  |  |  |  |
| 1 | **Appropriateness**  Appropriateness of care (n = 6)  Appropriateness | Care sensitively designed to fit women’s needs improved implementation and access | Chartier  Ganann  Noonan | **Richness:** Data is relevatively rich  **Quantity: 6** | **Moderate:** Not enough data to draw conclusions |
| 2 |  |  | Evans et al., 2020; Megnin-Viggars et al., 2015; Scope et al., 2017; |  |  |
| 2 | Culturally appropriate | In line with this care needed to be culturally appropriate to fit to women’s individual cultural needs | Button et al., 2017; Dennis & Chung-Lee, 2006; Hadfield & Wittkowski, 2017; Jones, 2019; Schmied et al., 2017; Smith et al., 2019; Tobin et al., 2018; Watson et al., 2019 | **Richness:** Most of the data apart from Jones is rich  **Quantity: 8** | **Moderate:** although the findings are detailed, there is not enough data to draw firm conclusion |
| 1 | **Woman-centred**  Choice (n = 6) | Women being able to choose their care improved implementation | Chartier  Doering  Ganann  Ormsby  Vik  Segre | **Richness:** Data is moderately rich  **Quantity: 6** | **Moderate:** Not enough data to draw conclusions |
| 1 | Fitting in with women’s lifestyle (n = 3) | Interventions that fit easily into women’s lifestyles aided implementation. | Chartier  Omahen  Pugh | **Richness:** Moderately rich  **Quantity: 3** | **Severe** Not enough data to draw conclusions |
| 1 | Flexibility (n = 13) | Flexibility of care improved implementation | Bina  Doering  Eappen  Ganann  Hadfield  Judd  Munoda  Nithian  Omahen  Pugh  Shorey  Williams  Atif 2020 | **Richness:** Some studies are richer than others, but generally it is a detailed theme  **Quantity: 13** | **Minor:** moderate amount of studies |
| 1 | Patient centred (n = 5) | Being aware of patient’s needs and providing care with women at the centre aided implementation and access  Relevance to women improved implementation |  |  |  |
| 1 | Relevance to women (n = 2) |  | Omahen  Pugh | **Richness: Moderately rich**  **Quantity:2** | **Severe:**  only 2 studies, more data needed – also linked to appropriateness |
| 2 | Individualised |  | Evans et al., 2020; Hadfield & Wittkowski, 2017; Megnin-Viggars et al., 2015; Morrell et al., 2016; Schmied et al., 2017; Scope et al., 2017; Slade et al., 2020; Sorsa et al., 2021; Viveiros & Darling, 2018; Watson et al., 2019 | **Richness:** Most data is rich  **Quantity: 10** | **Minor:**  enough data to draw a conclusion, but literature not totally saturated |
| 2 | Length | Care needed to be at an appropriate length for women, not to finish too early to aid access | Hadfield & Wittkowski, 2017; Morrell et al., 2016; Schmied et al., 2017; Watson et al., 2019 | **Richness:** Data is quite detailed  **Quantity: 4** | **Severe:** Despite detailed studies, more research is needed |
| 1 | Timing | Implementation was aided when the timing fitted in with women’s needs | Pugh  Segre  Leger  Masood | **Richness:** Data is moderately rich  **Quantity: 4** | **Severe:** Despite detailed studies, more research is needed |
| 1 | **How and where delivered**  Delivery in a healthcare setting (n = 12) | Generally women prefer treatment at their homes. Healthcare settings were seen by women as being too busy. Implementation was aided when services acted as a “one stop shop” | Atif 2020  Bina  Boyd  Cox  kerker  Munodowafa  Ormsby | **Richness:** The data is quite rich with some quotes  **Quantity: 7** | **Moderate -**  only 7 studies, more research needed |
| 1 | Delivery in home setting (n = 5) |  | Ammerman  Beeber  Judd  Munodaw  Myors  Leger  Hadfield  Hansotte  Jones 19  Praetorius  Sorsa | **Richness:** The data is quite rich with some quotes  **Quantity: 11** | **Minor:** could be more data, but women appear to prefer home setting |
| 2 | Location |  | Gannan  Judd  Leger  Jones et al., 2014; Mollard et al., 2016; Newman et al., 2019; | **Richness:** Not particularly detailed  **Quantity: 7** | **Moderate:**  more data needed to understand good location |
| 1 | Face to face delivery (n = 4) | Provision of face to face care, as opposed to over the phone or online improved implementation. | Omahen  Doering  Shorey  Pugh | **Richness:** Not particularly detailed  **Quantity: 4** | **Moderate:**  more data needed |
| **1** | Group delivery (n = 3) | Delivery of care in a group setting, with other women with similar difficulties aided implementation. | Hadfield 2019  Masood 2015  Nakku  Dennis & Chung-Lee, 2006; Evans et al., 2020; Hadfield & Wittkowski, 2017; Jones et al., 2014; Megnin-Viggars et al., 2015; Morrell et al., 2016; Schmied et al., 2017; Scope et al., 2017; Slade et al., 2020; Tobin et al., 2018; Watson et al., 2019 | **Richness:** some quotes  **Quantity: 3** | **Minor:**  plenty of data |
| 2 | Group/peer support | Some women wanted to opportunity to talk within a peer setting |  | **Richness: most of the d**ata is rich  **Quantity: 14** |  |
| 2 | Validation | And this provided them with validation for their feelings | Jones et al., 2014; Morrell et al., 2016; Schmied et al., 2017; Slade et al., 2020 | **Richness:** The data is rich, but doesn’t tell a clear story  **Quantity: 5** | **Severe:** no clear story, little data, it is important to some women, but more research is needed |
| 1 | **Content**  Manualised therapy (n = 2) | Interventions performed according to specific guidelines sometimes helped with implementation but could feel too rigid. | Atif 2020 | **Richness:** Not detailed  **Quantity: 1** | **Severe:**  more research is needed |
| 1 | Techniques women found useful (n = 4) | Most women found personalised therapy that challenged their thoughts and beliefs (e.g. CBT approaches) useful therefore aiding implementation | Omahen  Masood  Pugh  Hadfield | **Richness:** Data quality is quite rich, with quotes  **Quantity: 4** | **Severe:**  each study says something different, not generalisable, need more research |
| 2 | Opportunity to talk | Having someone to talk to about their difficulties was a facilitator to access | Dennis & Chung-Lee, 2006; Evans et al., 2020; Hadfield & Wittkowski, 2017; Jones, 2019; Kassam, 2019; Morrell et al., 2016; Praetorius et al., 2020; Staneva et al., 2015; Megnin | **Richness:** Jones provides little detail  **Quantity: 9** | **Moderate:**  the studies are generally detailed, but there are only 9 contributing to the theme, more research please |
| 2 | Information provision | Women valued care that provided information about PNMH and options available for help and support, thus aiding access | Forde et al., 2020; Hadfield & Wittkowski, 2017; A. Jones, 2019; Megnin-Viggars et al., 2015; Morrell et al., 2016; Schmied et al., 2017; Scope et al., 2017; Slade et al., 2020; Sorsa et al., 2021  Tobin | **Richness:** Some of the studies provide detailed descriptions, others are lacking  **Quantity: 10** | **Moderate:** there are some studies that could provide more detail about *what* information would be useful |
| 1 | Technology (n = 11) | The use of technology to provide care and treatment such as reminders on screens, completing assessment questionnaires on tablets, communicating via Whatsapp, or completing therapy online aided implementation **when it was working.** | Feinberg  Fernandez  Jallo  Kim  Lind  Noonan  Pinero-leano  Shorey  Willey  Williams  Doering  +SH | **Richness:** Pineros provides extremely detailed information about this theme  **Quantity: 11 + SH Meeting** | **None/v minor:**  11 studies, some with ample detail, + real world examples from stakeholder group |
| 1 | **Organisational aspects**  Open inclusion criteria (n = 2) | Open inclusion criteria was useful for implementation . | Judd  Young | **Richness:** Quite rich data  **Quantity: 2** | **Severe:**  only 2 studies, more research needed |
| 1 | Practical support (n = 10) | The provision of support that allowed women to access care, such as providing a creche or paying for travel aided implementation | Shorey  Cox  Munodawafa  Ganan  Hadfield  Masood  Nakku  Nithianden  Ormsby | **Richness:** Data is quite rich and there is the provision of quotes  **Quantity: 9** | **minor:**  certainly appears important but not enough data to draw conclusions |
| 1 | **Perception of care**  Healthcare professional’s perception (n = 19) | Positive views of care by HCPs aided implementation | Ammerman  Atifd 2016  Atif 2020  Bina  Boyd  Chartier  Dierubg  Drozd  Feinberg  Kim  Leger  McKenzieMc  Munodowafa  Nakku  Nithian  Ormsby  Reed  Segre  Shorey  Vik  Willey | **Richness:** Extremely detailed  **Quantity: 21** | **None/very minor -**  lots of detailed data |
| 1 | Women’s perception of the care (n = 7) | Positive views of women about care aided implementation | Ammerman  Atif 2016  Atif 2020  McKenzie  Ormsby | **Richness:** Shakespeare provides particularly detailed data  **Quantity: 5** | **Moderate:**  more research needed |
| 1 | **Screening and assessment**  Wording of assessment tools (n = 3) | The clarity of the wording of the assessment tools affects implementation and access | Williams  Doering  Segre | **Richness:** The data is very detailed with quotes  **Quantity: 5** | **Moderate:**  more research needed |
| 2 | Wording of screening tools |  | Brealey et al., 2010; Hewitt et al., 2009 |  |  |
| 2 | Delivery of screening | Women did not like it when delivery was done in a tick box way with no time for discussion and no explanation beforehand  Explaining the purpose of assessment to women. | Brealey et al., 2010; Button et al., 2017; Evans et al., 2020; Hewitt et al., 2009; Smith et al., 2019  Nithianden  Shakespeare  Segre  Vik  Williams  Doering | **Richness:** Quite detailed, especially around opening dialogue  **Quantity: 11** | **Minor:**  could be more research done, but the general consensus fits across studies, quotes are provided and data is rich |
| 1 |  |  |  |  |  |
| 2 | Screening acceptability | EPDS was generally found to be acceptable to Native/Indigenous women of Canada, South Asian women living in the UK, and Black Caribbean women living in the UK However, certain questions may not elicit true feelings from Vietnamese women living in the UK because of the shame of admitting to these Q10 on the EPDS (‘the thought of harming myself has occurred to me’) was seen as problematic to Arabic, Vietnamese and Black Caribbean mothers living in the UK or USA, | Brealey et al., 2010; Button et al., 2017; Evans et al., 2020; Hewitt et al., 2009; Megnin-Viggars et al., 2015; Mollard et al., 2016; Smith et al., 2019  Doering  Shakespeare | **Richness:** Data is detailed, quotes are provided  **Quantity: 9** | **Moderate:** more research to understand why some do, and some don’t find it acceptable |
|  | Organisational |  |  |  |  |
| 1 | Continuity of carer (n = 8) | Women being able to see the same person across the care pathway was helpful in terms of implementation and access to PNMH care. | Byatt  Chartier  Higgins  Nithianden  Rowan  OMahen  Willey  Williams | **Richness:** Moderately detailed, systematic review data is more detailed  **Quantity: 17** | **None/minor -**  17 studies providing detailed clear data, plus supported by stakeholder meeting discussions so real life examples |
| 2 | Continuity of carer |  | Brealey et al., 2010; Button et al., 2017; Hadfield & Wittkowski, 2017; Megnin-Viggars et al., 2015; Slade et al., 2020; Smith et al., 2019; Tobin et al., 2018; Viveiros & Darling, 2018; Watson et al., 2019  SH |  |  |
| 1 | Organisational structure (n = 5) | A structure in which the purpose of each part of the organisation is clear and meets all of women’s needs, ensuring women don’t fall through gaps for implementation | Rowan  Higgins  Judd  Vik  Pugh | **Richness:** Data is rich from Rowan, but the rest is lacking  **Quantity: 5** | **Moderate:** more research needed |
| 1 | Referral pathways (n = 12) | Having a clear and easy process to allow for women to be referred to other services helps implement a new service. | Ammerman  Bina  Judd  Boyd  Cox  Feinberg  Nithianden  Kerker  Noonan  Willey  Rowan  Nakku | **Richness:** Data is quite rich with some representative quotes  **Quantity: 11** | **Minor:**  some more studies would be beneficial but no major gaps |
| 1 | Workflow procedures (n = 13) | Knowledge of job roles and processes within the organisation. The understanding of which tasks need to be done by whom, and how to achieve these tasks helps with implementation | Feinberg  Fernandez  Kim  Lind  Mckenzie  Segre  Munodawafa  Nithaianden  Doering  Noonan  Gannan  Pugh | **Richness:** All studies provide detailed data apart from McKenzie  **Quantity: 12** | **Minor:**  detailed data from 12 studies |
| 1 | Lack of appropriate or timely services (n = 9) | Not having anywhere to refer women on to due to lack of services or being able to refer women on to other services but the waiting time being long.  This prevented women accessing care because no service would take them, long waiting times, structural barriers, needed to be in a crisis | Boyd  Doering  Ganann  Higgins  Kerker  Leger  Lomanohaycroft  Noonan  Rowan  Williams | **Richness:** Most studies provide detailed data  **Quantity: 17** | **None/V minor:** Clear rich data with many supporting studies |
| 2 | Lack of services/overstretched |  | Bina, 2020; Button et al., 2017; Forde et al., 2020; Megnin-Viggars et al., 2015; Smith et al., 2019; Tobin et al., 2018; Viveiros & Darling, 2018 |  |  |
| 1 | Resources (n = 5) | Not having enough resources within organisations e.g. medication or support staff means cant implement a service properly | Boyd  Keker  Nakku | **Richness:** Data is moderately rich  **Quantity: 3** | **Moderate:** more research is needed |
| 1 | Service integration (n = 6) | The linking up of different services who deal with different needs, or at different time points across the perinatal period benefitted women, stopped them falling through the net – implementation + access | Ammerman  Atif  Atif 2020  Judd  Noonan  Young  Rowan | **Richness:** Data is rich, detailed  **Quantity: 13** | **No/minor:** rich data, saying the same thing from multiple sources + SH agreement so real life aspects |
| 2 | Collaboration within and across services |  | Bina, 2020; Megnin-Viggars et al., 2015; Newman et al., 2019; Smith et al., 2019; Watson et al., 2019  Forde  SH |  |  |
| 1 | Timely follow up (n = 3) | Women being followed up by healthcare professionals or other services in short time frame means you can keep women on care pathway aiding implementation | Vik  Myors  Nithianden | **Richness:** Data is not particularly rich  **Quantity: 3** | **Moderat:**  more research is needed |
| **Political factors** | | | | | |
| 1 | Funding (n = 10) | Financial resources needed to provide and implement perinatal mental healthcare. | Ammerman  Cox  Doering  Feinberg  Friendman  Gannan  Lomanon  Kim  Ormsby  Rowan | **Richness:** good detail  **Quantity: 10** | **Minor:** there is plenty of data, could be more studies as only 10 |
| 2 | Immigration status | Immigration status influenced women’s *decision to consult* and access to care due to fears of being deported | Bina, 2020; Giscombe et al., 2020; Hansotte et al., 2017; Kassam, 2019; Schmied et al., 2017  Ganann | **Richness:** Data is very rich  **Quantity: 6** | **Moderate:**  more research is needed into this |
| 2 | Economic status | This influenced womens decision to consult, access to care (because can’t afford it) but also their experience of care because cant find wellbeing when you cant fulfil basic needs  Costs of healthcare related to the above point. If you cant afford it, you don’t consult | Schmied  Tobin  Lucas  Kassam  Bina  Hansotte  Viveiros  Jones | **Richness:** The data is rich, there are quotes, some quite harrowing  **Quantity: 8** | **Moderate:**  more research is needed into this |
| 2 | Healthcare costs |  |  |  |  |
|  | Societal factors |  |  |  |  |
| 1 | Stigma | Stigma prevented implementation and access to care. it was pervasive. | Atif 2016  Atif 2020  Beeber  Bina  Boyd  Chartier  Cox  Feinberg  Higgins  Keker  MCcAULEY  McKenzie  Munodawafa  Myors  Nakku  Nithianden  Noonan  Shakesperare  Vik  Williams  Young | **Richness:** All very detailed  **Quantity: 43** | **None -**  a lot of data |
| 2 | Stigma |  | Bina, 2020; Button et al., 2017; Dennis & Chung-Lee, 2006; Forde et al., 2020; Giscombe et al., 2020; Hadfield & Wittkowski, 2017; Hansotte et al., 2017; Hewitt et al., 2009; Holopainen & Hakulinen, 2019; Jones, 2019; Kassam, 2019; Lucas et al., 2019; Megnin-Viggars et al., 2015; Morrell et al., 2016; Nilaweera et al., 2014; Mollard 17; Smith et al., 2019; Sorsa et al., 2021; Staneva et al., 2015; Tobin et al., 2018; Viveiros & Darling, 2018; Watson et al., 2019 |  |  |
| 1 | Culture (n = 13) | The beliefs and behaviours of a particular group of people or a society.  Certain cultural beliefs about mental illness (i.e. it isn’t real, or its caused by spirits) means people are less likely to consult. Culture and stigma interlink. You have to be strong, have to fulfil gender roles, punishment for being black (more likely to be labelled) | Atif 2016  Bina  Boyd  Friedman  Ganann  Higgins  Masood  McCauley  Naku  Nithanden  Noonan  Segre  Shorey | **Richness:** The data is very rich, there is a lot of detail related to this  **Quantity: 32** | **None -**  a lot of data |
| 2 | Culture |  | Bina, 2020; Brealey et al., 2010; Button et al., 2017; Dennis & Chung-Lee, 2006; Giscombe et al., 2020; Hansotte et al., 2017; Hewitt et al., 2009; Jones, 2019; Kassam, 2019; Megnin-Viggars et al., 2015; Nilaweera et al., 2014; Praetorius et al., 2020; Schmied et al., 2017; Smith et al., 2019; Sorsa et al., 2021; Staneva et al., 2015; Tobin et al., 2018; Viveiros & Darling, 2018; Watson et al., 2019; Wittkowski et al., 2014 |  |  |
| 2 | Maternal norms | The **maternal norm** for women to show they are strong, that they can cope and be a good mother (n = 19), prevented women from *deciding to consult, disclosing, accessing care* and *women’s experience of care* | Bina, 2020; Brealey et al., 2010; Button et al., 2017; Dennis & Chung-Lee, 2006; Forde et al., 2020; Hadfield & Wittkowski, 2017; Hansotte et al., 2017; Hewitt et al., 2009; Holopainen & Hakulinen, 2019; Jones et al., 2014; Lucas et al., 2019; Mollard et al., 2016; Morrell et al., 2016; Newman et al., 2019; Schmied et al., 2017; Slade et al., 2020; Smith et al., 2019; Sorsa et al., 2021; Staneva et al., 2015; Viveiros & Darling, 2018 | **Richness:** The data was rich, with lots of quotes  **Quantity: 22** | **None/v minor:** a lot of consistent data |

# *Supplementary Materials* *7:* Geographical distribution of research

| **Review** | **UK** | **HIC/Western** | **LMIC/Eastern** | **Don’t know** |
| --- | --- | --- | --- | --- |
| 1 | *Hadfield et al., 2019; Masood et al., 2015; McKenzie-McHarg et al., 2014; O’Mahen & Flynn, 2008; Rowan et al., 2010; Shakespeare et al., 2003; Williams et al., 2016* | **Australia:** *Judd et al., 2011; Myors et al., 2015; Nithianandan et al., 2016; Ormsby et al., 2018; Reed et al., 2014; Willey et al., 2018*  **Canada:** *Chartier et al., 2015; Leger et al., 2015; Pugh et al., 2015*  **Ireland:** *Higgins et al., 2018; Noonan et al., 2018*  **Norway:** *Drozd et al., 2018; Vik et al., 2009*  **USA:** *Ammerman et al., 2014; Beeber et al., 2009; Boyd et al., 2011; Byatt et al., 2013; Cox et al., 2017; Doering et al., 2017; Feinberg et al., 2006; Fernandez y Garcia et al., 2011; Friedman et al., 2010; Ganann et al., 2019; Jallo et al., 2015; Kerker et al., 2018; Kim et al., 2009; Lind et al., 2017; Lomonaco-Haycraft et al., 2018; Pineros-Leano et al., 2015; Segre et al., 2014; Young et al., 2019* | **Ghana:** *Munodawafa et al., 2017*  **Israel:** *Bina et al., 2018*  **Pakistan:** *Atif et al., 2016, 2019*  **Peru:** *Eappen et al., 2018*  **Singapore:** *Shorey & Ng, 2019*  **South Africa:** *McCauley et al., 2019*  **Uganda:** *Nakku et al., 2016* |  |
| 2 | Brealey et al., 2010 – 53%  Button et al., 2017 – 100%  Forde et al., 2020 – 66%  Hadfield & Wittkowski, 2017 – 53%  Hewitt et al., 2009– 53%  Sambrook Smith et al., 2019– 100%  Slade et al., 2020– 53%  Viveiros & Darling, 2018 – 71%  Watson et al., 2019 – 100% | Bina, 2020 – 83%  Evans et al., 2020 – 100%  Giscombe et al., 2020– 88%  Hansotte et al., 2017 – 100%  Holopainen & Hakulinen, 2019 – 77%  Jones et al., 2014 – 100%  Kassam, 2019– 88%  Lucas et al., 2019 – 100%  Mollard et al., 2016 – 100%  Morrell et al., 2016– 92%  Newman et al., 2019– 100%  Nilaweera et al., 2014 – 100%  Praetorius et al., 2020– 75%  Randall & Briscoe, 2018– 100%  Schmied et al., 2017 – 100%  Scope et al., 2017 – 95%  Sorsa et al., 2021 – 100%  Staneva et al., 2015– 88%  Tobin et al., 2018– 100% | Wittkowski et al., 2014 – 100% (sub-Saharan Africa) | Dennis & Chung-Lee, 2006; A. Jones, 2019; Megnin-Viggars et al., 2015 |

# *Supplementary Materials 8.* GRADE-CERQual Relevance rating table

| Theme | Summary of review finding | Studies contributing to finding | Assessment of relevance based on country of study | | | | Overall rating |
| --- | --- | --- | --- | --- | --- | --- | --- |
|  |  |  | Direct relevanceUK | Partial relevance Western Country | Indirect relevanceLMIC Eastern | Unclear relevance |  |
| Additional personal difficulties | Difficulties that women may have that are not related to perinatal mental illness, such as poverty can prevent successful service implementation | Ganann 2019 Rowan 2010 Kerker 2018 Munodawafa 2017 Williams 2016 Atif 2016 Atif 2020 Boyd 2011 Cox 2017 Nakku 2016 | Rowan Williams | Ganann Kerker Boyd Cox | Munodawafa Atif Atif 20 Nakku |  | **Minor concerns**  - majority have direct and partial relevance to the theme, the healthcare service in question isn’t relevant here because it is directly related to women |
|  |  |  | NHS | Universal govt funded HC | Universal insurance | Non Universal insurance |  |
|  |  |  | Rowan Williams | Munodawafa |  | Atif 16 Atif 20 Nakku Gannan Kerker Boyd Cox |  |
| Family Family and friends | Negative beliefs from women’s family and friends can prevent successful implementation, help-seeking and women’s access to services | Atif 2016 Atif 2020 Boyd 2011 Doering 2017 Gannan 2019 Higgins 2018 Masood 2015 Nakku 2016 Nithianden Noonan o mahen Pineros Leano Williams Vik 2009 Young 2019 Bina, 2020; Button et al., 2017; Dennis & Chung-Lee, 2006; Forde et al., 2020; Hadfield & Wittkowski, 2017; Hansotte et al., 2017; Holopainen & Hakulinen, 2019; Jones, 2019; Lucas et al., 2019; Nilaweera et al., 2014; Schmied et al., 2017; Smith et al., 2019; Sorsa et al., 2021; Viveiros & Darling, 2018; Watson et al., 2019 | Direct relevance UK | Partial relevance Western Country | Indirect relevance LMIC Eastern | Unclear relevance | **Minor concerns**  - majority have direct and partial relevance to the theme, the healthcare service in question isn’t relevant here because it is directly related to women |
|  |  |  | Masood O’Mahen Williams Bina Button Forde Hadfield Smith Viveiros Watson | Boyd Doering Ganann Higgins Nithianden Noonan Pineros-Leano Vik Young Hansotte Holopainen Lucas Nilaweera Schmied Sorsa | Atif 16 Atif 20 Nakku | Dennis Jones 19 |  |
|  |  |  |  |  |  |  |  |
|  |  |  | NHS | Universal govt funded HC | Universal insurance Don’t know | Non Universal insurance |  |
|  |  |  | Masood O’Mahen Williams Button Forde Hadfield Smith Viveiros Watson | Higgins Nithianden Noonan Vik Nilaweera Schmied Sorsa | Bina Dennis Holopainen Jones 2019 | Atif Atif Boyd Doering Gannan Nakku Pineros-leano Young Hansotte Lucas |  |
| Social isolation | Social isolation can exacerbate women’s symptoms, but can also be exacerbated by social isolation. This can affect help seeking and women’s experiences of care | Kassam Bina Jones 14 Jones 19 Viveiros Giscombe Watson Tobin | Direct relevance UK | Partial relevance Western Country | Indirect relevance LMIC Eastern | Unclear relevance | **Minor concerns**  - majority have direct and partial relevance to the theme, the healthcare service in question isn’t relevant here because it is directly related to women |
|  |  |  | Viveiros Watson | Kassam Bina Jones 14 Giscombe Tobin |  | Jones 19 |  |
|  |  |  | NHS | Universal govt funded HC | Universal insurance Don’t know | Non Universal insurance |  |
|  |  |  | Viveiros Watson | Kassam Jones 14 Giscombe Tobin | Bina Jones 19 |  |  |
| Beliefs about the causes of mental illness | When women believe their symptoms are caused by a biological predisposition, or that their symptoms are caused by spiritual, physical or external factors, or are a normal response to motherhood this hinders implementation and prevents women help seeking and accessing services. | O'Mahen 2015 Feinberg 2006 | Direct relevance UK | Partial relevance Western Country | Indirect relevance LMIC Eastern | Unclear relevance | **Minor -**  direct and partially relevant |
|  |  |  | O’Mahen | Feinberg |  |  |  |
|  |  |  | NHS | Universal govt funded HC | Universal insurance Don’t know | Non Universal insurance |  |
|  |  |  | O’Mahen |  |  | Feinberg |  |
| Spiritual |  | Schmied et al., 2017; Wittkowski et al., 2014 | Direct relevance UK | Partial relevance Western Country | Indirect relevance LMIC Eastern | Unclear relevance | **Moderate -**  no direct relevance, different health services |
|  |  |  |  | Schmied | Witkowski |  |  |
|  |  |  | NHS | Universal govt funded HC | Universal insurance Don’t know | Non Universal insurance |  |
|  |  |  |  | Schmied |  | Witkowski |  |
| External |  | Bina, 2020; Button et al., 2017; Dennis & Chung-Lee, 2006; Lucas et al., 2019; Schmied et al., 2017; Staneva et al., 2015; Tobin et al., 2018; Watson et al., 2019 | Direct relevance UK | Partial relevance Western Country | Indirect relevance LMIC Eastern | Unclear relevance | **Minor concerns**  - majority have direct and partial relevance to the theme, the healthcare service in question isn’t relevant here because it is directly related to women |
|  |  |  | Button Watson | Bina Lucas Schmied Staneva Tobin |  | Dennis |  |
|  |  |  | NHS | Universal govt funded HC | Universal insurance Don’t know | Non Universal insurance |  |
|  |  |  | Button Watson | Schmied Staneva Tobin | Bina | Lucas |  |
| Physical |  | Bina, 2020; Button et al., 2017; Dennis & Chung-Lee, 2006; Forde et al., 2020; Jones et al., 2014; Newman et al., 2019; Schmied et al., 2017; Smith et al., 2019; Staneva et al., 2015; Watson et al., 2019 | Direct relevance UK | Partial relevance Western Country | Indirect relevance LMIC Eastern | Unclear relevance | **Minor concerns**  - majority have direct and partial relevance to the theme, the healthcare service in question isn’t relevant here because it is directly related to women |
|  |  |  | Button Forde Smith Watson | Bina Jones 14 Newman Schmied Staneva |  | Dennis |  |
|  |  |  | NHS | Universal govt funded HC | Universal insurance Don’t know | Non Universal insurance |  |
|  |  |  | Button Forde Smith Watson | Jones 14 Newman Schmied Staneva | Bina Dennis |  |  |
| Normal |  | Dennis & Chung-Lee, 2006; Giscombe et al., 2020; Jones et al., 2014; Schmied et al., 2017; Slade et al., 2020; Smith et al., 2019; Sorsa et al., 2021 | Direct relevance UK | Partial relevance Western Country | Indirect relevance LMIC Eastern | Unclear relevance | **Minor concerns**  - majority have direct and partial relevance to the theme, the healthcare service in question isn’t relevant here because it is directly related to women |
|  |  |  | Slade Smith | Giscombe Jones 14 Schmied Sorsa |  | Dennis |  |
|  |  |  | NHS | Universal govt funded HC | Universal insurance Don’t know | Non Universal insurance |  |
|  |  |  | Slade Smith | Giscombe Jones Schmied Sorsa |  |  |  |
| Dealing with symptoms - ignore | Depending on how women view their symptoms will affect how they deal with their symptoms. When women choose to ignore their difficulties, minimise their symptoms or seek spiritual guidance this is a barrier to help-seeking and access | Bina, 2020; Hadfield & Wittkowski, 2017; Jones, 2019; Newman et al., 2019; Schmied et al., 2017; Slade et al., 2020 | Direct relevance UK | Partial relevance Western Country | Indirect relevance LMIC Eastern | Unclear relevance | **Minor concerns**  - majority have direct and partial relevance to the theme, the healthcare service in question isn’t relevant here because it is directly related to women |
|  |  |  | Hadfield Slade | Bina Newman Schmied |  | Jones 19 |  |
|  |  |  | NHS | Universal govt funded HC | Universal insurance Don’t know | Non Universal insurance |  |
|  |  |  | Hadfield Slade | Newman Schmied | Bina Jones 19 |  |  |
| seeking spiritual guidance |  | Hansotte et al., 2017; Kassam, 2019; Schmied et al., 2017; Watson et al., 2019 | Direct relevance UK | Partial relevance Western Country | Indirect relevance LMIC Eastern | Unclear relevance | **Minor concerns**  - majority have direct and partial relevance to the theme |
|  |  |  | Watson | Hansotte Kassam Schmied |  |  |  |
|  |  |  | NHS | Universal govt funded HC | Universal insurance Don’t know | Non Universal insurance |  |
|  |  |  | Watson | Kassam Schmied |  | Hansotte |  |
| Symptom minimisation |  | Bina, 2020; Dennis & Chung-Lee, 2006; Forde et al., 2020; Hewitt et al., 2009; Holopainen & Hakulinen, 2019; Jones et al., 2014; Kassam, 2019; Lucas et al., 2019; Megnin-Viggars et al., 2015; Schmied et al., 2017; Staneva et al., 2015; Watson et al., 2019 | Direct relevance UK | Partial relevance Western Country | Indirect relevance LMIC Eastern | Unclear relevance | **Minor concerns**  - majority have direct and partial relevance to the theme |
|  |  |  | Forde Hewitt Watson | Bina Holopainen Jones 14 Kassam Lucas Schmied Staneva |  | Dennis Megnin |  |
|  |  |  | NHS | Universal govt funded HC | Universal insurance Don’t know | Non Universal insurance |  |
|  |  |  | Forde Hewitt Watson | Jones 14 Schmied Staneva | Bina Holopainen |  |  |
| Recognising something is wrong | Women have to recognise something is wrong before they seek help | Bina, 2020; Button et al., 2017; Forde et al., 2020; Hadfield & Wittkowski, 2017; Hansotte et al., 2017; Slade et al., 2020; Sorsa et al., 2021; Staneva et al., 2015; Viveiros & Darling, 2018 | Direct relevance UK | Partial relevance Western Country | Indirect relevance LMIC Eastern | Unclear relevance | **Minor concerns**  - majority have direct and partial relevance to the theme, the healthcare service in question isn’t relevant here because it is directly related to women |
|  |  |  | Button Forde Hadfield Slade Viveiros | Bina Hansotte Sorsa Staneva |  |  |  |
|  |  |  | NHS | Universal govt funded HC | Universal insurance **Don’t know** | Non Universal insurance |  |
|  |  |  | Button Forde Hadfield Slade Viveiros | Sorsa Staneva | Bina | Hansotte |  |
| Not knowing where to go | Not having enough information to know where to go is a barrier to help seeking | Bina Dennis Hansotte Megnin-viggars Schmied Smith Sorsa Tobin | Direct relevance UK | Partial relevance Western Country | Indirect relevance LMIC Eastern | Unclear relevance | **Moderate –** only one review reflects UK and NHS services, perhaps more relevant in other countries? |
|  |  |  | Smith | Bina Hansotte Schmied Sorsa Tobin |  | Dennis Megnin |  |
|  |  |  | NHS | Universal govt funded HC | Universal insurance **Don’t know** | Non Universal insurance |  |
|  |  |  | Smith | Schmied Sorsa Tobin | Bina | Hansotte |  |
| Beliefs about health services – medication only | Beliefs about health services are also a barrier to helpseeking and access, such as believing services only offer medication, are stretched, HCPs wont care or that they not sure if the HCP they are talking with are equipped to deal with their symptoms, | Bina, 2020; Button et al., 2017; Jones, 2019; Megnin-Viggars et al., 2015; Nilaweera et al., 2014; Sorsa et al., 2021; Tobin et al., 2018 | Direct relevance UK | Partial relevance Western Country | Indirect relevance LMIC Eastern | Unclear relevance | **Moderate –** only one review reflects UK and NHS services, perhaps more relevant in other countries? |
|  |  |  | Button | Bina Nilaweera Sorsa Tobin |  | Jones Megnin |  |
|  |  |  | NHS | Universal govt funded HC | Universal insurance **Don’t know** | Non Universal insurance |  |
|  |  |  | Button | Nilaweera Sorsa Tobin | Bina |  |  |
| Beliefs about health services - stretched |  | Hadfield & Wittkowski, 2017 | Direct relevance UK | Partial relevance Western Country | Indirect relevance LMIC Eastern | Unclear relevance | **No/Minor**  - only one study though |
|  |  |  | Hadfield |  |  |  |  |
|  |  |  | NHS | Universal govt funded HC | Universal insurance **Don’t know** | Non Universal insurance |  |
|  |  |  | Hadfield |  |  |  |  |
| Beliefs about HCPs – what is their role |  | Brealey et al., 2010; Button et al., 2017; Dennis & Chung-Lee, 2006; Hadfield & Wittkowski, 2017; Hewitt et al., 2009; Megnin-Viggars et al., 2015; Mollard et al., 2016; Morrell et al., 2016; Nilaweera et al., 2014; Schmied et al., 2017; Scope et al., 2017; Smith et al., 2019 | Direct relevance UK | Partial relevance Western Country | Indirect relevance LMIC Eastern | Unclear relevance | **Minor concerns**  - majority have direct and partial relevance to the theme, the healthcare service in question isn’t relevant here because it is directly related to women |
|  |  |  | Brealey Button Hadfield Hewitt Smith | Mollard Morrell Nilaweera Schmied Scope |  | Dennis Megnin |  |
|  |  |  | NHS | Universal govt funded HC | Universal insurance **Don’t know** | Non Universal insurance |  |
|  |  |  | Brealey Button Hadfield Hewitt Smith | Morrell Nilaweera Schmied Scope | Dennis Megnin | Mollard |  |
| Beliefs about HCPs – they won’t be interested |  | Bina, 2020; Hadfield & Wittkowski, 2017 | Direct relevance UK | Partial relevance Western Country | Indirect relevance LMIC Eastern | Unclear relevance | **Minor -**  but only 2 studies |
|  |  |  | Hadfield | Bina |  |  |  |
|  |  |  | NHS | Universal govt funded HC | Universal insurance **Don’t know** | Non Universal insurance |  |
|  |  |  | Hadfield |  | Bina |  |  |
| Logistics – childcare | Logistical issues effect implementation, help seeking and access to care. These logistical issues include, childcare, travel, timing of appointments | Bina, 2020; Button et al., 2017; Dennis & Chung-Lee, 2006; Hansotte et al., 2017; Jones 19 Morrell et al., 2016; Newman et al., 2019; Scope et al., 2017; Smith et al., 2019; Tobin et al., 2018; Watson et al., 2019 Boyd Cox Doering Friedman | Direct relevance UK | Partial relevance Western Country | Indirect relevance LMIC Eastern | Unclear relevance | **Moderate -**  seems to be an issue in most cultures and health services |
|  |  |  | Button Smith Watson | Bina Hansotte Morrell Newman Scope Tobin Boyd Cox Doering Friedman |  | Dennis Jones 19 |  |
|  |  |  | NHS | Universal govt funded HC | Universal insurance **Don’t know** | Non Universal insurance |  |
|  |  |  | Button Smith Watson | Morrell Newman Schmied Scope Tobin | Bina Dennis Jones 19 | Hansotte Boyd Cox Doering Friedman |  |
| Logistics – timing |  | Bina, 2020; Dennis & Chung-Lee, 2006; Newman et al., 2019; Scope et al., 2017; Watson et al., 2019 Atif 16 Pugh Atif 20 Chartier Cox Friedman | Direct relevance UK | Partial relevance Western Country | Indirect relevance LMIC Eastern | Unclear relevance | **Moderate –** less representation in UK services and country, seems to affect non universal insurance HC more |
|  |  |  | Watson | Bina Newman Scope Pugh Chartier Cox Friedman | Atif Atif | Dennis |  |
|  |  |  | NHS | Universal govt funded HC | Universal insurance **Don’t know** | Non Universal insurance |  |
|  |  |  | Watson | Newman Scope Pugh Chartier | Bina Dennis | Atif Atif 20 Cox Friedman |  |
| Logistics – location/travel |  | Bina Hansotte Jones 19 Mollard Morrell Newman Sorsa Tobin Watson Young Eappen Massood Doering | Direct relevance UK | Partial relevance Western Country | Indirect relevance LMIC Eastern | Unclear relevance | **Minor -**  seems to be relevant in NHS, universally funded and insurance services as well as eastern and wester |
|  |  |  | Watson Masood | Bina Hansotte Mollard Morrell Newman Sorsa Tobin Young Doering | Eappen | Jones 19 |  |
|  |  |  | NHS | Universal govt funded HC | Universal insurance **Don’t know** | Non Universal insurance |  |
|  |  |  | Watson Masood | Morrell Newman Sorsa Tobin | Bina Eappen | Hansotte Mollard Young Doering |  |
| Symptoms of psychological difficulty | Psychological factors such as fear of judgement, feeling psychologically ready to take part in care, symptoms of psychological difficulties, and fear of social services involvement prevent implementation, help seeking and access to PNMH care | Friedman 2011 Boyd 2011 Hadfield 2019 | Direct relevance UK | Partial relevance Western Country | Indirect relevance LMIC Eastern | Unclear relevance | **Minor -**  but only 3 studies |
|  |  |  | Hadfield | Friedman Boyd |  |  |  |
|  |  |  | NHS | Universal govt funded HC | Universal insurance **Don’t know** | Non Universal insurance |  |
|  |  |  | Hadfield |  |  | Friedman Boyd |  |
| Fear of being judged as a bad mum |  | Bina, 2020; Brealey et al., 2010; Button et al., 2017; Forde et al., 2020; Jones et al., 2014; Lucas et al., 2019; Schmied et al., 2017; Slade et al., 2020; Sorsa et al., 2021; Viveiros & Darling, 2018; Watson et al., 2019 | Direct relevance UK | Partial relevance Western Country | Indirect relevance LMIC Eastern | Unclear relevance | **None/V minor –** most are uk based studies |
|  |  |  | Brealey Button Forde Slade Viveiros Watson | Bina Jones 14 Lucas Schmied Sorsa |  |  |  |
|  |  |  | NHS | Universal govt funded HC | Universal insurance **Don’t know** | Non Universal insurance |  |
|  |  |  | Brealey Button Forde Slade Viveiros Watson | Jones Schmied Sorsa | Bina | Lucas |  |
| Social services |  | Bina, 2020; Brealey et al., 2010; Button et al., 2017; Dennis & Chung-Lee, 2006; Evans et al., 2020; Forde et al., 2020; Hadfield & Wittkowski, 2017; Hewitt et al., 2009; Jones, 2019; Megnin-Viggars et al., 2015; Newman et al., 2019; Tobin et al., 2018; Watson et al., 2019 Stakeholder | Direct relevance UK | Partial relevance Western Country | Indirect relevance LMIC Eastern | Unclear relevance | **None/V minor –** all saying the same thing and brought up at stakeholder meetings |
|  |  |  | Brealey Buton Forde Hadfield Hewitt Watson +SH | Bina Evans Newman Tobin |  | Dennis Jones 19 Megnin |  |
|  |  |  | NHS | Universal govt funded HC | Universal insurance **Don’t know** | Non Universal insurance |  |
|  |  |  | Brealey Buton Forde Hadfield Hewitt Watson +SH | Evans Newman Tobin | Bina Dennis Jones 19 Megnin |  |  |
| Psychological readiness |  | Leger 2005 Chartier 2015 | Direct relevance UK | Partial relevance Western Country | Indirect relevance LMIC Eastern | Unclear relevance | **Moderate –** not directly related to UK/NHS |
|  |  |  |  | Leger Chartier |  |  |  |
|  |  |  | NHS | Universal govt funded HC | Universal insurance **Don’t know** | Non Universal insurance |  |
|  |  |  |  | Leger Chartier |  |  |  |
| Ethnicity | Demographic factors such as age (older women are more likely to seek help), ethnicity (white women are more likely to seek help), and previous experiences with mental health services (those with previous diagnoses and positive experiences of MHS are more likely to seek help) impacted helpseeking and access to PNMH Care | Bina, 2020; Dennis & Chung-Lee, 2006; Hansotte et al., 2017; Watson et al., 2019 | Direct relevance UK | Partial relevance Western Country | Indirect relevance LMIC Eastern | Unclear relevance | **Moderate -**  not a very clear pattern, could be relevant, need more research |
|  |  |  | Watson | Bina Hansotte |  | Dennis |  |
|  |  |  | NHS | Universal govt funded HC | Universal insurance **Don’t know** | Non Universal insurance |  |
|  |  |  | Watson |  | Bina | Hansotte |  |
| Age |  | Bina, 2020; Hansotte et al., 2017 | Direct relevance UK | Partial relevance Western Country | Indirect relevance LMIC Eastern | Unclear relevance | **Severe –** No direct relevance |
|  |  |  |  | Bina Hansotte |  |  |  |
|  |  |  | NHS | Universal govt funded HC | Universal insurance **Don’t know** | Non Universal insurance |  |
|  |  |  |  |  | Bina | Hansotte |  |
| Previous experiences |  | O’mahen Button et al., 2017; Evans et al., 2020; Hadfield & Wittkowski, 2017; Hansotte et al., 2017; Jones, 2019; Sorsa et al., 2021; Watson et al., 2019 | Direct relevance UK | Partial relevance Western Country | Indirect relevance LMIC Eastern | Unclear relevance | **Minor -**  seems to be relevant in NHS, universally funded and insurance services as well as eastern and wester |
|  |  |  | O MAHEN Button Hadfield Watson | Evans Hansotte Sorsa |  | Jones 19 |  |
|  |  |  |  |  |  |  |  |
|  |  |  | NHS | Universal govt funded HC | Universal insurance **Don’t know** | Non Universal insurance |  |
|  |  |  | O MAHEN Button Hadfield Watson | Evans Sorsa | Jones 19 | Hansotte |  |
| Previous diagnoses |  | Bina, 2020; | Direct relevance UK | Partial relevance Western Country | Indirect relevance LMIC Eastern | Unclear relevance | **Severe –** no direct relevance |
|  |  |  |  | Bina |  |  |  |
|  |  |  | NHS | Universal govt funded HC | Universal insurance **Don’t know** | Non Universal insurance |  |
|  |  |  |  |  | Bina |  |  |
| HCPS |  |  |  |  |  |  |  |
| Characteristics of healthcare professionals (n = 19) Characteristics of HCPs | The way healthcare professionals acted towards women. For example, open, non-judgmental, willing to listen, motivated and interested healthcare professionals were valued by women, aided implementation. Trustworthy, readily available, helpful, interested, nonjudgemental, understanding, caring, positive, empathetic, warm, above and beyond were all facilitators to PNMH access. | Atif Boyd Chartier Doering Feinberg Kerker Gannan Higgins Judd Kim Leger Masood Munodawaf Myors Nithian Noonan Pugh Shorey Willioams Atif 2020 Stakeholders Brealey et al., 2010; Button et al., 2017; Dennis & Chung-Lee, 2006; Forde et al., 2020; Hadfield & Wittkowski, 2017; Hewitt et al., 2009; Megnin-Viggars et al., 2015; Morrell et al., 2016; Newman et al., 2019; Schmied et al., 2017; Slade et al., 2020; Staneva et al., 2015; Viveiros & Darling, 2018 | Direct relevance UK | Partial relevance Western Country | Indirect relevance LMIC Eastern | Unclear relevance | **None-**  seems to be relevant in NHS, universally funded and insurance services as well as eastern and wester and have relevance from SH meeting |
|  |  |  | Masood Williams Brealey Button Forde Hadfield Hewitt Slade Viveiros Stakeholders | Boyd Chartier Doering Feinberg Kerke Ganann Higgins Judd Kim Leger Myors Nithianden Noonan Pugh Morrell Newman Staneva | Atif Atif 20 Munadowafa Shorey | Dennis Megnin |  |
|  |  |  |  |  |  |  |  |
|  |  |  | NHS | Universal govt funded HC | Universal insurance **Don’t know** | Non Universal insurance |  |
|  |  |  | Masood Williams Brealey Button Forde Hadfield Hewitt Slade Viveiros | Chartier Higgins Judd Leger Myors Nithianden Noonan Pugh Munadowafa Shorey Morrell Newman Staneva | **Dennis Megnin** | Atif Atif 20 Boyd Doering Feinberg Kerke Ganann Kim rs |  |
| HCP being dismissive or normalising symptoms | This prevented women from accessing care | Button et al., 2017; Dennis & Chung-Lee, 2006; Forde et al., 2020; Hadfield & Wittkowski, 2017; Hansotte et al., 2017; Megnin-Viggars et al., 2015; Newman et al., 2019; Sorsa et al., 2021; Watson et al., 2019 Stakeholder | Direct relevance UK | Partial relevance Western Country | Indirect relevance LMIC Eastern | Unclear relevance | **None -**  most UK NHS based plus stakeholder support |
|  |  |  | Button Forde Hadfield Watson sh | Hansotte Newman Sorsa |  | Dennis Megnin |  |
|  |  |  | NHS | Universal govt funded HC | Universal insurance **Don’t know** | Non Universal insurance |  |
|  |  |  | Button Forde Hadfield Watson SH | Newman Sorsa | **Dennis Megnin** | Hansotte |  |
| The way the HCP delivers the care | HCPs focussing on the infant, not asking about PNMH or asking in a tick-box way, not treating women as individuals and not being culturally sentitive were barriers to care | Button et al., 2017; Forde et al., 2020; Kassam, 2019; Megnin-Viggars et al., 2015; Nilaweera et al., 2014; Schmied et al., 2017; Slade et al., 2020; Smith et al., 2019; Sorsa et al., 2021 | Direct relevance UK | Partial relevance Western Country | Indirect relevance LMIC Eastern | Unclear relevance | **Minor -**  most directly relevant or partially relevant |
|  |  |  | Button Forde Slade Smith | Kassam Nilaweera Schmied Sorsa |  | Megnin |  |
|  |  |  | NHS | Universal govt funded HC | Universal insurance **Don’t know** | Non Universal insurance |  |
|  |  |  | Button Forde Slade Smith | Kassam Nilaweera Schmied Sorsa | **Megnin** |  |  |
| Collaborative working (n = 18) | Healthcare professionals working together to improve care for women. | Ammerman Boyds Byatt Cox Eappen Feinberg Ganann Higgins Judd Kerker Lind Lomonaco Munodawafa Myors Nithianden Noonan Rowan Willey Stakeholder | Direct relevance UK | Partial relevance Western Country | Indirect relevance LMIC Eastern | Unclear relevance | **Minor -**  although most are partially relevant, rown fits and we have real life examples from the stakeholder meetings |
|  |  |  | SH Rowan | Ammermna Boyd Byatt Chartier Cox Ganann Higgins Judd Feinberg Myors Nithianden Noonan Willey Kerker | Eappen Munodawafa |  |  |
|  |  |  | NHS | Universal govt funded HC | Universal insurance **Don’t know** | Non Universal insurance |  |
|  |  |  | SH Rowan | Chartier Higgins Judd Munodawafa Noonan Willey |  | Ammerman Boyd Byatt Cox Gannan Feinberg Kerker Lind Lomanoco |  |
| Communication between healthcare professionals (n = 10) | Clear communication amongst healthcare professionals. | Bina Byatt Cox Eappen Lind Mckenzie Nithiandan Orsmby Riwan Segre Stakeholders | Direct relevance UK | Partial relevance Western Country | Indirect relevance LMIC Eastern | Unclear relevance | **None -**  although most are partially relevant, rown fits and we have real life examples from the stakeholder meetings |
|  |  |  | McKenzie Rowan SH | Byatt Cox Lind Nithianden Ormsby Segre | Bina Eappen |  |  |
|  |  |  | NHS | Universal govt funded HC | Universal insurance **Don’t know** | Non Universal insurance |  |
|  |  |  | McKenzie Rowan SH | Nithianden Ormsby | Bina Eappen | Byatt Cox Lind Segre |  |
| Confidence of healthcare providers (n = 12) | Healthcare professional’s belief in their ability to provide the care being offered to women. | Atif 2020 Beeber Bina Cox Fernandez Higgins Munodawara Nithianden Ormsby Reed Vik Stakeholders | Direct relevance UK | Partial relevance Western Country | Indirect relevance LMIC Eastern | Unclear relevance | **Minor-**  none directly relevant, but some partially relevant stakeholders suggested training so HCP don’t have fear if someone mentions PNMI |
|  |  |  | **SH** | Beeber Cox Fernandez Higgins Nithianden Ormsby Reed Vik | Atif Bina Munodowafa |  |  |
|  |  |  | NHS | Universal govt funded HC | Universal insurance **Don’t know** | Non Universal insurance |  |
|  |  |  |  | Munodawafa Higgins Niathianden Ormsby Reed Vik | Bina | Atif Beeber Cox Fernandez |  |
| Dedicated person to act as women’s advocate (n = 7) | Someone who can explain the process of the service and who is there for women throughout the care pathway. | Lomonoco haycroft Chartier Kim Ganann Nithian Rowan Willey Bina (SR) | Direct relevance UK | Partial relevance Western Country | Indirect relevance LMIC Eastern | Unclear relevance | **Minor -**  only one directly linked, but linked to universal govt funded health services and services in western countries |
|  |  |  | Rowan | Lomanco Chartier Kim Ganann Nithianden Willey Bina (sr) |  |  |  |
|  |  |  | NHS | Universal govt funded HC | Universal insurance **Don’t know** | Non Universal insurance |  |
|  |  |  | Rowan | Chartier Nithianden Willey |  | Lomanoco Kim Ganann |  |
| Heavy workload (n = 20) | A heavy workload or not enough time to provide the care being offered by the service was a barrier to implementation and HCP appearing to busy to women was a barrier to PNMH access. | Ammerman Bina Drozd Eappen Feinberg Fernandez Gannan Higgins Kerker Kim McCauley McKenzie Nakku Nithianden Noonan Rowan Shorey Vik Willey Stakeholders | Direct relevance UK | Partial relevance Western Country | Indirect relevance LMIC Eastern | Unclear relevance | **None -**  universal + support from SHs |
|  |  |  | McKenzie Rowan | Ammerman Drozd Feinberg Fernandez Ganann Higgins Kerker Kim Nithianden Vik Willey | Bina McCauley Nakku Shorey Eappen |  |  |
|  |  |  | NHS | Universal govt funded HC | Universal insurance **Don’t know** | Non Universal insurance |  |
|  |  |  | McKenzie Rowan | Drozd Higgins Nithianden Noonan Shorey Vik Wiley | Bina Eappen | Ammerman Feinberg Fernande Ganann Kerker Kim McCauley Nakku |  |
| HCP appearing too busy from women’s perspective |  | Bina, 2020; Button et al., 2017; Dennis & Chung-Lee, 2006; Hewitt et al., 2009; Megnin-Viggars et al., 2015; Slade et al., 2020; Viveiros & Darling, 2018; Watson et al., 2019 | Direct relevance UK | Partial relevance Western Country | Indirect relevance LMIC Eastern | Unclear relevance | **None**  - all the ones where it is clear the ocation all suggest this |
|  |  |  | Button Hewitt Slade Viveiros Watson |  |  | Bina Dennis Megnin |  |
|  |  |  | NHS | Universal govt funded HC | Universal insurance **Don’t know** | Non Universal insurance |  |
|  |  |  | Button Hewitt Slade Viveiros Watson |  | Bina Dennis Megnin |  |  |
| Women’s perception of HCPs knowledge OR HCPs perception about PNMH | Healthcare professionals not knowing about PNMI or the services available was a barrier to implementation, and women perceiving this to be the case was a barrier to access | Dennis & Chung-Lee, 2006; Forde et al., 2020; Hansotte et al., 2017; Jones, 2019; Megnin-Viggars et al., 2015; Rowan Stakeholder | Direct relevance UK | Partial relevance Western Country | Indirect relevance LMIC Eastern | Unclear relevance | **Minor –** UK plus real life relevance |
|  |  |  |  |  |  |  |  |
|  |  |  | Forde Rowan Stakeholder | Hansotte |  | Dennis Jones 2019 Megnin |  |
|  |  |  | NHS | Universal govt funded HC | Universal insurance **Don’t know** | Non Universal insurance |  |
|  |  |  | Forde Rowan |  | Dennis Jones 2019 Megnin | Hansotte |  |
| Women’s perception of HCPs knowledge of services/referral pathways |  | Dennis & Chung-Lee, 2006; Smith et al., 2019; Viveiros & Darling, 2018 Ganann Higgins McCauley McKenzie Ormsby Reed Rowan Beebe | Direct relevance UK | Partial relevance Western Country | Indirect relevance LMIC Eastern | Unclear relevance | **Minor -**  there is some UK relevance, but also western relevance, and a few don’t knows |
|  |  |  | Smith Viveiros KcKenzie Rowan | Gannan Higgins Ormsby Reed Beeber | McCauley | Dennis |  |
|  |  |  | NHS | Universal govt funded HC | Universal insurance **Don’t know** | Non Universal insurance |  |
|  |  |  | Smith Viveiros KcKenzie Rowan | Higgins Ormsby Reed | Dennis | Gannan Beeber McCauley |  |
| Supervision/Support (n = 6) | The opportunity for healthcare professionals to have a place to reflect and raise issues. | Boyd Gannan Munodawafa Nakku Vik Nithianden | Direct relevance UK | Partial relevance Western Country | Indirect relevance LMIC Eastern | Unclear relevance | **Moderate -**  none with direct relevance, but some partial |
|  |  |  |  | Boyd Ganann Nithianden | Munodawafa Nakku |  |  |
|  |  |  | NHS | Universal govt funded HC | Universal insurance **Don’t know** | Non Universal insurance |  |
|  |  |  |  | Nithianden Munodawafa |  | Boyd Gannan Nakku |  |
| Training (n = 27) | The opportunity for healthcare professionals to learn about perinatal mental illness and the care they are providing to women- poor or no training was associated with poor or no implementation. | Ammerman Atif 2016 Atiff 2020 Beeber Boyd Chartier Doering Drozd Eappen Feinberg Gannan Kerker Judd Kim Lind Mccaluey Mckenzie Munodowar Nithianden Noonan Reed Rowan Shorey Willey Williams Stakeholder | Direct relevance UK | Partial relevance Western Country | Indirect relevance LMIC Eastern | Unclear relevance | **None** appears universal plus real life relevance from SHs |
|  |  |  | Rowan William McKenzie Stakeholder | Ammerman Beeber Boyd Chartier Doering Drozd Feinberg Gannan Kerker Judd Kim Lind Nithianden Noonan Reed Willey | Atif Atif 20 Eappen McCauley Shorey |  |  |
|  |  |  | NHS | Universal govt funded HC | Universal insurance **Don’t know** | Non Universal insurance |  |
|  |  |  | Rowan William McKenzie SHs | Chartier Drozd Munodawafa Nithianden Noonan Shorey Willey | Eappen | Ammerman Beeber Boyd Doering Feinberg Gannan Kerker Kim Lind McCauley |  |
| HCP not recognising help-seeking | This impacted women’s access to care | Bina, 2020; Button et al., 2017; Megnin-Viggars et al., 2015; Tobin et al., 2018; Watson et al., 2019 | Direct relevance UK | Partial relevance Western Country | Indirect relevance LMIC Eastern | Unclear relevance | **Minor:**  2 direct, 1 partial |
|  |  |  | Button Watson | Tobin |  | Bina Megnin |  |
|  |  |  | NHS | Universal govt funded HC | Universal insurance **Don’t know** | Non Universal insurance |  |
|  |  |  | Button Watson | Tobin | Bina Megnin |  |  |
| Interpersonal |  |  |  |  |  |  |  |
| Language barriers (n = 10) | Difficulties in communication due to women and healthcare professionals speaking different languages affected implementation and access. | Doering Boyd Gannan Masood Munodowafa Nithiaden Pinero-leano Segre Willey Williams Button et al., 2017; Dennis & Chung-Lee, 2006; Hansotte et al., 2017; Megnin-Viggars et al., 2015; Schmied et al., 2017; Smith et al., 2019 Discussions of this in the stakeholder meetings | Direct relevance UK | Partial relevance Western Country | Indirect relevance LMIC Eastern | Unclear relevance | **None -**  mixture of all settings, countries and real life stakeholder relevance |
|  |  |  | Masood Williams Button Smith SHs | Doering Boyd Gannan Nithianden Pinero Segre Willey Hansotte Schmied | Munodawafa | Dennis Megnin |  |
|  |  |  | NHS | Universal govt funded HC | Universal insurance **Don’t know** | Non Universal insurance |  |
|  |  |  | Masood Williams Button Smith SHs | Nithianden Willey Munodawafa Schmied | Dennis Megnin | Doering Boyd Gannan Pinero Segre Hansotte |  |
| Open and honest communication (n = 11) Communication | Women and healthcare professionals being able to speak openly and honestly without fear of judgement – aids implementation . Women like to be informed of what will he happening, and for HCPs to listen to them this aids access | Doering Shakespeare Williams Vik Shorey Boyd Willey Gannan Brealey et al., 2010; Forde et al., 2020; Hadfield & Wittkowski, 2017; Hewitt et al., 2009 Stakeholder | Direct relevance UK | Partial relevance Western Country | Indirect relevance LMIC Eastern | Unclear relevance | **None -**  relevant everywhere & real life example from SH |
|  |  |  | Shakespeare Williams Brealey Forde Hadfield Hewitt | Vik Doering Boyd Willey Ganann | Shorey |  |  |
|  |  |  | NHS | Universal govt funded HC | Universal insurance **Don’t know** | Non Universal insurance |  |
|  |  |  | Shakespeare Williams Brealey Forde Hadfield Hewitt | Vik Willey |  | Doering Boyd Gannan |  |
| Information provision | Women like to have information shared with them, this aids access | Dennis Hadfield Megnin Jones 19 Randall Morrell Slade Sorsa | Direct relevance UK | Partial relevance Western Country | Indirect relevance LMIC Eastern | Unclear relevance | **Minor -**  most relevant or partially |
|  |  |  | Hadfield Slade | Randll Morrell Sorsa |  | Dennis Megnin Jones 19 |  |
|  |  |  | NHS | Universal govt funded HC | Universal insurance **Don’t know** | Non Universal insurance |  |
|  |  |  | Hadfield Slade | Morrell Sorsa | Randall Dennis Megnin Jones 19 |  |  |
| Shared decision making | Women like to make decisions about their care with HCPs, this aids access | Bina, 2020; Hadfield & Wittkowski, 2017; Megnin-Viggars et al., 2015; Morrell et al., 2016; Randall & Briscoe, 2018; Scope et al., 2017 | Direct relevance UK | Partial relevance Western Country | Indirect relevance LMIC Eastern | Unclear relevance | **Minor - -**  most relevant or partially |
|  |  |  | Hadfield | Bina Morrell Randall Scope |  | Megnin |  |
|  |  |  | NHS | Universal govt funded HC | Universal insurance **Don’t know** | Non Universal insurance |  |
|  |  |  | Hadfield | Morrell Scope | Bina Megnin Randll |  |  |
| Privacy and confidentiality (n = 8) | Women and healthcare professionals being able to interact in privacy. | Doering Feinberg Higgins Jallo Masood Omahen Pineros-Leano Pugh | Direct relevance UK | Partial relevance Western Country | Indirect relevance LMIC Eastern | Unclear relevance | **Minor - -**  most relevant or partially |
|  |  |  | Masood OMahen | Doering Feinberg Higgins Pineros Pugh |  |  |  |
|  |  |  | NHS | Universal govt funded HC | Universal insurance **Don’t know** | Non Universal insurance |  |
|  |  |  | Masood OMahen | Higgins Pugh |  | Doering Feinberg Pineros |  |
| Trusting relationship (n = 15) Relationship and rapport | Women feeling safe with healthcare professionals to be able to be open and honest about their feelings this aided implementation and access. | Atif 2016 Doering Feinberg Gannan Hadfield Higgins Kerker Leger Noonan Shakespeare Shorey Vik Willey Williams Young Bina, 2020; Brealey et al., 2010; Dennis & Chung-Lee, 2006; Hadfield & Wittkowski, 2017; Hewitt et al., 2009; Megnin-Viggars et al., 2015; Morrell et al., 2016; Scope et al., 2017; Slade et al., 2020; Tobin et al., 2018 | Direct relevance UK | Partial relevance Western Country | Indirect relevance LMIC Eastern | Unclear relevance | **None - -**  most relevant or partially & real life evidence |
|  |  |  | Hadfield Shakespeare Williams Brealey Hadfield Hewitt Slade Stakeholder | Doering Feinberg Gannan Higgins Kerker Leger Noonan Wik Willey Young Bina Morrell Scope Tobin | Atif | Dennis Megnin |  |
|  |  |  |  |  |  |  |  |
|  |  |  | NHS | Universal govt funded HC | Universal insurance **Don’t know** | Non Universal insurance |  |
|  |  |  | Hadfield Shakespeare Williams Brealey Hadfield Hewitt Slade | Higgins Noonan Vik Willey Morrell Scope Tobin | Dennis Megnin Bina | Atif Doering Feinberg Ganna Kerker Leger Young |  |
| Medication |  |  |  |  |  |  |  |
| HCPs reluctance | Healthcare professionals having little confidence in prescribing medication or knowing which medications are safe during pregnancy and breastfeeding | Ganann Byatt Higgins Noonan | Direct relevance UK | Partial relevance Western Country | Indirect relevance LMIC Eastern | Unclear relevance | **Moderrate -**  no direct relevance but some partial |
|  |  |  |  | Gannan Byatt Higgins Noonan |  |  |  |
|  |  |  | NHS | Universal govt funded HC | Universal insurance **Don’t know** | Non Universal insurance |  |
|  |  |  |  | Higgins Noonan |  | Ganann Byatt |  |
| Women’s reluctance | Women not wanting to take medication affects implementation of care and access. | Ganan Doering Williamns Young Dennis, Tobin, Jones & Sorsa, Megnin Viggers, Hadfield, Bina and Button | Direct relevance UK | Partial relevance Western Country | Indirect relevance LMIC Eastern | Unclear relevance | **Minor - -**  most relevant or partially |
|  |  |  | Williams Hadfield Button | Gannan Doering Young Tobin Sorsa Bina |  | Dennis Jones 19 Megnin |  |
|  |  |  |  |  |  |  |  |
|  |  |  | NHS | Universal govt funded HC | Universal insurance **Don’t know** | Non Universal insurance |  |
|  |  |  | Williams Hadfield Button | Tobin Sorsa | Dennis Jones 19 Megnin Bina | Ganann Doering Young |  |
| **Ideal care** |  |  |  |  |  |  |  |
| **Appropriateness** Appropriateness of care (n = 6) Appropriateness | Care sensitively designed to fit women’s needs improved implementation and access | Chartier Gannan Nithianden Noonan Shakespeare Evans Megnin Scope | Direct relevance UK | Partial relevance Western Country | Indirect relevance LMIC Eastern | Unclear relevance | **Moderate-**  only one supporting study and it is about screening |
|  |  |  | Shakespeare | Chartier Gannan Nithianden Noonan Evans Scope |  | Megnin |  |
|  |  |  |  |  |  |  |  |
|  |  |  |  |  |  |  |  |
|  |  |  | NHS | Universal govt funded HC | Universal insurance **Don’t know** | Non Universal insurance |  |
|  |  |  | Shakespeare | Chartoer Nithianden Nooan | Megnin | Ganann |  |
| Culturally appropriate | In line with this care needed to be culturally appropriate to fit to women’s individual cultural needs | Button Dennis Hadfield Jones 19 Schmied Smith Tobin Watson | Direct relevance UK | Partial relevance Western Country | Indirect relevance LMIC Eastern | Unclear relevance | **Minor - -**  most relevant or partially |
|  |  |  | Button Hadfield Smith Watson | Tobin Schmied |  |  |  |
|  |  |  | NHS | Universal govt funded HC | Universal insurance **Don’t know** | Non Universal insurance |  |
|  |  |  | Button Hadfield Smith Watson | Tobin Schmied | Dennis Jones 19 |  |  |
| **Woman-centred** Choice (n = 6) | Women being able to choose their care improved implementation | Chartier Doering Ganann Ormsby Vik Segre Stakeholders | Direct relevance UK | Partial relevance Western Country | Indirect relevance LMIC Eastern | Unclear relevance | **Mild-**  no direct relevance, only partial but evidence from SHs |
|  |  |  | SH | Chartier Doering Ganann Ormsby Vik Segre |  |  |  |
|  |  |  | NHS | Universal govt funded HC | Universal insurance **Don’t know** | Non Universal insurance |  |
|  |  |  |  | Chartier Vik Ormsby |  | Doering Gannan Segre |  |
| Fitting in with women’s lifestyle (n = 3) | Interventions that fit easily into women’s lifestyles aided implementation. | Chartier Omahen Pugh | Direct relevance UK | Partial relevance Western Country | Indirect relevance LMIC Eastern | Unclear relevance | **Minor - -**  most relevant or partially |
|  |  |  | OMahen | Chartier Pugh |  |  |  |
|  |  |  | NHS | Universal govt funded HC | Universal insurance **Don’t know** | Non Universal insurance |  |
|  |  |  | OmAHEN | Chartier Pugh |  |  |  |
| Flexibility (n = 13) | Flexibility of care improved implementation | Bina Doering Eappen Ganann Hadfield Judd Munoda Nithian Omahen Pugh Shorey Williams Atif 2020 Scope | Direct relevance UK | Partial relevance Western Country | Indirect relevance LMIC Eastern | Unclear relevance | **Minor -**  seems to be quite a universal desire |
|  |  |  | Hadfield O’Mageb Williams | Doering Ganann Judd Nithianden Pugh Scope | Bina Eappen Munodawafa Shorey Atif 20 |  |  |
|  |  |  | NHS | Universal govt funded HC | Universal insurance **Don’t know** | Non Universal insurance |  |
|  |  |  | Hadfield O’Mageb Williams | Judd Nithianden Pugh Munodawafa Shorey Scope | Bina Eappen | Doering Gannan Atif |  |
| Relevance to women (n = 2) | Being aware of patient’s needs and providing care with women at the centre aided implementation and access Relevance to women improved implementation | Omahen Pugh | Direct relevance UK | Partial relevance Western Country | Indirect relevance LMIC Eastern | Unclear relevance | **Minor - -**  most relevant or partially |
|  |  |  | O Mahen | Pugh |  |  |  |
|  |  |  | NHS | Universal govt funded HC | Universal insurance **Don’t know** | Non Universal insurance |  |
|  |  |  | OmAHEN | pUGH |  |  |  |
| Individualised |  | Evans et al., 2020; Hadfield & Wittkowski, 2017; Megnin-Viggars et al., 2015; Morrell et al., 2016; Schmied et al., 2017; Scope et al., 2017; Slade et al., 2020; Sorsa et al., 2021; Viveiros & Darling, 2018; Watson et al., 2019 | Direct relevance UK | Partial relevance Western Country | Indirect relevance LMIC Eastern | Unclear relevance | **Minor - -**  most relevant or partially |
|  |  |  | Hadfield Slade Viveiros Watson | Evans Morrell Schmied Scope Sorsa |  | Megnin |  |
|  |  |  | NHS | Universal govt funded HC | Universal insurance **Don’t know** | Non Universal insurance |  |
|  |  |  | Hadfield Slade Viveiros Watson | Evans Morrell Schmied Scope Sorsa | Megnin |  |  |
| Length | Care needed to be at an appropriate length for women, not to finish too early to aid access | Hadfield & Wittkowski, 2017; Morrell et al., 2016; Schmied et al., 2017; Watson et al., 2019 | Direct relevance UK | Partial relevance Western Country | Indirect relevance LMIC Eastern | Unclear relevance | **Minor - -**  most relevant or partially |
|  |  |  | Hadfield Watson | Morrell Schmied |  |  |  |
|  |  |  | NHS | Universal govt funded HC | Universal insurance **Don’t know** | Non Universal insurance |  |
|  |  |  | Hadfield Watson | Morrell Schmied |  |  |  |
| Timing (n = 5) | Implementation was aided when the timing fitted in with women’s needs | Pugh Segre Leger Masood | Direct relevance UK | Partial relevance Western Country | Indirect relevance LMIC Eastern | Unclear relevance | **Minor - -**  most relevant or partially |
|  |  |  | Masood | Pugh Segre Leger |  |  |  |
|  |  |  | NHS | Universal govt funded HC | Universal insurance **Don’t know** | Non Universal insurance |  |
|  |  |  | Masood | Pugh Leger |  | Segre |  |
| **How and where delivered** Delivery in a healthcare setting (n = 12) | Generally women prefer treatment at their homes. Healthcare settings were seen by women as being too busy. Implementation was aided when services acted as a “one stop shop” | Atif 20 Bina Boyd Cox Kerker Munodawafa Ormsby | Direct relevance UK | Partial relevance Western Country | Indirect relevance LMIC Eastern | Unclear relevance | **Moderate** some partially releavant |
|  |  |  |  | Boyd Cox Kerker Ormsby | Atif 20 Bina Munodawafa |  |  |
|  |  |  | NHS | Universal govt funded HC | Universal insurance **Don’t know** | Non Universal insurance |  |
|  |  |  |  | Ormsby Munodawafa | Bina | Atif 20 Boyd Cox Kerker |  |
| Delivery in home setting (n = 5) |  | Ammerman Beeber Judd Munodawafa Myors Leger Hadfield Hansotte Jones 19 Praetorius Sorsa | Direct relevance UK | Partial relevance Western Country | Indirect relevance LMIC Eastern | Unclear relevance | **Moderate-**  only one supporting study |
|  |  |  | Hadfield | Ammerman Beeber Judd Myors Leger Hansotte Praetorius Sorsa | Munodawafa | Jones 19 |  |
|  |  |  | NHS | Universal govt funded HC | Universal insurance **Don’t know** | Non Universal insurance |  |
|  |  |  | Hadfield | Judd Myors Sorsa |  | Ammerman Beeber Leger Hansotte Praetorius |  |
| Location |  | Ganann Judd Leger Ormsby Jones et al., 2014; Mollard et al., 2016; Newman et al., 2019; | Direct relevance UK | Partial relevance Western Country | Indirect relevance LMIC Eastern | Unclear relevance | **Moderate:** no direct relevance but all partial |
|  |  |  |  | Gannan Judd Leger Ormsby Jones 14 Mollard Newman |  |  |  |
|  |  |  | NHS | Universal govt funded HC | Universal insurance **Don’t know** | Non Universal insurance |  |
|  |  |  |  | Judd Ormsby Jones 14 Newman |  | Gannan Leger Mollard |  |
| Face to face delivery (n = 4) | Provision of face to face care, as opposed to over the phone or online improved implementation. | Omahen Doering Shorey Pugh Stakeholder | Direct relevance UK | Partial relevance Western Country | Indirect relevance LMIC Eastern | Unclear relevance | **None:** seems universal & evidence from SH meeting |
|  |  |  | OMahen Stakeholder | Doering Pugh | Shorey |  |  |
|  |  |  | NHS | Universal govt funded HC | Universal insurance **Don’t know** | Non Universal insurance |  |
|  |  |  | OMahen | Pugh Shorey | Doering |  |  |
| Groups/peer support | Some women want group/peer support, others don’t | Hadfield 2019 Masood 2015 Nakku Dennis Evans Hadfield Jones 14 Megnin Morrell Schmied Scope Slade Tobin Watson Stakeholder | Direct relevance UK | Partial relevance Western Country | Indirect relevance LMIC Eastern | Unclear relevance | **None- -**  most relevant or partially plus real life evidence |
|  |  |  | Hadfield Masood Hadfield sr Slade Watson SH | Evans Jones 14 Morrell Schmied Scope Tobin | Nakku | Dennis Megnin |  |
|  |  |  | NHS | Universal govt funded HC | Universal insurance **Don’t know** | Non Universal insurance |  |
|  |  |  | Hadfield Masood Hadfield sr Slade Watson | Evans Jones 14 Morrell Schmied Scope Tobin | Dennis Megnin |  |  |
| Validation | And this provided them with validation for their feelings | Jones et al., 2014; Morrell et al., 2016; Schmied et al., 2017; Slade et al., 2020; Praetorius | Direct relevance UK | Partial relevance Western Country | Indirect relevance LMIC Eastern | Unclear relevance | **Moderate:**  heavily partially relevant, only one direct |
|  |  |  | Slade | Jones 14 Morrell Schmied Praetorius |  |  |  |
|  |  |  | NHS | Universal govt funded HC | Universal insurance **Don’t know** | Non Universal insurance |  |
|  |  |  | Slade | Morrell Schmied |  | Praetorius |  |
| **Content** Manualised therapy (n = 2) | Interventions performed according to specific guidelines sometimes helped with implementation but could feel too rigid. | Atif 2020 | Direct relevance UK | Partial relevance Western Country | Indirect relevance LMIC Eastern | Unclear relevance | **Severe:**  carried out in LMIC country |
|  |  |  |  |  | Atif 20 |  |  |
|  |  |  | NHS | Universal govt funded HC | Universal insurance **Don’t know** | Non Universal insurance |  |
|  |  |  |  |  |  | Atif 20 |  |
| Techniques women found useful (n = 4) | Most women found personalised therapy that challenged their thoughts and beliefs (e.g. CBT approaches) useful therefore aiding implementation | Omahen Masood Pugh Hadfield | Direct relevance UK | Partial relevance Western Country | Indirect relevance LMIC Eastern | Unclear relevance | **None:**  majority have direct relevance |
|  |  |  | OmAHEN Masood Hadfield | Pugh |  |  |  |
|  |  |  | NHS | Universal govt funded HC | Universal insurance **Don’t know** | Non Universal insurance |  |
|  |  |  | OmAHEN Masood Hadfield | Pugh |  |  |  |
| Opportunity to talk | Having someone to talk to about their difficulties was a facilitator to access | Dennis & Chung-Lee, 2006; Evans et al., 2020; Hadfield & Wittkowski, 2017; Jones, 2019; Kassam, 2019; Morrell et al., 2016; Praetorius et al., 2020; Staneva et al., 2015 Megnin | Direct relevance UK | Partial relevance Western Country | Indirect relevance LMIC Eastern | Unclear relevance | **Moderater -**  only 1 relevant |
|  |  |  | Hadfield | Evans Kassam Morrell Praetorius Staneva |  | Dennis Jones 19 Megnin |  |
|  |  |  | NHS | Universal govt funded HC | Universal insurance **Don’t know** | Non Universal insurance |  |
|  |  |  | Hadfield | Evans Kassam Morrell Staneva | Dennis Jones Praetorius Megnin |  |  |
| Information provision | Women valued care that provided information about PNMH and options available for help and support, thus aiding access | Forde et al., 2020; Hadfield & Wittkowski, 2017; A. Jones, 2014; Megnin-Viggars et al., 2015; Morrell et al., 2016; Schmied et al., 2017; Scope et al., 2017; Slade et al., 2020; Sorsa et al., 2021 Tobin | Direct relevance UK | Partial relevance Western Country | Indirect relevance LMIC Eastern | Unclear relevance | **Minor - -**  most relevant or partially s |
|  |  |  | Forde Hadfield Slade | Jones 14 Morrell Schmied Scope Sorsa Tobin |  | Megnin |  |
|  |  |  | NHS | Universal govt funded HC | Universal insurance **Don’t know** | Non Universal insurance |  |
|  |  |  | Forde Hadfield Slade | Jones 14 Morrell Schmied Scope Sorsa Tobin |  |  |  |
| Technology (n = 11) | The use of technology to provide care and treatment such as reminders on screens, completing assessment questionnaires on tablets, communicating via Whatsapp, or completing therapy online aided implementation **when it was working.** | Feinberg Fernandez Jallo Kim Lind Noonan Pinero-leano Shorey Willey Williams Doering Stakeholder | Direct relevance UK | Partial relevance Western Country | Indirect relevance LMIC Eastern | Unclear relevance | **None:** although only one study supports, we have real life evidence from the stakeholders – this came across strongly in multiple stakeholder groups |
|  |  |  | Williams SH | Feinberg Fernandez Jallo Kim Lind Noonan Pineros Willey Doering | Shorey |  |  |
|  |  |  | NHS | Universal govt funded HC | Universal insurance **Don’t know** | Non Universal insurance |  |
|  |  |  | Williams SH | Noonan Willey Shorey |  | Feinberg Fernandez Jallo Kim Lind Pinero Doering |  |
| **Organisational aspects** Open inclusion criteria (n = 2) | Open inclusion criteria was useful for implementation . | Judd Young | Direct relevance UK | Partial relevance Western Country | Indirect relevance LMIC Eastern | Unclear relevance | **Moderate –** none are directly relevant, but have partial relevance |
|  |  |  |  | Judd Young |  |  |  |
|  |  |  | NHS | Universal govt funded HC | Universal insurance **Don’t know** | Non Universal insurance |  |
|  |  |  |  | Judd |  | Young |  |
| Practical support (n = 10) | The provision of support that allowed women to access care, such as providing a creche or paying for travel aided implementation | Shorey Cox Munodawafa Ganan Hadfield Masood Nakku Nithianden Ormsby Button Forde Jones 19 Newman | Direct relevance UK | Partial relevance Western Country | Indirect relevance LMIC Eastern | Unclear relevance | **minor -**  seems pretty universal |
|  |  |  | Hadfield Masood Button Forde | Cox Gannan Nithianden Ormsby Newman | Shorey Munodawafa Nakku | Jones 19 |  |
|  |  |  | NHS | Universal govt funded HC | Universal insurance **Don’t know** | Non Universal insurance |  |
|  |  |  | Hadfield Masood Button Forde | Nithinden Ormsby Newman Shorey Munodawafa | Jones 19 | Cox Gannan Nakku |  |
| **Perception of care** Healthcare professional’s perception (n = 19) | Positive views of care by HCPs aided implementation | Ammerman Atifd 2016 Atif 2020 Bina Boyd Chartier Dierubg Drozd Feinberg Kim Leger McKenzieMc Munodowafa Nakku Nithian Ormsby Reed Segre Shorey Vik Willey | Direct relevance UK | Partial relevance Western Country | Indirect relevance LMIC Eastern | Unclear relevance | **Moderate -**  only 1 relevant |
|  |  |  | McKenzie | Ammerman Boyd Chartier Doering Drozd Feinberg Kim Leger Nithianden Ormsby Reed Segrey Vik Willey | Atif 16 Atif 20 Bina Munodawafa Nakku Shorey |  |  |
|  |  |  | NHS | Universal govt funded HC | Universal insurance **Don’t know** | Non Universal insurance |  |
|  |  |  | McKenzie | Chartier Drozd Leger Nithianden Ormsby Wik Willey Shorey | Bina | Ammerman Boyd Doering Feinberg Kim Segre Ati 20 Atif 16 Nakku |  |
| Women’s perception of the care (n = 7) | Positive views of women about care aided implementation | Ammerman Atif 2016 Atif 2020 McKenzie Ormsby | Direct relevance UK | Partial relevance Western Country | Indirect relevance LMIC Eastern | Unclear relevance | **Moderate -**  only 1 relevant |
|  |  |  | McKenzie | Ammerman Ormsby | Atif 16 Atif 20 |  |  |
|  |  |  | NHS | Universal govt funded HC | Universal insurance **Don’t know** | Non Universal insurance |  |
|  |  |  | McKenzie | rmsby |  | Ammerman Atif 16 Atif 20 |  |
| **Screening and assessment** Wording of assessment tools (n = 3) Wording of screening tools | The clarity of the wording of the assessment tools affects implementation and access | Williams Doering Segre Brealey et al., 2010; Hewitt et al., 2009 | Direct relevance UK | Partial relevance Western Country | Indirect relevance LMIC Eastern | Unclear relevance | **None-**  most are directly relevant |
|  |  |  | Brealey Hewitt Williams | Doering Segre |  |  |  |
|  |  |  | NHS | Universal govt funded HC | Universal insurance **Don’t know** | Non Universal insurance |  |
|  |  |  | Brealey Hewitt Williams |  |  | Doering Segre |  |
| Delivery of screening | Women did not like it when delivery was done in a tick box way with no time for discussion and no explanation beforehand Explaining the purpose of assessment to women. | Brealey et al., 2010; Button et al., 2017; Evans et al., 2020; Hewitt et al., 2009; Smith et al., 2019 Nithianden Shakespeare Noonan Segre Vik Williams Doering | Direct relevance UK | Partial relevance Western Country | Indirect relevance LMIC Eastern | Unclear relevance | **Minor - -**  most relevant or partially s |
|  |  |  |  |  |  |  |  |
|  |  |  | Brealey Button Hewitt Smith Shakespeare Williams | Evans Nithianden Noonan Sgre Vik Doering |  |  |  |
|  |  |  | NHS | Universal govt funded HC | Universal insurance **Don’t know** | Non Universal insurance |  |
|  |  |  | Brealey Button Hewitt Smith Shakespeare Williams | Evans Nithianden Vik Noonan |  | Segre Doering |  |
| Screening acceptability | EPDS was generally found to be acceptable to Native/Indigenous women of Canada, South Asian women living in the UK, and Black Caribbean women living in the UK However, certain questions may not elicit true feelings from Vietnamese women living in the UK because of the shame of admitting to these Q10 on the EPDS (‘the thought of harming myself has occurred to me’) was seen as problematic to Arabic, Vietnamese and Black Caribbean mothers living in the UK or USA, | Brealey et al., 2010; Button et al., 2017; Evans et al., 2020; Hewitt et al., 2009; Megnin-Viggars et al., 2015; Mollard et al., 2016; Smith et al., 2019 Doeriing Shakespeare Stakeholder | Direct relevance UK | Partial relevance Western Country | Indirect relevance LMIC Eastern | Unclear relevance | **None –**majority directly related |
|  |  |  | Brealey Button Hewitt Smith Shakespeare | Evans Mollard Doering |  | Megnin |  |
|  |  |  | NHS | Universal govt funded HC | Universal insurance **Don’t know** | Non Universal insurance |  |
|  |  |  | Brealey Button Hewitt Smith Shakespeare | Evan |  | Mollard Doering |  |
| Organisational |  |  |  |  |  |  |  |
| Continuity of carer (n = 8) | Women being able to see the same person across the care pathway was helpful in terms of implementation and access to PNMH care. | Byatt Chartier Higgins Nithianden OMahen Rowan Willey Williams Brealey Button Hadfileld Menin Slade Smith Tobin Viveiros Watson Stakeholder | Direct relevance UK | Partial relevance Western Country | Indirect relevance LMIC Eastern | Unclear relevance | **None/v minor -**  most studies are directly relevant + evidence from SH meeting |
|  |  |  | Rowan O Mahen Williams Brealey Button Hadfield Slade Smith Viveiros Watson | Byatt Chartier Higgins Niithianden Willey Tobin |  | Megnin |  |
|  |  |  | NHS | Universal govt funded HC | Universal insurance **Don’t know** | Non Universal insurance |  |
|  |  |  | Rowan O Mahen Williams Brealey Button Hadfield Slade Smith Viveiros Watson SH | Chartier Higgins Nithianden | Megnin | Byatt |  |
| Organisational structure (n = 5) | A structure in which the purpose of each part of the organisation is clear and meets all of women’s needs, ensuring women don’t fall through gaps for implementation | Rowan Higgins Judd Vik Pugh | Direct relevance UK | Partial relevance Western Country | Indirect relevance LMIC Eastern | Unclear relevance | **Moderate-**  most partial relevance |
|  |  |  | Rowan | Higgins Jud Vik Pugh |  |  |  |
|  |  |  | NHS | Universal govt funded HC | Universal insurance **Don’t know** | Non Universal insurance |  |
|  |  |  | Rowan SH | Higgins Jud Vik Pugh |  |  |  |
| Referral pathways (n = 12) | Having a clear and easy process to allow for women to be referred to other services helps implement a new service. | Ammerman Bina Judd Boyd Cox Feinberg Nithianden Kerker Noonan Willey Rowan Stakeholder | Direct relevance UK | Partial relevance Western Country | Indirect relevance LMIC Eastern | Unclear relevance | **Minor -**  most partial relevance but came up in SH meetings so real life relevance |
|  |  |  | Rowan SH | Ammerman Judd Boyd Cox Feinberg Nithianden Noonan Willey | Bina |  |  |
|  |  |  | NHS | Universal govt funded HC | Universal insurance **Don’t know** | Non Universal insurance |  |
|  |  |  | Rowan SH | Judd Nithianden Noonan Willey | Bina | Ammerman Cox Feinberg |  |
| Workflow procedures (n = 13) | Knowledge of job roles and processes within the organisation. The understanding of which tasks need to be done by whom, and how to achieve these tasks helps with implementation. Things the organisation has put in place to help with implementation | Feinberg Fernandez Kim Lind Mckenzie Segre Munodawafa Nithaianden Doering Noonan Gannan Pugh Stakeholder | Direct relevance UK | Partial relevance Western Country | Indirect relevance LMIC Eastern | Unclear relevance | **Minor -**  most partial relevance but came up in SH meetings so real life relevance |
|  |  |  | McKNZIE SH | Feinberg Fernandez Kim Lind Segre Nithianden Doering Noonan Gannan Pugh | Munodawafa |  |  |
|  |  |  | NHS | Universal govt funded HC | Universal insurance **Don’t know** | Non Universal insurance |  |
|  |  |  | McKenzie SH | Munodawafa Noonan Pugh Segre |  | Feeinberg Fernandez Kim Lind Segre Doering Ganann |  |
| Lack of appropriate or timely services (n = 9) Lack of services/overstretched | Not having anywhere to refer women on to due to lack of services or being able to refer women on to other services but the waiting time being long. This prevented women accessing care because no service would take them, long waiting times, structural barriers, needed to be in a crisis | Boyd Doering Ganann Higgins Kerker Lomanohaycroft Noonan Leger Rowan Williams Bina, 2020; Button et al., 2017; Forde et al., 2020; Jones, 2019; Megnin-Viggars et al., 2015; Smith et al., 2019; Tobin et al., 2018; Viveiros & Darling, 2018 Stakeholder | Direct relevance UK | Partial relevance Western Country | Indirect relevance LMIC Eastern | Unclear relevance | **None -**  more are of partial relevance but came up in SH meetings so real life relevance |
|  |  |  | Rowan Williams Button Forde Smith Viveiros SH | Boyd Doering Ganann Higgins Kerker Lomano Haycroft BinaSR Tobin |  | Megnin Jones 19 |  |
|  |  |  | NHS | Universal govt funded HC | Universal insurance **Don’t know** | Non Universal insurance |  |
|  |  |  | Rowan Williams Button Forde Smith Viveiros SH | Higgins Kerker Tobin | Megnin Jones 19 Bina | Boyd Doering Gannan Kerker Lomano |  |
| Resources (n = 5) | Not having enough resources within organisations e.g. medication or support staff means cant implement a service properly Stakeholders | Boyd Keker Nakku | Direct relevance UK | Partial relevance Western Country | Indirect relevance LMIC Eastern | Unclear relevance | **Minor:** most of partial relevance, but there is real life evidence from SH groups about workforce |
|  |  |  | SH | Boyd Kerker | Nakku |  |  |
|  |  |  | NHS | Universal govt funded HC | Universal insurance **Don’t know** | Non Universal insurance |  |
|  |  |  | SH |  |  | Boyd Kerker Nakku |  |
| Service integration (n = 6) Collaboration within and across services | The linking up of different services who deal with different needs, or at different time points across the perinatal period benefitted women, stopped them falling through the net – implementation + access | Ammerman Atif Atif 2020 Judd Noonan Young Rowan Bina, 2020; Megnin-Viggars et al., 2015; Newman et al., 2019; Smith et al., 2019; Watson et al., 2019 Forde Stakeholder | Direct relevance UK | Partial relevance Western Country | Indirect relevance LMIC Eastern | Unclear relevance | **None:** more are of partial relevance, but there is real life evidence from SH |
|  |  |  | Rowan Smith Watson Forde SH | Ammerman Judd Noonn Young BinaSR Newman | Atif Atif 20 | Megnin |  |
|  |  |  | NHS | Universal govt funded HC | Universal insurance **Don’t know** | Non Universal insurance |  |
|  |  |  | Rowan Smith Watson Forde SH | Noonan Newman | Bina SR Megnin | Ammerman Judd Young Atif Atif 20 |  |
| Timely follow up (n = 3) | Women being followed up by healthcare professionals or other services in short time frame means you can keep women on care pathway aiding implementation | Vik Myors Nithianden | Direct relevance UK | Partial relevance Western Country | Indirect relevance LMIC Eastern | Unclear relevance | **Moderate:** none are of direct relevance |
|  |  |  |  | Vik Myors Nithianden |  |  |  |
|  |  |  | NHS | Universal govt funded HC | Universal insurance **Don’t know** | Non Universal insurance |  |
|  |  |  |  | Vik Myors Nithianden |  |  |  |
| Polictics |  |  |  |  |  |  |  |
| Funding (n = 10) | Financial resources needed to provide and implement perinatal mental healthcare. | Ammerman Cox Doering Feinberg Friendman Gannan Lomanon Kim Ormsby Rowan Stakeholder | Direct relevance UK | Partial relevance Western Country | Indirect relevance LMIC Eastern | Unclear relevance | **None:** Most are of partial relevance, but there is real life evidence from SH |
|  |  |  | Rowan SH | Ammerman Cox Doering Feinberg Friedman Ganann Lomano Kim Ormsby |  |  |  |
|  |  |  | NHS | Universal govt funded HC | Universal insurance **Don’t know** | Non Universal insurance |  |
|  |  |  | Rowan SH | Ormsby |  | Ammerman Cox Doering Gannan Feinberg Friedman Lomano Kim |  |
| Immigration status | Immigration status influenced women’s *decision to consult* and access to care due to fears of being deported | Bina, 2020; Giscombe et al., 2020; Hansotte et al., 2017; Kassam, 2019; Schmied et al., 2017 Ganann Stakeholder – vulnerable women | Direct relevance UK | Partial relevance Western Country | Indirect relevance LMIC Eastern | Unclear relevance | **Moderate:** vulnerable women did come up in stakeholder but no other studies |
|  |  |  | SH | Bina Giscombe Kassam Hansotte Schmied Ganann |  |  |  |
|  |  |  | NHS | Universal govt funded HC | Universal insurance **Don’t know** | Non Universal insurance |  |
|  |  |  | SH | Giscombe Kassam Schmied | Bina | Hansotte Gannan |  |
| Economic status Healthcare costs | This influenced womens decision to consult, access to care (because can’t afford it) but also their experience of care because cant find wellbeing when you cant fulfil basic needs Costs of healthcare related to the above point. If you cant afford it, you don’t consult | Schmied Tobin Lucas Kassam Bina Hansotte Viveiros Jones 19 | Direct relevance UK | Partial relevance Western Country | Indirect relevance LMIC Eastern | Unclear relevance | **Moderate:** more relevant to places where you have to pay for healthcare |
|  |  |  | Viveiros | Schmied Tobin Lucas Kassam Bina Hansotte |  | Jones 19 |  |
|  |  |  | NHS | Universal govt funded HC | Universal insurance **Don’t know** | Non Universal insurance |  |
|  |  |  |  | Schmied Tobin Kassam | Bina | Hansotte Lucas |  |
| Societal |  |  |  |  |  |  |  |
| Stigma | Stigma prevented implementation and access to care. it was pervasive. | Atif 2016 Atif 2020 Beeber Bina Boyd Chartier Cox Feinberg Higgins Keker MCcAULEY McKenzie Munodawafa Myors Nakku Nithianden Noonan Shakesperare Vik Williams Young Bina Button Dennis Forde Giscombe Hadfield Hansottee Holopainen Hewitt 19 Jones 19 Kassam Lucas Megnin Mollad Nilaweera Smith Sorsa Staneva Tobin Viveiros Watson | Direct relevance UK | Partial relevance Western Country | Indirect relevance LMIC Eastern | Unclear relevance | **None –** a universal theme with real life support |
|  |  |  | McKenzie Shakespeare Williams Button Forde Hadfield Hewitt Smith Viveiros Watson SH | Beeber Boyd Chartier Cox Feinberg Higgins Kerker Myors Nithianden Noonan Vik Young BinaSR Giscombe Hansotte Holopainen Kassam Lucas Mollard Nilaweera Sorsa Staneva Tobin | Atif Atif 20 Bina McCauley Munodawafa Nakku | Dennis Jones 19 Megnin |  |
|  |  |  | NHS | Universal govt funded HC | Universal insurance **Don’t know** | Non Universal insurance |  |
|  |  |  |  |  |  |  |  |
| Culture (n = 13) | The beliefs and behaviours of a particular group of people or a society. Certain cultural beliefs about mental illness (i.e. it isn’t real, or its caused by spirits) means people are less likely to consult. Culture and stigma interlink. You have to be strong, have to fulfil gender roles, punishment for being black (more likely to be labelled) | Atif 2016 Bina Boyd Friedman Ganann Higgins Masood McCauley Naku Nithanden Noonan Segre Shorey Bina, 2020; Brealey et al., 2010; Button et al., 2017; Dennis & Chung-Lee, 2006; Giscombe et al., 2020; Hansotte et al., 2017; Hewitt et al., 2009; Jones, 2019; Kassam, 2019; Megnin-Viggars et al., 2015; Nilaweera et al., 2014; Praetorius et al., 2020; Schmied et al., 2017; Smith et al., 2019; Sorsa et al., 2021; Staneva et al., 2015; Tobin et al., 2018; Viveiros & Darling, 2018; Watson et al., 2019; Wittkowski et al., 2014 Stakeholders | Direct relevance UK | Partial relevance Western Country | Indirect relevance LMIC Eastern | Unclear relevance | **None –** a universal theme |
|  |  |  | Masood Brealey Button Hewitt Smith Watson | Boyd Friedman Ganann Higgins Nithianden Segre Bina SR Hansotte Giscombe Kassam Nilaweera Praetorius Schmied Sorsa Staneva Tobin | Atif 1 Bina McCauley Nakku Shorey Witkowski | Dennis Jones 19 Megnin |  |
|  |  |  | NHS | Universal govt funded HC | Universal insurance **Don’t know** | Non Universal insurance |  |
|  |  |  |  |  |  |  |  |
| Maternal norms | The **maternal norm** for women to show they are strong, that they can cope and be a good mother (n = 19), prevented women from *deciding to consult, disclosing, accessing care* and *women’s experience of care* | Bina, 2020; Brealey et al., 2010; Button et al., 2017; Dennis & Chung-Lee, 2006; Forde et al., 2020; Hadfield & Wittkowski, 2017; Hansotte et al., 2017; Hewitt et al., 2009; Holopainen & Hakulinen, 2019; Jones et al., 2014; Lucas et al., 2019; Mollard et al., 2016; Morrell et al., 2016; Newman et al., 2019; Schmied et al., 2017; Slade et al., 2020; Smith et al., 2019; Sorsa et al., 2021; Staneva et al., 2015; Viveiros & Darling, 2018 | Direct relevance UK | Partial relevance Western Country | Indirect relevance LMIC Eastern | Unclear relevance | **None/v minor:**  a universal theme |
|  |  |  | Brealey Button Forde Hadfield Hewitt Slade Smith Viveiros | BinaSR Hansotte Holopainen Jones14 Lucas Mollard Morrell Newman Schmied Sorsa Staneva |  | Dennis Jones 19 |  |
|  |  |  | NHS | Universal govt funded HC | Universal insurance **Don’t know** | Non Universal insurance |  |
|  |  |  |  |  |  |  |  |

# *Supplementary Materials 9:* GRADE-CERQual overall confidence of concepts table

| **Theme** | **Definition of theme** | **Studies citing this theme** | **N** | **Methodological rating** | **Adequacy rating** | **Coherence rating** | **Relevance rating** | **Overall CERQUAL Rating** |
| --- | --- | --- | --- | --- | --- | --- | --- | --- |
| **1 Women** | | | | | | | | |
| **1.1 Beliefs about health services** | | | | | | | | |
| 1.1.1 Services only offer medication | The belief that health services will only offer medication to treat PMH concerns | *Doering et al., 2017; Ganann et al., 2019; Williams et al., 2016; Young et al., 2019;*  Bina, 2020; Button et al., 2017; Dennis & Chung-Lee, 2006; Hadfield & Wittkowski, 2017; Jones, 2019; Megnin-Viggars et al., 2015; Nilaweera et al., 2014; Sorsa et al., 2021; Tobin et al., 2018 | 14 | Moderate confidence | Low confidence | High confidence | Low confidence | **Low confidence** |
| 1.1.2 Services are stretched | The belief that PMH services are too stretched and will therefore be unable to help women | Dennis & Chung-Lee, 2006; Hadfield & Wittkowski, 2017 | 2 | Low confidence | Very low confidence | Low confidence | Moderate confidence | **Low confidence** |
| 1.1.3 Services are too complicated | Services being too complex or complicated | *Ganann et al., 2019*  Tobin et al., 2018  SH | 2 | Moderate confidence | Very low confidence | High confidence | Moderate confidence | **Low confidence** |
| 1.1.4 Women’s mistrust and fear of services | Having little trust in health services | *Boyd et al., 2011*  Jones, 2019 | 2 | Moderate confidence | Very low confidence | High confidence | Low confidence | **Low confidence** |
| **1.2 Beliefs about HCPs** | | | | | | | | |
| 1.2.1 Not understanding HCPs’ role | Not understanding the roles of HCPs and how their roles related to PMH | Brealey et al., 2010; Button et al., 2017; Dennis & Chung-Lee, 2006; Hadfield & Wittkowski, 2017; Hewitt et al., 2009; Megnin-Viggars et al., 2015; Mollard et al., 2016; Morrell et al., 2016; Nilaweera et al., 2014; Sambrook Smith et al., 2019; Schmied et al., 2017; Scope et al., 2017 | 12 | Low confidence | Low confidence | Moderate confidence | Moderate confidence | **Moderate confidence** |
| 1.2.2 Believing HCPs won’t be interested | Believing HCPs won't be interested in PMH | Bina, 2020; Hadfield & Wittkowski, 2017 | 2 | Moderate confidence | Very low confidence | Low confidence | Moderate confidence | **Low confidence** |
| **1.3 Beliefs about perinatal mental illness** | | | | | | | | |
| **1.3.1 What is it?** | | | | | | | | |
| 1.3.1.1 What is perinatal mental illness? | Having poor or no knowledge about PMI | *Atif et al., 2019; Kerker et al., 2018*  Bina, 2020; Button et al., 2017; Dennis & Chung-Lee, 2006; Hadfield & Wittkowski, 2017; Hansotte et al., 2017; A. Jones, 2019; Lucas et al., 2019; Megnin-Viggars et al., 2015; Morrell et al., 2016; Newman et al., 2019; Sambrook Smith et al., 2019; Schmied et al., 2017; Scope et al., 2017; Staneva et al., 2015; Tobin et al., 2018; Watson et al., 2019 | 18 | Low confidence | Moderate confidence | High confidence | Moderate confidence | **Moderate confidence** |
| 1.3.1.2 No language to describe perinatal mental illness | Not having the language to describe PMI | *Bina et al., 2018*  Brealey et al., 2010; Staneva et al., 2015; Tobin et al., 2018; Watson et al., 2019 | 5 | Moderate confidence | Very low confidence | Moderate confidence | Moderate confidence | **Low confidence** |
| **1.3.2 Causes of perinatal mental illness** | | | | | | | | |
| 1.3.2.1 Spiritual/cultural causes | Believing that symptoms are caused by cultural or spiritual factors | *Atif et al., 2016; McCauley et al., 2019; Nakku et al., 2016*  Button et al., 2017; Schmied et al., 2017; Wittkowski et al., 2014 | 6 | Moderate confidence | Low confidence | Moderate confidence | Moderate confidence | **Moderate confidence** |
| 1.3.2.2 External causes | Believing that symptoms are caused by external factors such as jobs, being a migrant | Bina, 2020; Button et al., 2017; Dennis & Chung-Lee, 2006; Lucas et al., 2019; Schmied et al., 2017; Staneva et al., 2015; Tobin et al., 2018; Watson et al., 2019 | 8 | Moderate confidence | Low confidence | Moderate confidence | Low confidence | **Low confidence** |
| 1.3.2.3 Physical causes | Believing that symptoms are caused by physical factors such as tiredness and hormones | *O’Mahen & Flynn, 2008*  Bina, 2020; Button et al., 2017; Dennis & Chung-Lee, 2006; Forde et al., 2020; C. C. G. Jones et al., 2014; Newman et al., 2019; Sambrook Smith et al., 2019; Schmied et al., 2017; Staneva et al., 2015; Watson et al., 2019 | 13 | Low confidence | Low confidence | Moderate confidence | Moderate confidence | **Low confidence** |
| 1.3.2.4 A normal response to motherhood? | Believing symptoms are just a normal response to motherhood | *Williams et al., 2016*  Dennis & Chung-Lee, 2006; Giscombe et al., 2020; Jones et al., 2014; Sambrook Smith et al., 2019; Schmied et al., 2017; Slade et al., 2020; Sorsa et al., 2021; Viveiros & Darling, 2018 | 9 | Low confidence | Low confidence | Moderate confidence | Moderate confidence | **Moderate confidence** |
| **1.3.3 How to cope with symptoms** | | | | | | | | |
| 1.3.3.1 Ignore them | Women many deal with symptoms by ignoring them and assuming they will go away on their own | Bina, 2020; Hadfield & Wittkowski, 2017; Jones et al., 2014; Newman et al., 2019; Schmied et al., 2017; Slade et al., 2020 | 6 | Low confidence | Low confidence | Moderate confidence | Moderate confidence | **Moderate confidence** |
| 1.3.3.2 Seek spiritual guidance | Women may cope with symptoms by seeking spiritual guidance | Hansotte et al., 2017; Kassam, 2019; Schmied et al., 2017; Watson et al., 2019 | 4 | Moderate confidence | Very low confidence | High confidence | Moderate confidence | **Low confidence** |
| 1.3.3.3 Minimise them | Women may minimise or deny their symptoms | *Shakespeare et al., 2003*  Bina, 2020; Dennis & Chung-Lee, 2006; Forde et al., 2020; Hewitt et al., 2009; Holopainen & Hakulinen, 2019; Jones et al., 2014; Kassam, 2019; Megnin-Viggars et al., 2015; Schmied et al., 2017; Slade et al., 2020; Staneva et al., 2015; Tobin et al., 2018; Watson et al., 2019 | 14 | Moderate confidence | Low confidence | High confidence | Moderate confidence | **Moderate confidence** |
| **1.4 Deciding to seek help** | | | | | | | | |
| 1.4.1 Recognising something is wrong | The first step to seeking help for many women, was recognising that something is "wrong" | Bina, 2020; Button et al., 2017; Forde et al., 2020; Hadfield & Wittkowski, 2017; Slade et al., 2020; Staneva et al., 2015; Viveiros & Darling, 2018 | 8 | Moderate confidence | Low confidence | High confidence | Moderate confidence | **Moderate confidence** |
| 1.4.2 Where do I go to seek help? | The next step to help seeking is then understanding where to go in order to seek help | *Ganann et al., 2019*  Bina, 2020; Dennis & Chung-Lee, 2006; Hansotte et al., 2017; Megnin-Viggars et al., 2015; Schmied et al., 2017; Sorsa et al., 2021; Tobin et al., 2018 | 9 | Low confidence | Very low confidence | High confidence | Low confidence | **Low confidence** |
| **1.5 Fear of judgement** | | | | | | | | |
| 1.5.1 Fear of being seen as a bad mum | Fear of being judged and being seen as a bad mother | Bina, 2020; Brealey et al., 2010; Button et al., 2017; Forde et al., 2020; Jones et al., 2014; Lucas et al., 2019; Slade et al., 2020; Sorsa et al., 2021; Viveiros & Darling, 2018 | 9 | Low confidence | Low confidence | High confidence | High confidence | **Moderate confidence** |
| 1.5.2 Social services/ removal of child | Fear of social services involvement or their child being removed from their care | *Boyd et al., 2011; Feinberg et al., 2006; Shakespeare et al., 2003; Young et al., 2019*  Bina, 2020; Brealey et al., 2010; Button et al., 2017; Dennis & Chung-Lee, 2006; Evans et al., 2020; Forde et al., 2020; Hadfield & Wittkowski, 2017; Hewitt et al., 2009; A. Jones, 2019; Megnin-Viggars et al., 2015; Newman et al., 2019; Tobin et al., 2018; Watson et al., 2019  SH | 17 | Moderate confidence | Moderate confidence | High confidence | High confidence | **High confidence** |
| **1.6 Logistics of accessing perinatal mental health care** | | | | | | | | |
| 1.6.1 Childcare | Lack of childcare as a barrier to PMH Care | *Boyd et al., 2011; Cox et al., 2017; Doering et al., 2017; Friedman et al., 2010*  Bina, 2020; Button et al., 2017; Dennis & Chung-Lee, 2006; Hansotte et al., 2017; Morrell et al., 2016; Newman et al., 2019; Sambrook Smith et al., 2019; Scope et al., 2017; Watson et al., 2019 | 14 | Low confidence | Low confidence | High confidence | Low confidence | **Low confidence** |
| 1.6.2 Timing of care | Timing of appointments and services offered | *Atif et al., 2019; Friedman et al., 2010*  Bina, 2020; Dennis & Chung-Lee, 2006; Newman et al., 2019; Scope et al., 2017; Watson et al., 2019 | 7 | Low confidence | Low confidence | Moderate confidence | Low confidence | **Low confidence** |
| 1.6.3 Location/ travel | Location of services or travel costs to get to services | *Cox et al., 2017; Doering et al., 2017; Eappen et al., 2018; Friedman et al., 2010; Masood et al., 2015; Nakku et al., 2016*  Bina, 2020; Hansotte et al., 2017; Mollard et al., 2016; Morrell et al., 2016; Newman et al., 2019; Tobin et al., 2018; Watson et al., 2019 | 13 | Moderate confidence | Low confidence | High confidence | Low confidence | **Low confidence** |
| **1.7 Social and family life** | | | | | | | | |
| 1.7.1 Social isolation or support | Woman’s experiences of social support or social isolation | Giscombe et al., 2020; Hansotte et al., 2017; A. Jones, 2019; C. Jones et al., 2014; Kassam, 2019; Lucas et al., 2019; Tobin et al., 2018; Viveiros & Darling, 2018; Watson et al., 2019 | 9 | Low confidence | Low confidence | Moderate confidence | Moderate confidence | **Moderate confidence** |
| 1.7.2 Family and friends’ beliefs | Woman’s family and friends’ beliefs about mental illness | *Atif et al., 2016, 2019; Boyd et al., 2011; Doering et al., 2017; Ganann et al., 2019; Higgins et al., 2018; Masood et al., 2015; Nakku et al., 2016; Nithianandan et al., 2016; Noonan et al., 2018; O’Mahen & Flynn, 2008; Pineros-Leano et al., 2015; Vik et al., 2009; Williams et al., 2016; Young et al., 2019*  Bina, 2020; Button et al., 2017; Dennis & Chung-Lee, 2006; Forde et al., 2020; Hadfield & Wittkowski, 2017; Holopainen & Hakulinen, 2019; A. Jones, 2019; Lucas et al., 2019; Nilaweera et al., 2014; Sambrook Smith et al., 2019; Schmied et al., 2017; Sorsa et al., 2021; Viveiros & Darling, 2018; Watson et al., 2019 | 30 | Moderate confidence | High confidence | Moderate confidence | Moderate confidence | **Moderate confidence** |
| 1.7.3 Additional personal difficulties | Personal difficulties outside of PMH such as unemployment | *Atif et al., 2016; Boyd et al., 2011; Kerker et al., 2018; Munodawafa et al., 2017; Rowan et al., 2010; Williams et al., 2016*  Hansotte et al., 2017 | 7 | Moderate confidence | Low confidence | Moderate confidence | Moderate confidence | **Moderate confidence** |
| **1.8 Sociodemographic factors** | | | | | | | | |
| 1.8.1 Ethnicity | Woman’s ethnicity | Bina, 2020; Dennis & Chung-Lee, 2006; Hansotte et al., 2017; Watson et al., 2019 | 4 | Low confidence | Very low confidence | High confidence | Moderate confidence | **Low confidence** |
| 1.8.2 Age | Woman’s age | Bina, 2020; Hansotte et al., 2017 | 2 | Low confidence | Very low confidence | High confidence | Very low confidence | **Very low confidence** |
| **1.9 Mental health factors** | | | | | | | | |
| 1.9.1 Previous experiences of mental health care | Previous experiences of mental health care | *O’Mahen & Flynn, 2008*  Button et al., 2017; Evans et al., 2020; Hadfield & Wittkowski, 2017; Hansotte et al., 2017; Watson et al., 2019 | 6 | Moderate confidence | Low confidence | High confidence | Moderate confidence | **Moderate confidence** |
| 1.9.2 Previous diagnoses or symptoms | Previous experiences of mental health symptoms or diagnoses | Bina, 2020; Sorsa et al., 2021 | 2 | Low confidence | Very low confidence | High confidence | Very low confidence | **Very low confidence** |
| 1.9.3 Current diagnoses or symptoms | Current experiences of mental health symptoms or diagnoses | *Chartier et al., 2015; Friedman et al., 2010; Hadfield et al., 2019; Young et al., 2019*  Sorsa et al., 2021; Viveiros & Darling, 2018 | 6 | Low confidence | Low confidence | High confidence | Moderate confidence | **Low confidence** |
| **2. HCPs** | | | | | | | | |
| **2.1 HCPs knowledge about PMH** | | | | | | | | |
| 2.1.1 HCPs knowledge about PMI | HCPs knowledge about PMH actual & perceived by women | *Beeber et al., 2009; Byatt et al., 2013; Ganann et al., 2019; Higgins et al., 2018; Judd et al., 2011; McCauley et al., 2019; McKenzie-McHarg et al., 2014; Noonan et al., 2018; Reed et al., 2014; Rowan et al., 2010*  Bina, 2020; Dennis & Chung-Lee, 2006; Megnin-Viggars et al., 2015; Morrell et al., 2016; Slade et al., 2020; Viveiros & Darling, 2018  SH | 17 | Moderate confidence | Moderate confidence | High confidence | Moderate confidence | **Moderate confidence** |
| 2.1.2 HCP’s knowledge about services/referral pathways | HCPs knowledge about PMH services and referral pathways actual & perceived by women | *Ganann et al., 2019; Higgins et al., 2018; Rowan et al., 2010*  Dennis & Chung-Lee, 2006; Hansotte et al., 2017; Sambrook Smith et al., 2019; Slade et al., 2020; Viveiros & Darling, 2018 | 8 | Moderate confidence | Low confidence | High confidence | High confidence | **High confidence** |
| 2.1.3 HCPs confidence | HCP's confidence in addressing PMH | *Atif et al., 2019; Bina et al., 2018; Cox et al., 2017; Fernandez y Garcia et al., 2011; Higgins et al., 2018; Munodawafa et al., 2017; Nithianandan et al., 2016; Ormsby et al., 2018; Reed et al., 2014*  SH | 9 | Moderate confidence confidence | Low confidence | High confidence | Moderate confidence | **Moderate confidence** |
| **2.2 Getting it right the first time** | | | | | | | | |
| 2.2.1 Being dismissive or normalising symptoms | HCPs dismissing or normalising symptoms | *Ganann et al., 2019*  Button et al., 2017; Dennis & Chung-Lee, 2006; Forde et al., 2020; Hadfield & Wittkowski, 2017; Hansotte et al., 2017; Megnin-Viggars et al., 2015; Newman et al., 2019; Sorsa et al., 2021; Watson et al., 2019  SH | 11 | Low confidence | Low confidence | High confidence | High confidence | **High confidence** |
| 2.2.2 Not recognising help seeking or PMI | HCPs not recognising help seeking or PMI | Bina, 2020; Button et al., 2017; Megnin-Viggars et al., 2015; Tobin et al., 2018; Watson et al., 2019 | 5 | Low confidence | Very low confidence | High confidence | Moderate confidence | **Moderate confidence** |
| 2.2.3 Focussing on infant | HCPs focussing mainly on the infant | Button et al., 2017; Megnin-Viggars et al., 2015 | 2 | Low confidence | Very low confidence | High confidence | Moderate confidence | **Low confidence** |
| 2.2.4 Making time | A HCP who makes time to address PMH concerns | *Feinberg et al., 2006; Myors et al., 2015; Noonan et al., 2018*  Bina, 2020; Button et al., 2017; Dennis & Chung-Lee, 2006; Hewitt et al., 2009; Megnin-Viggars et al., 2015; Slade et al., 2020; Viveiros & Darling, 2018; Watson et al., 2019 | 11 | Moderate confidence | Low confidence | Moderate confidence | High confidence | **Moderate confidence** |
| 2.2.5 Assessment specific behaviours | HCP's assessment specific behaviours, such as asking about PMH, carrying out in a tick box way, or in a personalised way | *Doering et al., 2017; Fernandez y Garcia et al., 2011; Nithianandan et al., 2016; Segre et al., 2014; Vik et al., 2009; Williams et al., 2016*  Brealey et al., 2010; Sambrook Smith et al., 2019; Schmied et al., 2017; Slade et al., 2020; Viveiros & Darling, 2018; Watson et al., 2019 | 12 | Moderate confidence | Low confidence | Moderate confidence | Moderate confidence | **Moderate confidence** |
| **2.3 HCPs’ attributes** | | | | | | | | |
| 2.3.1 Similar demographic characteristics | HCP having similar demographics to women | *Leger et al., 2015; Masood et al., 2015; Nithianandan et al., 2016; Shorey & Ng, 2019*  Dennis & Chung-Lee, 2006; Watson et al., 2019 | 6 | Moderate confidence | Low confidence | High confidence | Moderate confidence | **Moderate confidence** |
| 2.3.2 Culturally sensitive | HCP being sensitive to women from all cultures | Kassam, 2019; Nilaweera et al., 2014; Viveiros & Darling, 2018; Watson et al., 2019*;* | 4 | Moderate confidence | Very low confidence | Moderate confidence | Moderate confidence | **Low confidence** |
| 2.3.3 Valued characteristics | HCPs possessing valued characteristics Trustworthy, empathetic, kind, caring with a genuinine interest, and going above and beyond | *Atif et al., 2016, 2019; Boyd et al., 2011; Doering et al., 2017; Kerker et al., 2018; Kim et al., 2009; Munodawafa et al., 2017; Myors et al., 2015; Pugh et al., 2015; Shorey & Ng, 2019*  Brealey et al., 2010; Button et al., 2017; Dennis & Chung-Lee, 2006; Forde et al., 2020; Hadfield & Wittkowski, 2017; Hewitt et al., 2009; Jones, 2019; Megnin-Viggars et al., 2015; Morrell et al., 2016; Newman et al., 2019; Schmied et al., 2017; Slade et al., 2020; Staneva et al., 2015; Watson et al., 2019  SH | 25 | Moderate confidence | High confidence | High confidence | High confidence | **High confidence** |
| **3. Interpersonal** | | | | | | | | |
| 3.1 Trusting relationship and rapport | The development of a trusting relationship and rapport between HCP and women | *Atif et al., 2016; Doering et al., 2017; Feinberg et al., 2006; Ganann et al., 2019; Hadfield et al., 2019; Higgins et al., 2018; Kerker et al., 2018; Leger et al., 2015; Noonan et al., 2018; Shakespeare et al., 2003; Shorey & Ng, 2019; Willey et al., 2018; Williams et al., 2016; Young et al., 2019*  Bina, 2020; Brealey et al., 2010; Dennis & Chung-Lee, 2006; Hadfield & Wittkowski, 2017; Hewitt et al., 2009; Megnin-Viggars et al., 2015; Morrell et al., 2016; Scope et al., 2017; Tobin et al., 2018 | 23 | Moderate confidence | High confidence | Moderate confidence | High confidence | **High confidence** |
| 3.2 Language barriers | Difficulties in communicating due to language barriers | *Beeber et al., 2009; Doering et al., 2017; Ganann et al., 2019; Masood et al., 2015; Munodawafa et al., 2017; Nithianandan et al., 2016; Pineros-Leano et al., 2015; Segre et al., 2014; Willey et al., 2018; Williams et al., 2016*  Dennis & Chung-Lee, 2006; Hansotte et al., 2017; Megnin-Viggars et al., 2015; Sambrook Smith et al., 2019; Schmied et al., 2017; Watson et al., 2019  SH | 16 | Moderate confidence | Moderate confidence | High confidence | High confidence | **High confidence** |
| 3.3 Shared decision making | Shared decision making between HCP and woman | Hadfield & Wittkowski, 2017; Megnin-Viggars et al., 2015; Randall & Briscoe, 2018; Scope et al., 2017 | 4 | Low confidence | Very low confidence | Moderate confidence | Moderate confidence | **Low confidence** |
| 3.4 Open and honest communication | Open and honest communication between HCP and woman | *Doering et al., 2017; Shakespeare et al., 2003; Vik et al., 2009; Willey et al., 2018*  Brealey et al., 2010; Hadfield & Wittkowski, 2017; Hewitt et al., 2009; Schmied et al., 2017; Watson et al., 2019  SH | 9 | Moderate confidence | Low confidence | Moderate confidence | High confidence | **Moderate confidence** |
| **4. Organisational** | | | | | | | | |
| **4.1 Overall organisational aspects** | | | | | | | | |
| 4.1.1 Co location and buildings | Location of the service including co-location of different services within the same building | *Boyd et al., 2011; Cox et al., 2017; Judd et al., 2011; Munodawafa et al., 2017; Ormsby et al., 2018; Young et al., 2019;*  Bina, 2020  SH | 7 | Moderate confidence | Low confidence | Moderate confidence | Low confidence | **Low confidence** |
| 4.1.2 Service integration and collaborative working | Collaborative working across services  SH | *Atif et al., 2016; Bina et al., 2018; Boyd et al., 2011; Byatt et al., 2013; Feinberg et al., 2006; Ganann et al., 2019; Hadfield et al., 2019; Judd et al., 2011; Lind et al., 2017; Lomonaco-Haycraft et al., 2018; Myors et al., 2015; Nithianandan et al., 2016; Noonan et al., 2018; Rowan et al., 2010*  Newman et al., 2019; Sambrook Smith et al., 2019; Watson et al., 2019 | 17 | Moderate confidence | Moderate confidence | Moderate confidence | Moderate confidence | **Moderate confidence** |
| 4.1.3 Collaboration within services | Collaborative working within services  SH | *Ammerman et al., 2014; Cox et al., 2017; Eappen et al., 2018; Higgins et al., 2018; Judd et al., 2011; Kerker et al., 2018; Lind et al., 2017; McKenzie-McHarg et al., 2014; Munodawafa et al., 2017; Nithianandan et al., 2016; Ormsby et al., 2018; Segre et al., 2014; Willey et al., 2018*  Sambrook Smith et al., 2019 | 14 | Moderate confidence | Moderate confidence | Moderate confidence | Moderate confidence | **Moderate confidence** |
| 4.1.4 Adequate workforce provision/ HCPs workload | Ensuring and adequate workforce provision so PMH can be addressed | *Ammerman et al., 2014; Bina et al., 2018; Drozd et al., 2018; Feinberg et al., 2006; Ganann et al., 2019; Higgins et al., 2018; Kerker et al., 2018; Kim et al., 2009; McCauley et al., 2019; Nakku et al., 2016; Nithianandan et al., 2016; Noonan et al., 2018; Rowan et al., 2010; Vik et al., 2009; Willey et al., 2018*  Bina, 2020; Viveiros & Darling, 2018  SH | 17 | Moderate confidence | Moderate confidence | High confidence | High confidence | **High confidence** |
| 4.1.5 Clear assessment and referral process | Clear assessment and referral processes within the organisation | *Cox et al., 2017; Feinberg et al., 2006; Ganann et al., 2019; Judd et al., 2011; Kerker et al., 2018; Kim et al., 2009; Nithianandan et al., 2016; Rowan et al., 2010; Segre et al., 2014; Williams et al., 2016*  Bina, 2020  SH | 11 | Moderate confidence | Low confidence | Moderate confidence | Moderate confidence | **Moderate confidence** |
| 4.1.6 Provision of supervision | Supervision for HCPs | *Atif et al., 2019; Munodawafa et al., 2017; Vik et al., 2009* | 3 | Low confidence | Very low confidence | High confidence | Very low confidence | **Very low confidence** |
| 4.1.7 Training | Provision of training for all HCPs working with perinatal women | *Ammerman et al., 2014; Atif et al., 2016, 2019; Beeber et al., 2009; Bina et al., 2018; Boyd et al., 2011; Chartier et al., 2015; Doering et al., 2017; Drozd et al., 2018; Feinberg et al., 2006; Ganann et al., 2019; Judd et al., 2011; Kerker et al., 2018; Kim et al., 2009; Leger et al., 2015; Lind et al., 2017; McKenzie-McHarg et al., 2014; Munodawafa et al., 2017; Nakku et al., 2016; Nithianandan et al., 2016; Noonan et al., 2018; Reed et al., 2014; Rowan et al., 2010; Shorey & Ng, 2019; Willey et al., 2018; Williams et al., 2016*  Bina, 2020; Brealey et al., 2010  SH | 28 | Moderate confidence | High confidence | High confidence | High confidence | **High confidence** |
| 4.1.9 Organisational goals/guidelines | Clear organisational goals and guidelines | *Ammerman et al., 2014; Willey et al., 2018* | 2 | Low confidence | Very low confidence | Very low confidence | Very low confidence | **Very low confidence** |
| **4.2 Characteristics of PMH Care** | | | | | | | | |
| **4.2.1 Across the care pathway** | | | | | | | | |
| 4.2.1.1 Continuity of carer | Care that provides the same HCP along the care pathway | *Chartier et al., 2015; Higgins et al., 2018; Nithianandan et al., 2016; O’Mahen & Flynn, 2008; Rowan et al., 2010; Willey et al., 2018*  Brealey et al., 2010; Button et al., 2017; Dennis & Chung-Lee, 2006; Hadfield & Wittkowski, 2017; Megnin-Viggars et al., 2015; Sambrook Smith et al., 2019; Slade et al., 2020; Tobin et al., 2018; Viveiros & Darling, 2018; Watson et al., 2019  SH | 17 | Moderate confidence | Moderate confidence | High confidence | High confidence | **High confidence** |
| 4.2.1.2 Culturally sensitive care | Care that is culturally sensitive to women's needs | *Ganann et al., 2019; Nithianandan et al., 2016; Noonan et al., 2018; Shorey & Ng, 2019*  Bina, 2020; Brealey et al., 2010; Button et al., 2017; Dennis & Chung-Lee, 2006; Giscombe et al., 2020; Hadfield & Wittkowski, 2017; Hansotte et al., 2017; Hewitt et al., 2009; Jones, 2019; Kassam, 2019; Sambrook Smith et al., 2019; Schmied et al., 2017; Tobin et al., 2018; Viveiros & Darling, 2018; Watson et al., 2019 | 19 | Moderate confidence | Moderate confidence | High confidence | High confidence | **High confidence** |
| 4.2.1.3 Privacy & confidentiality | Care that is private and maintains women's confidentiality | *Atif et al., 2019; Feinberg et al., 2006; Higgins et al., 2018; Jallo et al., 2015; Nithianandan et al., 2016; O’Mahen & Flynn, 2008*  Giscombe et al., 2020 | 7 | Moderate confidence | Low confidence | Moderate confidence | Low confidence | **Low confidence** |
| 4.2.1.4 Dedicated person/ PMH Champion | Care that has a dedicated person or PMH champion | *Chartier et al., 2015; Ganann et al., 2019; Kim et al., 2009; Lomonaco-Haycraft et al., 2018; Nithianandan et al., 2016; Rowan et al., 2010; Willey et al., 2018* Bina, 2020; Megnin-Viggars et al., 2015 | 9 | Low confidence | Low confidence | Moderate confidence | Low confidence | **Low confidence** |
| 4.2.1.5 Logistical support | Logistical support for women including easily accessible location, childcare, travel costs | *Ganann et al., 2019; Hadfield & Wittkowski, 2017; Leger et al., 2015; Masood et al., 2015; Nakku et al., 2016; Nithianandan et al., 2016; Ormsby et al., 2018*  Button et al., 2017; Jones, 2019; Mollard et al., 2016; Newman et al., 2019; Scope et al., 2017; Watson et al., 2019 | 13 | Moderate confidence | Low confidence | High confidence | Moderate confidence | **Moderate confidence** |
| 4.2.1.6 Home delivery | Care that is delivered at home | *Ammerman et al., 2014; Beeber et al., 2009; Judd et al., 2011; Leger et al., 2015; Munodawafa et al., 2017; Myors et al., 2015*  Brealey et al., 2010; Hadfield & Wittkowski, 2017; Hansotte et al., 2017; Jones, 2019 | 10 | Moderate confidence | Low confidence | High confidence | Moderate confidence | **Moderate confidence** |
| 4.2.1.7 Hospital delivery | Care that is delivered in hopsital/medical setting | *Atif et al., 2019; Boyd et al., 2011; Kerker et al., 2018; Shakespeare et al., 2003*  Dennis & Chung-Lee, 2006 | 5 | Low confidence | Very low confidence | Low confidence | Low confidence | **Low confidence** |
| 4.2.1.8 Provision of information | Whether care provides information | Dennis & Chung-Lee, 2006; Hadfield & Wittkowski, 2017; Jones, 2019; Megnin-Viggars et al., 2015; Morrell et al., 2016; Randall & Briscoe, 2018; Schmied et al., 2017 | 7 | Low confidence | Low confidence | Moderate confidence | Moderate confidence | **Moderate confidence** |
| 4.2.1.9 Technology | The use of technology in care | *Doering et al., 2017; Feinberg et al., 2006; Fernandez y Garcia et al., 2011; Jallo et al., 2015; Kim et al., 2009; Lind et al., 2017; Noonan et al., 2018; Pineros-Leano et al., 2015; Shorey & Ng, 2019; Willey et al., 2018; Williams et al., 2016*  SH | 11 | Moderate confidence | Low confidence | High confidence | High confidence | **High confidence** |
| 4.2.1.10 Service inclusion criteria | Inclusion criteria of services | *Boyd et al., 2011; Ganann et al., 2019*  Viveiros & Darling, 2018 | 3 | Moderate confidence | Very low confidence | Moderate confidence | Low confidence | **Low confidence** |
| **4.2.2 Assessment specific characteristics** | | | | | | | | |
| 4.2.2.1 Wording of assessment tools | How assessment tools are worded | *Doering et al., 2017; Segre et al., 2014; Williams et al., 2016*  Brealey et al., 2010; Button et al., 2017; Hewitt et al., 2009 | 6 | Moderate confidence | Low confidence | High confidence | High confidence | **Moderate confidence** |
| 4.2.2.2 Acceptability of assessment/ screening | Whether assessment tools and assessment/screening in general are acceptable to women and HCPs | *Boyd et al., 2011; Doering et al., 2017; Feinberg et al., 2006; Ganann et al., 2019; Kim et al., 2009; Nithianandan et al., 2016; Segre et al., 2014; Shakespeare et al., 2003; Vik et al., 2009; Willey et al., 2018*  Brealey et al., 2010; Evans et al., 2020; Hewitt et al., 2009; Megnin-Viggars et al., 2015; Mollard et al., 2016; Sambrook Smith et al., 2019; Viveiros & Darling, 2018 | 17 | Moderate confidence | Moderate confidence | Moderate confidence | Moderate confidence | **Moderate confidence** |
| **4.2.3 Intervention characteristics** | | | | | | | | |
| 4.2.3.1 Opportunity to talk | Interventions that provide an opportunity to talk | Dennis & Chung-Lee, 2006; Evans et al., 2020; Hadfield & Wittkowski, 2017; Jones et al., 2014; Kassam, 2019; Morrell et al., 2016; Praetorius et al., 2020 | 7 | Low confidence | Low confidence | High confidence | Low confidence | **Low confidence** |
| 4.2.3.2 Individualised and person centred | Indiviudalised and person centred interventions/care | *Chartier et al., 2015; Doering et al., 2017; Ganann et al., 2019; Masood et al., 2015; McKenzie-McHarg et al., 2014; Noonan et al., 2018; O’Mahen & Flynn, 2008; Pugh et al., 2015; Segre et al., 2014; Shorey & Ng, 2019*  Evans et al., 2020; Hadfield & Wittkowski, 2017; Megnin-Viggars et al., 2015; Morrell et al., 2016; Schmied et al., 2017; Scope et al., 2017; Slade et al., 2020; Watson et al., 2019  SH | 19 | Moderate confidence | Moderate confidence | Moderate confidence | High confidence | **Moderate confidence** |
| 4.2.3.3 Appropriateness | Appropriateness of intervention being offered, from women and HCPs point of view | *Atif et al., 2019; Bina et al., 2018; Chartier et al., 2015; Drozd et al., 2018; Leger et al., 2015; McKenzie-McHarg et al., 2014; Munodawafa et al., 2017; Noonan et al., 2018; Ormsby et al., 2018; Pugh et al., 2015; Reed et al., 2014; Shorey & Ng, 2019*  Evans et al., 2020; Megnin-Viggars et al., 2015; Scope et al., 2017 | 15 | Moderate confidence | Moderate confidence | High confidence | Low confidence | **Moderate confidence** |
| 4.2.3.4 Flexible | Flexibility of intervention | *Atif et al., 2019; Bina et al., 2018; Ganann et al., 2019; Hadfield et al., 2019; Judd et al., 2011; Munodawafa et al., 2017; O’Mahen & Flynn, 2008; Pugh et al., 2015; Shorey & Ng, 2019*  Sorsa et al., 2021; Watson et al., 2019 | 11 | Moderate confidence | Low confidence | High confidence | Moderate confidence | **Moderate confidence** |
| 4.2.3.5 Group support | Group/peer support as an intervention | *Hadfield et al., 2019; Masood et al., 2015; Nakku et al., 2016*  Dennis & Chung-Lee, 2006; Evans et al., 2020; Hadfield & Wittkowski, 2017; Holopainen & Hakulinen, 2019; Jones et al., 2014; Megnin-Viggars et al., 2015; Schmied et al., 2017; Scope et al., 2017; Slade et al., 2020; Tobin et al., 2018; Watson et al., 2019  SH | 14 | Moderate confidence | Low confidence | Moderate confidence | High confidence | **Moderate confidence** |
| 4.2.3.7 Face to face delivery | Face to face delivery of intervention | *O’Mahen & Flynn, 2008; Pugh et al., 2015; Shorey & Ng, 2019*  Schmied et al., 2017  SH | 4 | Moderate confidence | Very low confidence | High confidence | High confidence | **Low confidence** |
| **5. Commissioners** | | | | | | | | |
| 5.1 Referral pathways | Clear referral pathways | *Ammerman et al., 2014; Boyd et al., 2011; Higgins et al., 2018; Nithianandan et al., 2016; Rowan et al., 2010; Willey et al., 2018*  SH | 6 | Moderate confidence | Low confidence | Moderate confidence | High confidence | **Moderate confidence** |
| 5.2 Lack of appropriate or timely services | Lack of appropriate and timely services to refer women onto | *Boyd et al., 2011; Doering et al., 2017; Ganann et al., 2019; Higgins et al., 2018; Kerker et al., 2018; Leger et al., 2015; Lomonaco-Haycraft et al., 2018; Munodawafa et al., 2017; Myors et al., 2015; Nakku et al., 2016; Noonan et al., 2018; Rowan et al., 2010; Williams et al., 2016*  Bina, 2020; Button et al., 2017; Jones, 2019; Jones et al., 2014; Megnin-Viggars et al., 2015; Newman et al., 2019; Sambrook Smith et al., 2019; Tobin et al., 2018; Viveiros & Darling, 2018  SH | 22 | Moderate confidence | High confidence | High confidence | High confidence | **High confidence** |
| 5.3 Financial complexities | Financial complexities including funding, and sourcing money and resources for services and financial reimbursement | *Cox et al., 2017; Feinberg et al., 2006; Friedman et al., 2010; Ganann et al., 2019; Kim et al., 2009; Lomonaco-Haycraft et al., 2018; Ormsby et al., 2018; Rowan et al., 2010*  SH | 8 | Moderate confidence | Low confidence | Moderate confidence | Moderate confidence | **Moderate confidence** |
| **6. Political** | | | | | | | | |
| 6.1 Immigration status | How the immigration status of women may impact their PMH care journey | *Cox et al., 2017; Ganann et al., 2019*  Bina, 2020; Giscombe et al., 2020; Hansotte et al., 2017; Kassam, 2019; Schmied et al., 2017; Tobin et al., 2018; Watson et al., 2019  SH | 9 | Moderate confidence | Low confidence | High confidence | High confidence | **High confidence** |
| 5.2 Economic status & healthcare costs | How the cost of healthcare, and women's economic status may impact their PMH care journey | *Atif et al., 2016; Boyd et al., 2011; Cox et al., 2017; Doering et al., 2017; Ganann et al., 2019; Lomonaco-Haycraft et al., 2018; Munodawafa et al., 2017; Nakku et al., 2016; Ormsby et al., 2018*  Bina, 2020; Hansotte et al., 2017; Jones, 2019; Kassam, 2019; Lucas et al., 2019; Tobin et al., 2018; Viveiros & Darling, 2018  SH | 16 | Moderate confidence | Moderate confidence | High confidence | Low confidence | **Moderate confidence** |
| **7. Societal** | | | | | | | | |
| 7.1 Stigma | Stigma related to mental illness | *Atif et al., 2016, 2019; Boyd et al., 2011; Chartier et al., 2015; Cox et al., 2017; Feinberg et al., 2006; Higgins et al., 2018; Kerker et al., 2018; McCauley et al., 2019; McKenzie-McHarg et al., 2014; Munodawafa et al., 2017; Myors et al., 2015; Nakku et al., 2016; Nithianandan et al., 2016; Noonan et al., 2018; O’Mahen & Flynn, 2008; Shakespeare et al., 2003; Vik et al., 2009; Williams et al., 2016; Young et al., 2019*  Bina et al., 2018; Button et al., 2017; Dennis & Chung-Lee, 2006; Giscombe et al., 2020; Hadfield & Wittkowski, 2017; Hansotte et al., 2017; Hewitt et al., 2009; Holopainen & Hakulinen, 2019; A. Jones, 2019; Kassam, 2019; Lucas et al., 2019; Megnin-Viggars et al., 2015; Mollard et al., 2016; Morrell et al., 2016; Nilaweera et al., 2014; Sambrook Smith et al., 2019; Schmied et al., 2017; Scope et al., 2017; Sorsa et al., 2021; Tobin et al., 2018; Viveiros & Darling, 2018; Watson et al., 2019  SH | 43 | Moderate confidence | High confidence | High confidence | High confidence | **High confidence** |
| 7.2 Culture | Cultural beliefs about mental illness and seeking and accessing help | *Atif et al., 2016; Bina et al., 2018; Boyd et al., 2011; Feinberg et al., 2006; Friedman et al., 2010; Ganann et al., 2019; Higgins et al., 2018; Masood et al., 2015; McCauley et al., 2019; Nakku et al., 2016; Noonan et al., 2018; Segre et al., 2014*  Brealey et al., 2010; Button et al., 2017; Dennis & Chung-Lee, 2006; Giscombe et al., 2020; Hansotte et al., 2017; Hewitt et al., 2009; Holopainen & Hakulinen, 2019; Jones, 2019; Kassam, 2019; Megnin-Viggars et al., 2015; Praetorius et al., 2020; Sambrook Smith et al., 2019; Schmied et al., 2017; Staneva et al., 2015; Tobin et al., 2018; Viveiros & Darling, 2018; Watson et al., 2019; Wittkowski et al., 2014  SH | 30 | Moderate confidence | High confidence | High confidence | High confidence | **High confidence** |
| 7.3 Maternal norms | Maternal norms of being a "good mother" and a "strong woman" | *Shakespeare et al., 2003; Williams et al., 2016*  Bina, 2020; Brealey et al., 2010; Button et al., 2017; Dennis & Chung-Lee, 2006; Hadfield & Wittkowski, 2017; Hansotte et al., 2017; Hewitt et al., 2009; Holopainen & Hakulinen, 2019; Jones et al., 2014; Kassam, 2019; Lucas et al., 2019; Megnin-Viggars et al., 2015; Mollard et al., 2016; Newman et al., 2019; Nilaweera et al., 2014; Praetorius et al., 2020; Sambrook Smith et al., 2019; Schmied et al., 2017; Slade et al., 2020; Sorsa et al., 2021; Staneva et al., 2015; Viveiros & Darling, 2018; Watson et al., 2019  SH | 26 | Low confidence | High confidence | High confidence | High confidence | **High confidence** |

# *Supplementary Materials 10:* Identified barriers and facilitators to perinatal mental health care

| **#** | **Barriers** | **#** | **Facilitators** |
| --- | --- | --- | --- |
| *1* | *Assessment viewed as not acceptable by women and HPs* | *1* | *Assessment viewed as acceptable by women and HPs* |
| *2* | *Care provision lacks privacy and confidentiality* | *2* | *Care offered in undisturbed settings that ensure privacy* |
| *3* | *Care that does not provide logistical support* | *3* | *Care provides logistical support* |
| *4* | *Care that is not appropriate to women’s needs* | *4* | *Care that is appropriate to women’s needs* |
| *5* | *Care that is not delivered in a home setting* | *5* | *Home delivery of care* |
| *6* | *Care that lacks cultural sensitivity* | *6* | *Culturally sensitive care* |
| *7* | *Confusing organisational referral and assessment processes* | *7* | *Clear organisational assessment and referral processes* |
| *8* | *HPs not making time to address PMH difficulties* | *8* | *HPs making time to address PMH* |
| *9* | *HPs carrying out assessment in an impersonal way (e.g., tick box exercise)* | *9* | *HPs carrying out assessment in a personalised way* |
| *10* | *HPs having a poor knowledge about PMI* | *10* | *HPs having a good knowledge of PMH* |
| *11* | *HPs having a poor knowledge about services* | *11* | *HPs having a good knowledge of services and pathways* |
| *12* | *HPs having low confidence about addressing PMH* | *12* | *HPs having high confidence about addressing PMH* |
| *13* | *HPs not having received adequate PMH training* | *13* | *HPs having received adequate PMH training* |
| *14* | *HPs workload too heavy due to inadequate workforce provision* | *14* | *Adequate workforce provision to meet women’s needs* |
| *15* | *Inflexible care* | *15* | *Flexible care* |
| *16* | *Issues with technology* | *16* | *Technology that is fit for purpose* |
| *17* | *Lack of collaboration between services* | *17* | *Collaboration between services* |
| *18* | *Lack of information provision about care and PMH* | *18* | *Care provides information about PMI and available services* |
| *19* | *Lack of support from family and friends* | *19* | *Supportive family and friends* |
| *20* | *No continuity of carer* | *20* | *Continuity of carer* |
| *21* | *No open honest communication between women and HPs* | *21* | *Open and honest communication* |
| *22* | *No relationship and rapport between women and HPs* | *22* | *Trusting relationship between women and HPs* |
| *23* | *No shared decision making between women and HPs* | *23* | *Shared decision making between women and HPs* |
| *24* | *Staff within services not working together* | *24* | *Collaboration within services* |
| *25* | *Women being socially isolated* | *25* | *Women’s social support network* |
| *26* | *Women viewing peer support as not an acceptable intervention* | *26* | *Women finding group support acceptable* |
| *27* | *Women's previous negative experiences of mental health services* | *27* | *Women’s previous positive experiences of mental health services* |
| 28 | Women being an immigrant or a refugee | 28 | Care that offers an opportunity to talk |
| 29 | Care that is carried out in medical setting | 29 | Champion/dedicated PMH person |
| 30 | Complicated or confusing wording of assessment tools | 30 | Clear goals and guidelines |
| 31 | Women’s belief that services are too complex or complicated – difficult to navigate | 31 | Clear referral pathways |
| 32 | Cultural barriers | 32 | Co-location of services |
| 33 | Current symptoms getting in the way of treatment | 33 | Face to face care |
| 34 | Funding complexities | 34 | HPs receiving supervision |
| 35 | HPs dismissive or normalising symptoms | 35 | Individualised person-centred care |
| 36 | HPs focusing only on infant | 36 | Previous mental health diagnoses/symptoms |
| 37 | HPs lacking cross cultural knowledge of PMH | 37 | Recognising something is wrong |
| 38 | HPs not being interested in PMI | 38 | Valued characteristics of HPs |
| 39 | HPs not recognising help seeking | 39 | Women and HPs having similar demographics |
| 40 | Lack of appropriate PMH services |  |  |
| 41 | Lack of childcare |  |  |
| 42 | Language barriers |  |  |
| 43 | Maternal norms of being a "good mother" and a "strong" woman |  |  |
| 44 | Mother's worries about being judged as a "bad mum" |  |  |
| 45 | Restrictive eligibility criteria of care |  |  |
| 46 | Stigma |  |  |
| 47 | Stretched services |  |  |
| 48 | The belief that services only offer medication |  |  |
| 49 | Timing of services not suitable to women's needs |  |  |
| 50 | Travel costs |  |  |
| 51 | Women’s additional personal difficulties |  |  |
| 52 | Women being from an ethnic minority |  |  |
| 53 | Women being on a low income |  |  |
| 54 | Women being worried about social services involvement |  |  |
| 55 | Women being younger |  |  |
| 56 | Women believing PMH symptoms are a normal part of motherhood |  |  |

*Note*. Italics = both barriers and facilitators

# *Supplementary Materials 11:* MATRIx barriers conceptual framework


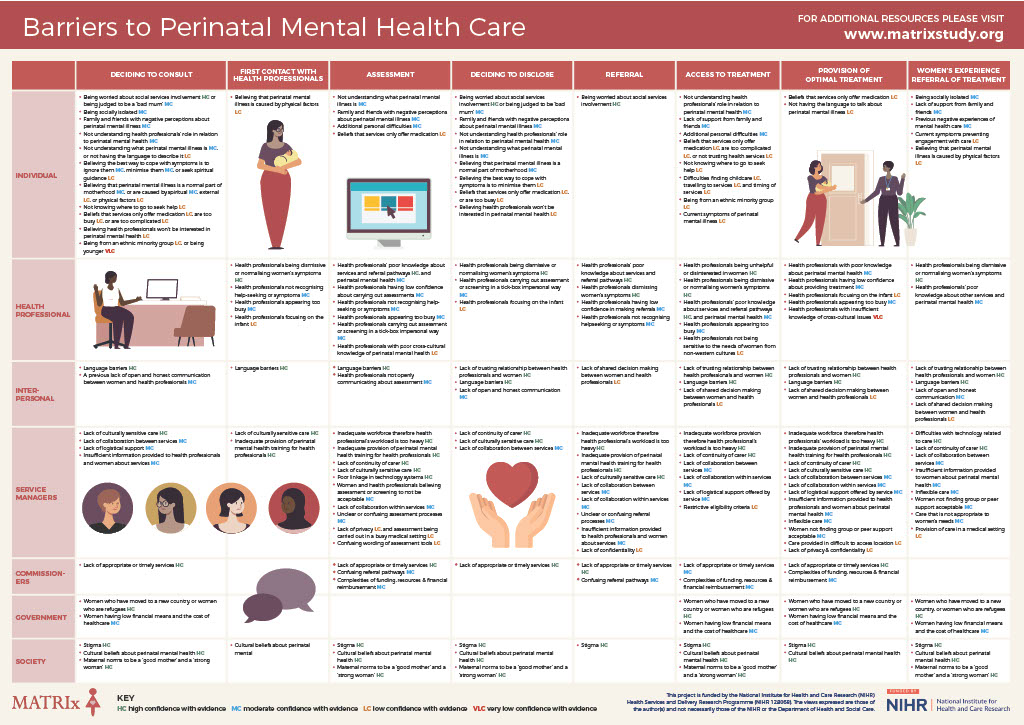


# *Supplementary Materials 12:* MATRIx facilitators conceptual framework


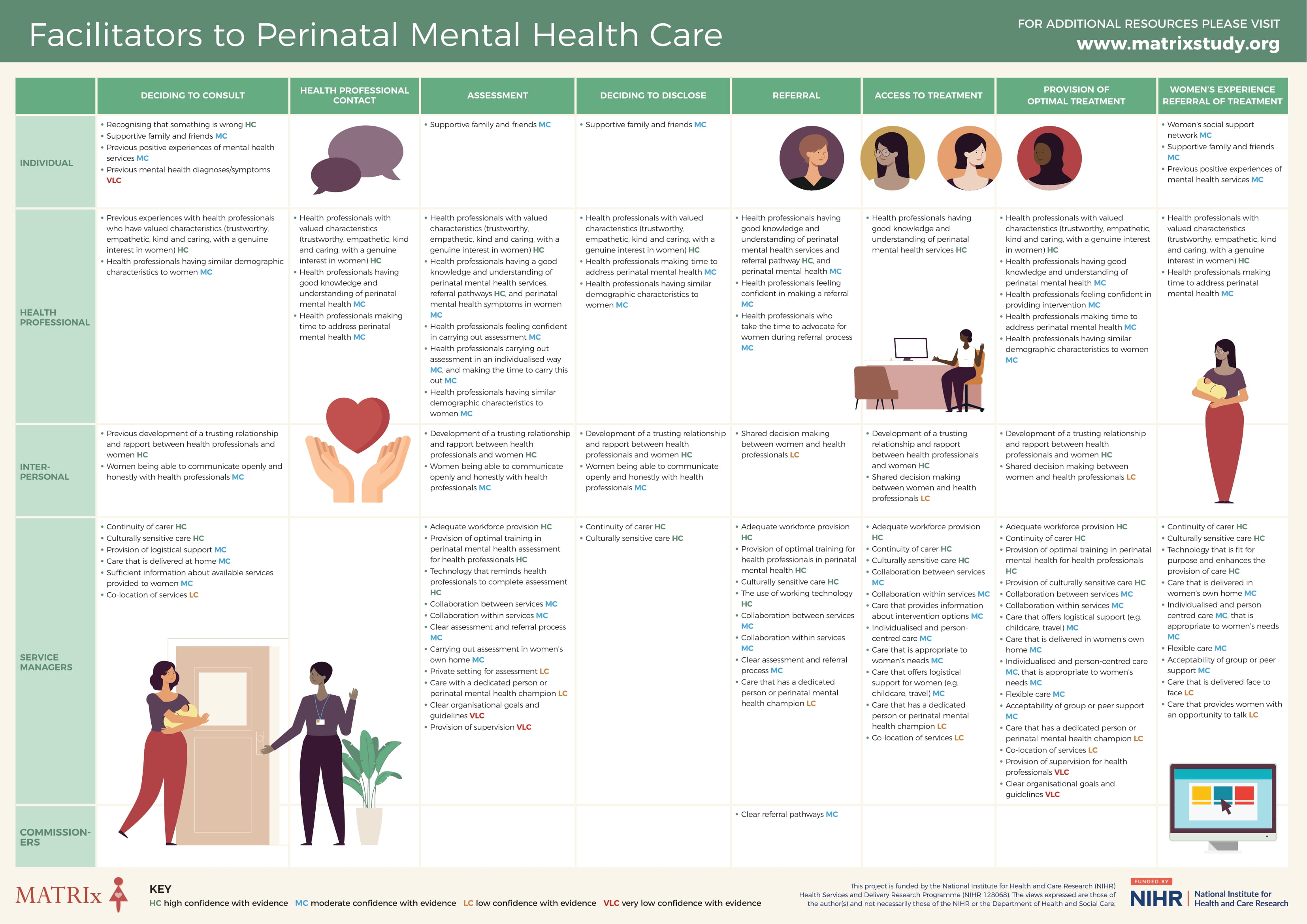


# *Supplementary Materials 12:* MATRIx recommendations for policy and practice

|  | Evidence | Recommendations |
| --- | --- | --- |
| Women | - Understanding women may believe that their symptoms are a normal part of motherhood*** which may lead to minimising symptoms*** or ignoring them*** - Understanding women may not fully understand the roles of each HPs meaning they may not feel comfortable talking with them about their symptoms*** - Understanding women may not want to disclose symptoms because of fears of being judged as a ‘bad mum’*** - Understanding women may not want to disclose symptoms because of fear of social services involvement**** - Understanding the presence of supportive family can be a facilitator to PMH care access*** - Understanding that women recognising something is wrong is a facilitator to PMH care access**** - Understanding previous positive experiences of mental health services is a facilitator to PMH care access*** - Services that provide childcare***, flexible timing of appointments***, and easily accessible location/home delivery of care/treatment*** - Understanding that not being financially stable***, or being a refugee or an immigrant**** can be a barrier to accessing care - Understanding additional personal difficulties, such as unemployment may prevent PMH care access*** | ^1^Development of information aimed at increasing awareness of PMI such as (1) infographics/leaflets disseminated through maternity services, primary care, third sector organisations (e.g. NCT), and antenatal classes (2) short animations & videos disseminated via social media on:   - Symptoms of different PMI - How they are common, and when to seek help - Causes - How to access professional support - Services available - Maternity professionals and their role in PMH care - Myth busters on social services and medication   ^2-4^Provision of care that meets women’s needs – is flexible, easy to access and provides childcare  ^4^A fair welfare and economic system that ensures that no one is living in poverty or in financial hardship |
| HPs | - Having a reasonable workload to ensure there is time to address women’s concerns**** - Works collaboratively with other HPs and other services*** - Communicates clearly and openly with other HPs*** - Validating women’s symptoms**** - Having the knowledge to understand different PMH difficulties*** - Recognising help-seeking*** - Has received adequate training****and therefore has good knowledge about PMI*** and other services and referral pathways**** - Feels confident in addressing PMH concerns*** - Caring HPs who show a genuine interest in women and who are trustworthy, non-judgemental, empathetic and warm**** | ^2-4^Provision of an adequate number of workers to meet women’s needs (see below).  ^2^Multidisciplinary meetings, co-location, encouragement of a culture of team working, joint working, sharing knowledge, and approachability ^a(see Box 3)^  ^2&4^ Implementation of PMH good practice guides(1) which cover:   - Symptoms of PNMI - Communication skills when discussing PNMI - What to do if a woman discloses PMH difficulties - Training opportunities - Links to further resources - Case studies with examples of good practice   ^5^Participates in CPD activities related to PMH including participating in high quality training (see below). Consider HPs receiving accreditation for participating in training(2)  ^2&4^Recruitment of staff positive interest and attitude towards providing high quality care to women. Consider HP receiving accreditation for providing high quality care, team working, and clear communication(2). |
| Interpersonal | - Resources available to break down language barriers such as translators or Language Line**** - Opportunities to form trusting relationships between women and HPs**** - Opportunities for open and honest communication*** | ^2^Recruit translators or form partnerships with other agencies that can provide additional support (e.g. translation services, interpreters(2)Click or tap here to enter text.) to translate infographics/leaflets into local languages^b^ and to act as an interpreter at appointments if women feel comfortable.  ^2^Investment in live translation tools or telephone interpreting such as Language Line.  ^2-4^Provision of continuity of carer across the care pathway^c^ |
| Service managers | - Recruitment of a multi-disciplinary team with enough staff to meet service users’ needs**** - Provision of continuity of carer**** - Clear assessment and referral processes*** - High quality staff training for all people working within a service, that is provided face-to-face, is time-protected, and covers PMH symptoms, treatment, cross-cultural presentations of PMH, referral pathways and available services and communication skills**** - Easy-to-use technology that is compatible with other technology systems used in other services**** - Provision of culturally sensitive care**** that is individualised***, flexible***, appropriate to women’s needs***. Provision of care should ideally be delivered face-to-face***, provides logistical support*** or is carried out in a home setting***. Furthermore, peer support is valued by some women too and should be considered*** - Clearly worded assessment tools*** - Assessment delivered in an individualised manner with discussion and adequate time given*** | ^2-4^Ensure an adequate workforce to meet women’s needs by utilising a workforce planning tool(3) and considering if there are a sufficient number of people in each of the key roles (psychiatrist, pharmacist, nurse, psychologist, occupational therapist, support staff, admin, peer support). Ensure a diverse workforce(4)  ^2-4^Clear & easily accessible guidelines on where to refer women to depending on their need. Development of one referral form that can be uploaded and amended, discussed at multidisciplinary team meetings^d^. Encouragement of a workspace that involves co-location, a culture of team working, sharing knowledge, approachability.  ^2&4^Provision of training for all people working in a health service. Consider the use of simulation training^e^. Training should:   - Be ring fenced/time protected - Provide accreditation, matched to competencies and appropriate to level of involvement - Be expected for all health services staff who have contact with perinatal women - Be interactive and provided by a knowledgeable person or network - Where relevant be face-to-face   Training should cover:   - Symptoms of PNMI – not just depression - How to talk about PMH, what questions to ask, language use - How and where to refer to - Diverse family structures - Vulnerable groups - Health inequalities - Lived experiences - Trauma informed care - Cross cultural presentations of mental illness - How to engage women from diverse backgrounds ^f^   ^2^Encourage co-production or user experience testing of technology to ensure ease of usability and integration into the workflow. Employment of a liaison person who has access to all systems to bridge the gap between different services.  ^6^Using compatible IT systems for easy access to information.  ^2&4^ Encourage co-production of care^f^. Collaborate with organisations such as The Motherhood Group to ensure care is culturally appropriate. Provide peer support to women who feel it would benefit them. Consider provision of home visits for care and deliver care face-to-face. If home delivery is not possible, ensure practical support is available such as childcare.  ^2&4^Use easy to understand assessment tools. Collaborate with organisations such as The Motherhood Group to ensure cultural appropriateness. Design or update assessment tools that use pictures alongside words for use with women whose English speaking and understanding is limited^h^. Ensure HPs have enough time to do this by creating an adequate workforce (see above)  ^5^Provide assessment in a woman-centred way. Explain questions or wording that women are not clear about. Clearly discuss results with women and explain next steps. |
| Commissioners | - Provision of adequate financial resources to ensure service managers can: - Recruit a multi-disciplinary team with enough staff to meet service users’ needs**** - Provide high-quality, time protected staff training to all staff**** - Provide continuity of carer**** - Provide resources that break down language barriers such as translators or Language Line**** - Provide an adequate number of appropriate services that women can be referred to in a timely manner**** - Reduction of the changeover of technology when new commissioners join, and encouragement of technology use that is compatible with other systems**** - Provide individualised, woman-centred care**** - Designing clear referral pathways^n^*** - Designing integrated care – Ensure collaboration within and between services*** - A clear and easy to access funding structure for commissioners and service managers | ^4&6^To provide services that meet the needs of the population, commissioners must(5):   - Have a good knowledge of population and the healthcare need in question. Therefore, training on PMH should be mandatory for at least one commissioner in each PCN, ICS or Health Board (see recommended training above) - Have access to high quality evidence e.g. the development of PMH information guide^i^/videos that covers:   - Symptoms of PNMI   - Impact on women and their families   - Barriers to women getting care they need and how to overcome these   - Effective care and treatment   - Examples of good practice - Engage with people with lived experience – services should be co-produced with those who have lived experience(6); see Box 3 – f).   ^4&7^Continued policy support from NHS England, and NHS related to PMH care, such as the publication of the Five Year Forward View(7) and Long Term Plan(8), NHS England, and Delivering Effective Services(9) report for NHS Scotland. |
| Government and regulatory bodies | - Support for refugee or immigrant women to be able to access care without being penalised (e.g., through deportation, through charging systems)*** - Adequate financial support for those who are not eligible for free healthcare*** - A clear and easy to access funding structure for commissioners and service managers, equality of funding distribution and adequate funding provision to ensure service needs are met*** | ^4^Free healthcare for all at the point of access^j^.  Suspension of NHS charging regulations until a full independent review of their impact on individual and public health, simplification of charging criteria and exemptions and safeguards to protect vulnerable patients and ensure they are not denied the care they are entitled to, is carried out^k^  ^4^A fair welfare and economic system that ensures that no one is living in poverty or in financial hardship^l^  ^4^The provision of a comprehensively researched and adequate budget provided to the Department of Health and Social Care, Health and Social Care Directorates and so all healthcare needs for that financial year can be met.  ^4^Where possible, reduction of in-year funding changes in England so local areas know exactly how much they can spend at the start of the year(10). |
| Society | - Less societal stigma related to mental health**** | ^4&7^NHS Mental Health Campaign focused on stigma reduction^m^ |

Note. **** = high confidence with evidence; *** = moderate confidence with evidence; ** = low confidence with evidence; * = very low confidence with evidence (based on CERQUAL Ratings)

1. Recommended development by third party organisations in collaboration with NIHR Applied Research Collaboration (ARC) PMH Themes Perinatal Mental Health Network Scotland National Managed Clinical Network, and Royal Colleges
2. Recommended development by service managers
3. Recommended *financial support* from commissioners
4. Recommended policy for government
5. Recommendation for HP
6. Recommendation for commissioners
7. Recommendation for NHS England

**Further information related to recommendations in Supplementary Materials 12**

1. The Greater Manchester Perinatal Parent Infant Mental Health Model of care works within an integrated system, making sure all services work together, preventing silo style working(11)
2. ACACIA Family support provide pre and postnatal depression support services. They have translated patient information into multiple languages (Arabic, Bengali, Chinese, French, Hindu, Polish, Punjabi, Romanian, Samoan & Urdu)(12)
3. The Tower Team based in the Tower Hamlets, London is a high-risk caseload midwifery team that works closely with the perinatal mental health team and the consultant obstetrician for mental health at St. Thomas’ hospital. The Tower Team offers continuity of care for women with severe mental illness from their maternity booking appointment, throughout pregnancy, intrapartum and for up to 28 days postpartum(13)
4. Perinatal Mental Health Service at South West London and St Georges Mental Health NHS Trust;
5. Brighton and Sussex University Hospitals NHS Trust provide Perinatal Mental Health Simulation Training on the identification and management of common perinatal mental health problems using actors and ‘real-life’ settings.
6. The Motherhood Group provide training related to engaging with Black women(14)
7. One example of a successful co-produced service is the co-production of perinatal mental health services in Ealing, Hammersmith, Fulham & Hounslow. There was strong engagement with lived experience experts from the start(13)
8. For example, How are you feeling screening tools by Abi Sobowale (Sheffield South West NHS Trust)
9. A guide for commissioning services is available for London. However, this was published in 2017 and needs updating in line with this conceptual framework(13)
10. Despite the NHS being free for UK residents, there are NHS charging regulations in place for those who are not residents of the United Kingdom. NHS charging regulations have a large negative impact on pregnant and postnatal women, in terms of their mental health(15) – increasing stress and anxiety, their vulnerability to domestic violence(16) and maternal deaths that may have been prevented through access to antenatal care(17,18). Furthermore, Public Health England has identified NHS charging for maternity care as one of the key issues that exacerbates poorer health outcomes for women and babies of colour(19).
11. This recommendation is in line with: (a) a joint statement set out by the Royal College of Physicians, the Royal College of Paediatrics and Child Health, the Royal College of Obstetricians and Gynaecologists and the Faculty of Public Health in 2018, calling for a suspension of NHS Charging(20); (b) a statement from the Academy of Medical Royal Colleges in 2019 released a statement calling for the suspension of the NHS charging regulations until a full independent review on individual and public health is carried out(21); (c) a statement from the Royal College of Paediatrics and Child Health calling for an end to NHS charging due to its adverse effects on child health and wider public health(22); (d) a report from Maternity Action calling for the immediate suspension of charging for NHS maternity care given the deterrent effect on women’s access to maternity care(23).

The UK government, under Article 25 of international human rights law, has a legal obligation to ensure “Everyone has the right to a standard of living adequate for the health and well-being of [them]self and of [their] family, including food, clothing, housing and medical care and necessary social services, and the right to security in the event of unemployment, sickness, disability, widowhood, old age or other lack of livelihood in circumstances beyond [their] control” and to ensure “Motherhood and childhood are entitled to special care and assistance. All children, whether born in or out of wedlock, shall enjoy the same social protection.

1. Based on research suggests public mental health campaigns can increase knowledge about mental illness and improve attitudes about people with mental illness(24–27)
2. The Future NHS Platform for National Perinatal Mental Health provides examples of pathways & system delivery models: Maternal mental health services-> MMHS Resources-> 3. Pathways & system delivery models

# *Supplementary Materials 13:* References

1. University of Liverpool. Supporting mental healthcare in a maternity and neonatal setting: Good practice guide and case studies. London: NHS England and NHS Improvement; 2021.

2. Powell BJ, Waltz TJ, Chinman MJ, Damschroder LJ, Smith JL, Matthieu MM, et al. A refined compilation of implementation strategies: Results from the Expert Recommendations for Implementing Change (ERIC) project. Implement Sci. 2015;10(1).

3. Future NHS. Mental Health Long Term Plan Analytical Tool [Internet]. 2021. Available from: https://future.nhs.uk/system/login?nextURL=%2Fconnect.ti%2FMHLTPat%2Fgrouphome

4. NHS North Region. Suggested PMH Workforce Planning KLOEs developed by North region [Internet]. National Perinatal Mental Health Workspace - NHS Future Platform. 2021 [cited 2021 Oct 22]. Available from: https://future.nhs.uk/PMHSCN/grouphome

5. Moreton R, Collinson B, Sandhu J. Working with commissioners and policy-makers: Workforce development and multiple disadvantage. 2021.

6. Woodall J, Davison E, Parnaby J, Hall AM. A Meeting of Minds: How co-production benefits people, professionals and organisations. 2019.

7. NHS England. NHS England » The NHS Five Year Forward View. NHS England. 2014.

8. NHS England. The NHS Long Term Plan. 2019.

9. NHS Scotland. Delivering Effective Services: Needs assessment and service recommendations for specialist and universal perinatal mental health services [Internet]. 2019. Available from: https://www.gov.scot/binaries/content/documents/govscot/publications/strategy-plan/2019/03/delivering-effective-services-needs-assessment-service-recommendations-specialist-universal-perinatal-mental-health-services/documents/delivering-effective-services

10. The King’s Fund. How funding flows in the NHS. 2020.

11. Greater Manchester Combined Authority. Greater Manchester Perinatal and Parent Infant Mental Health Service: Championing 1001 Critical Days. 2020.

12. ACACIA Family Support. Help for Black, Asian and Minority Ethnic Mums / Dads and People of Colour [Internet]. Pre and postnatal depression support services. 2021. Available from: https://www.acacia.org.uk/bame/mums-dads/#

13. NHS London Clinical Networks. Perinatal mental health services for London: Guide for commissioners. 2017.

14. The Motherhood Group. Understanding Diverse Motherhood: Workshop & Training [Internet]. 2021. Available from: https://www.themotherhoodgroup.com/bmmhwstakeholdertoolkit

15. Feldman R. What Price Safe Motherhood? Charging for NHS Maternity Care in England and its Impact on Migrant Women. Maternity Action; 2018.

16. Harris S, Hardwick J. A Vicious Circle: The relationship between NHS Charges for Maternity Care, Destitution, and Violence Against Women and Girls. Maternity Action; 2019.

17. Nullums LB, Rustage K, Hargreaves S, Friedland JS, Miller A, Hiam L. Access to healthcare for people seeking and refused asylum in Great Britain: A review of evidence. Equality and Human Rights Commission; 2018.

18. Knight M, Bunch K, Tuffnell D, Shakespeare J, Kotnis R, Kenyon S, et al. Saving Lives, Improving Mothers’ Care – Lessons Learned to Inform Maternity Care from the UK and Ireland Confidential Enquiries into Maternal Deaths and Morbidity 2015-17. MBRRACE-UK; 2019 p. 28.

19. Public Health England. Maternity high impact area: Reducing the inequality of outcomes for women from Black, Asian and Minority Ethnic (BAME) communities and their babies. 2020.

20. Royal Colleges. Royal colleges support suspension of NHS overseas visitor charges pending review [Internet]. Royal College of Physicians; 2018. Available from: https://www.rcplondon.ac.uk/news/royal-colleges-support-suspension-nhs-overseas-visitor-charges-pending-review

21. Academy of Medical Royal Colleges. NHS charges to overseas visitors regulations: A statement from the Academy of Medical Royal Colleges. 2019.

22. Royal College of Paediatrics and Child Health. Access to healthcare for migrant and undocumented children - position statement. 2021.

23. Maternity Action. Breach of Trust: A review of implementation of the NHS charging programme in maternity services in England. 2021.

24. Dietrich S, Mergl R, Freudenberg P, Althaus D, Hegerl U. Impact of a campaign on the public’s attitudes towards depression. Health Educ Res. 2010;25(1):135–50.

25. Jorm AF, Christensen H, Griffiths KM. The impact of beyondblue: The national depression initiative on the Australian public’s recognition of depression and beliefs about treatments. Aust N Z J Psychiatry. 2005;39(4):248–54.

26. Makowski AC, Mnich EE, Ludwig J, Daubmann A, Bock T, Lambert M, et al. Changes in beliefs and attitudes toward people with depression and schizophrenia - results of a public campaign in Germany. Psychiatry Res. 2016;237:271–8.

27. Sampogna G, Bakolis I, Evans-Lacko S, Robinson E, Thornicroft G, Henderson C. The impact of social marketing campaigns on reducing mental health stigma: Results from the 2009–2014 Time to Change programme. Eur Psychiatry. 2017;40:116–22.
